# Effects of Hormone Therapy on Cognition and Mood in Recently Postmenopausal Women: Findings from the Randomized, Controlled KEEPS–Cognitive and Affective Study

**DOI:** 10.1371/journal.pmed.1001833

**Published:** 2015-06-02

**Authors:** Carey E. Gleason, N. Maritza Dowling, Whitney Wharton, JoAnn E. Manson, Virginia M. Miller, Craig S. Atwood, Eliot A. Brinton, Marcelle I. Cedars, Rogerio A. Lobo, George R. Merriam, Genevieve Neal-Perry, Nanette F. Santoro, Hugh S. Taylor, Dennis M. Black, Matthew J. Budoff, Howard N. Hodis, Frederick Naftolin, S. Mitchell Harman, Sanjay Asthana

**Affiliations:** 1 School of Medicine and Public Health, University of Wisconsin, Madison, Wisconsin, United States of America; 2 Geriatric Research, Education and Clinical Center, William S. Middleton Memorial Veterans Hospital, Madison, Wisconsin, United States of America; 3 Wisconsin Alzheimer’s Disease Research Center, Madison, Wisconsin, United States of America; 4 Department of Biostatistics and Medical Informatics, University of Wisconsin, Madison, Wisconsin, United States of America; 5 Department of Neurology, Emory University School of Medicine, Atlanta, Georgia, United States of America; 6 Emory Alzheimer’s Disease Research Center, Atlanta, Georgia, United States of America; 7 Preventive Medicine, Brigham and Women’s Hospital, Harvard Medical School, Boston, Massachusetts, United States of America; 8 Departments of Surgery and Physiology & Biomedical Engineering, Mayo Clinic, Rochester, Minnesota, United States of America; 9 Utah Foundation for Biomedical Research, Salt Lake City, Utah, United States of America; 10 Obstetrics & Gynecology, University of California at San Francisco, San Francisco, California, United States of America; 11 Obstetrics & Gynecology, Columbia University College of Physicians and Surgeons, New York, New York, United States of America; 12 VA Puget Sound Health Care System, Tacoma, Washington, United States of America; 13 Division of Metabolism, Endocrinology and Nutrition, University of Washington, Tacoma, Washington, United States of America; 14 Neuroscience and Obstetrics & Gynecology, Albert Einstein College of Medicine, Bronx, New York, United States of America; 15 Obstetrics & Gynecology, University of Colorado School of Medicine, Aurora, Colorado, United States of America; 16 Obstetrics & Gynecology, Yale University School of Medicine, New Haven, Connecticut, United States of America; 17 Epidemiology & Biostatistics, University of California at San Francisco, San Francisco, California, United States of America; 18 Division of Cardiology, Los Angeles Biomedical Research Institute at Harbor–UCLA Medical Center, Torrance, California, United States of America; 19 Atherosclerosis Research Unit, University of Southern California, Los Angeles, California, United States of America; 20 Obstetrics & Gynecology, New York University School of Medicine, New York, New York, United States of America; 21 Kronos Longevity Research Institute, Phoenix, Arizona, United States of America; 22 Division of Endocrinology, Phoenix VA Medical Center, Phoenix, Arizona, United States of America; University of Cambridge, UNITED KINGDOM

## Abstract

**Background:**

Menopausal hormone therapy (MHT) reportedly increases the risk of cognitive decline in women over age 65 y. It is unknown whether similar risks exist for recently postmenopausal women, and whether MHT affects mood in younger women. The ancillary Cognitive and Affective Study (KEEPS-Cog) of the Kronos Early Estrogen Prevention Study (KEEPS) examined the effects of up to 4 y of MHT on cognition and mood in recently postmenopausal women.

**Methods and Findings:**

KEEPS, a randomized, double-blinded, placebo-controlled clinical trial, was conducted at nine US academic centers. Of the 727 women enrolled in KEEPS, 693 (95.3%) participated in the ancillary KEEPS-Cog, with 220 women randomized to receive 4 y of 0.45 mg/d oral conjugated equine estrogens (o-CEE) plus 200 mg/d micronized progesterone (m-P) for the first 12 d of each month, 211 women randomized to receive 50 μg/d transdermal estradiol (t-E2) plus 200 mg/d m-P for the first 12 d of each month, and 262 women randomized to receive placebo pills and patches. Primary outcomes included the Modified Mini-Mental State examination; four cognitive factors: verbal learning/memory, auditory attention/working memory, visual attention/executive function, and speeded language/mental flexibility; and a mood measure, the Profile of Mood States (POMS). MHT effects were analyzed using linear mixed-effects (LME) models, which make full use of all available data from each participant, including those with missing data. Data from those with and without full data were compared to assess for potential biases resulting from missing observations. For statistically significant results, we calculated effect sizes (ESs) to evaluate the magnitude of changes.

On average, participants were 52.6 y old, and 1.4 y past their last menstrual period. By month 48, 169 (24.4%) and 158 (22.8%) of the 693 women who consented for ancillary KEEPS-Cog were lost to follow-up for cognitive assessment (3MS and cognitive factors) and mood evaluations (POMS), respectively. However, because LME models make full use all available data, including data from women with missing data, 95.5% of participants were included in the final analysis (*n* = 662 in cognitive analyses, and *n* = 661 in mood analyses). To be included in analyses, women must have provided baseline data, and data from at least one post-baseline visit. The mean length of follow-up was 2.85 y (standard deviation [SD] = 0.49) for cognitive outcomes and 2.76 (SD = 0.57) for mood outcomes. No treatment-related benefits were found on cognitive outcomes. For mood, model estimates indicated that women treated with o-CEE showed improvements in depression and anxiety symptoms over the 48 mo of treatment, compared to women on placebo. The model estimate for the depression subscale was −5.36 × 10^−2^ (95% CI, −8.27 × 10^−2^ to −2.44 × 10^−2;^ ES = 0.49, *p* < 0.001) and for the anxiety subscale was −3.01 × 10^−2^ (95% CI, −5.09 × 10^−2^ to −9.34 × 10^−3^; ES = 0.26, *p* < 0.001). Mood outcomes for women randomized to t-E2 were similar to those for women on placebo. Importantly, the KEEPS-Cog results cannot be extrapolated to treatment longer than 4 y.

**Conclusions:**

The KEEPS-Cog findings suggest that for recently postmenopausal women, MHT did not alter cognition as hypothesized. However, beneficial mood effects with small to medium ESs were noted with 4 y of o-CEE, but not with 4 y of t-E2. The generalizability of these findings is limited to recently postmenopausal women with low cardiovascular risk profiles.

**Trial Registration:**

ClinicalTrials.gov NCT00154180 and NCT00623311

## Abstract 2

In a randomized, controlled trial, Carey Gleason and colleagues examine the effects of hormone therapies on cognitive and mood outcomes in recently postmenopausal women.

## Editors' Summary

**Background:**

Menopause (“change of life”)—the time in a woman’s life when her menstrual periods stop—is a normal part of aging and usually occurs around the age of 50. In the years before menopause (the menopausal transition or perimenopause), the levels of estrogen and progesterone—sex hormones produced by the ovaries that prepare the woman’s body every month for a possible pregnancy—go up and down irregularly. This variation in hormone levels changes the frequency and characteristics of a woman’s periods but can also cause hot flashes (feeling hot on and off during the day), night sweats, vaginal dryness, bone thinning, and mood swings. Some women sail through menopause without experiencing any of these symptoms, but for other women menopausal symptoms, which can continue for several years after menopause, can be debilitating. For these women, menopausal hormone therapy (MHT, previously known as hormone replacement therapy, or HRT)—treatment with various combinations and types of estrogen and progesterone—can be prescribed during or after the menopausal transition to manage their troublesome symptoms.

**Why Was This Study Done?:**

Although MHT has helped many women deal with their menopausal symptoms, it can increase a woman’s risk of heart disease, stroke, blood clots, and breast cancer. There is also some evidence that MHT increases the risk of cognitive decline (decline in thinking, language, memory, understanding, and judgment) and dementia in women who start taking MHT after the age of 65. However, other evidence suggests that MHT might enhance cognition and mood if it is given at menopause rather than later. In this randomized, double-blinded, placebo-controlled clinical trial (the KEEPS-Cog trial, an ancillary study of the Kronos Early Estrogen Prevention Study, which examined the effect of MHT on cardiovascular health), the researchers investigate the effects of up to four years of MHT on cognition and mood in recently postmenopausal women living in the US. A randomized, placebo-controlled clinical trial compares the outcomes of participants assigned an active intervention or a placebo (dummy) intervention through the play of chance; in a double-blinded trial, neither the researchers nor the participants know who is receiving which treatment until the trial ends.

**What Did the Researchers Do and Find?:**

In the KEEPS-Cog trial, 220 healthy recently postmenopausal women took an estrogen pill every day and a progesterone pill for the first 12 days of each month, 211 women wore an estradiol patch (transdermal estradiol) and took a progesterone pill for the first 12 days of each month, and 262 women received placebo patches and pills for up to four years (the average follow-up was a little less than three years). The researchers assessed the trial participants for their overall cognitive health using an instrument called the Modified Mini-Mental State Examination, for four specific cognitive functions using several established instruments, for depression symptoms using the Beck Depression Inventory, and for mood using the Profile of Mood States instrument at baseline and at 18, 36, and 48 months. Statistical analysis of the data collected indicates that, during the trial, there were no treatment-related effects on cognition or depression symptoms. However, women treated with estrogen pills and progesterone (but not those treated with estradiol patches and progesterone) showed improvements in some mood symptoms compared to women in the placebo group.

**What Do These Findings Mean?:**

Before starting their trial, the researchers hypothesized that, because the body handles different formulations and types of estrogen in different ways, transdermal estradiol but not oral estrogen would improve cognition and mood in recently postmenopausal women when compared to placebo. Notably, the findings suggest that MHT does not alter cognition as hypothesized and that oral rather than transdermal estrogen has a small to moderate beneficial effect on mood. Importantly, these findings provide no information about the effects of MHT beyond four years and, because most of the women in the study were white, well-educated, and at low risk of cardiovascular disease, may not be applicable to the general postmenopausal population of the US and of other countries. Moreover, because MHT improved menopausal symptoms in the women receiving hormones, the trial was not truly double-blinded. However, despite these and other study limitations, the researchers suggest that their findings could now be used to help women make more informed decisions about whether to use MHT to manage their menopausal symptoms.

**Additional Information:**

Please access these websites via the online version of this summary at http://dx.doi.org/10.1371/journal.pmed.1001833.

- The US National Institute on Aging provides detailed information for women about menopause (in English and Spanish) and about hormones and menopause
- The UK National Health Service Choices website also provides detailed information about menopause and about menopausal hormone therapy, including some personal stories
- The US Food and Drug Administration provides answers to common questions about menopause and hormones (in English and Spanish)
- MedlinePlus provides links to further resources and advice about menopause and about menopausal hormone therapy (in English and Spanish)
- More information about the KEEPS-Cog trial is available

## Introduction

Previously referred to as hormone replacement therapy (HRT), supplementation with estrogen and progesterone is prescribed during or after the menopausal transition for management of menopausal symptoms. There is emerging evidence that the cognitive and mood effects of menopausal hormone therapy (MHT) may vary depending on the timing of administration relative to menopause [1,2]. Specifically, it is hypothesized that MHT could potentially enhance cognition and mood if administered at menopause. While we did not test the long-term effects of MHT in the present study, others have theorized that MHT could reduce the risk of neurodegenerative disorders like Alzheimer disease, but only if initiated shortly after menopause—the “critical window” hypothesis [3,4]. Differences in the timing of MHT initiation could partly explain discrepant findings from prior clinical trials and observational studies. For example, results from trials involving recently postmenopausal women and several large observational studies including women initiating MHT shortly after menopause suggest that MHT has either a neutral or beneficial effect on cognition and mood [5–11]. In contrast, findings from several large studies, including the Women’s Health Initiative Memory Study (WHIMS), enrolling women aged 65 y and older indicated that both opposed and unopposed conjugated equine estrogens (CEE) were associated with adverse cognitive effects [12–17] and no mood benefits [16,17].

The Kronos Early Estrogen Prevention Study–Cognitive and Affective Study (KEEPS-Cog), an ancillary study of the Kronos Early Estrogen Prevention Study (KEEPS) [18–20], is a randomized trial funded by the US National Institutes of Health-Institute on Aging (NIH-NIA) designed to characterize the cognitive and mood effects of up to 4 y of MHT in recently postmenopausal women. The primary Kronos Early Estrogen Prevention Study (KEEPS) was designed to test the effects of early initiation of oral or transdermal MHT versus placebo on a surrogate measure of atherosclerotic progression, carotid artery intima-media thickness. Unique features of the KEEPS include exclusive enrollment of a large sample of recently postmenopausal women, within 3 y of their last menstrual period (LMP); evaluation of the efficacy of a low dose of oral CEE (o-CEE) and transdermal estradiol (t-E2) in a single study; cyclical administration of oral micronized progesterone (m-P; Prometrium); and examination of the effects of MHT on cognition and mood and related symptoms in non-depressed recently postmenopausal women.

Depending on the formulation and mode of delivery, MHT may exert differential cognitive and mood effects [21,22]. Among the key differences between t-E2 and o-CEE are the “first pass” hepatic metabolism occurring with oral estrogens before the drug reaches systemic circulation, and variations in the predominant form of estrogen in each preparation (17β estradiol in the transdermal product versus estrone in CEE [21,22]). We hypothesized that women randomized to t-E2 but not o-CEE would exhibit cognitive and mood improvements compared to women on placebo. Additionally, we explored the role of a genetic polymorphism potentially influencing response to MHT, *apolipoprotein E (APOE)* ε4 carrier status. Possessing one or two copies of the ε4 allele confers an increased risk for Alzheimer disease [23], and may influence estrogen’s effects on cognitive and mood outcomes [24].

Presented here are findings from the KEEPS-Cog. Building on findings in women initiating MHT during or shortly after the menopausal transition [5–11], the KEEPS-Cog tested the hypothesis that up to 4 y of MHT would improve cognition and mood when initiated in healthy recently postmenopausal women.

## Methods

### Design Overview

Women enrolled in the KEEPS [18–20], a randomized, placebo-controlled, double-blind trial, were approached for participation in the KEEPS-Cog. A description of the KEEPS’s recruitment and screening methods, inclusion/exclusion criteria, and participating sites was published previously [18–20]. The KEEPS-Cog was designed to evaluate the potential effects of up to 4 y of t-E2 or o-CEE therapy with cyclical m-P on cognitive and mood outcomes in healthy, non-hysterectomized recently postmenopausal women. The original protocol entailed 5 y of treatment. This was altered in the first year of the study, after reconsideration of study design. Specifically, analyses suggested that the length of follow-up could be shortened without compromising the power to detect changes in study outcomes.

The University of Wisconsin–Madison served as the coordinating center for KEEPS-Cog. Cognitive and mood data were collected at nine US sites. The research team based in Madison, Wisconsin, coordinated data collection, trained study personnel, ensured adherence to the KEEPS-Cog study protocol, and monitored data quality.

### Ethical Approval

All participants provided written informed consent. Institutional review boards (IRBs) at the participating sites and the University of Wisconsin approved study procedures. Specifically, the Western Institutional Review Board reviewed and approved activities led by the central KEEPS investigators and Phoenix KEEPS study site investigators. Central KEEPS and Phoenix protocol numbers were as follows: IRB—1058663, Western Institutional Review Board—20040792KEEPS, #10–02980, and MDBHAS #11–05383. The University of Wisconsin Health Science IRB reviewed and approved activities led by the KEEPS-Cog investigators (IRB number: H-2005-0059). Specific study site IRB approvals were obtained from the following institutions: Brigham and Women’s Hospital (IRB number: 2004-P-002144 BWH), Mayo Clinic (IRB number: 2241–04), Columbia University (IRB number: AAAA-8062), Yale University (IRB number: 0409027022), University of Utah (IRB number: 13257), Albert Einstein College of Medicine–Montefiore (IRB number: 04-08-213), University of California, San Francisco (IRB number: 10–02980), and University of Washington (UW IRB number: 26702 and VAPSHCS IRB number: 01048).

### Setting and Participants

**Fig 1:**
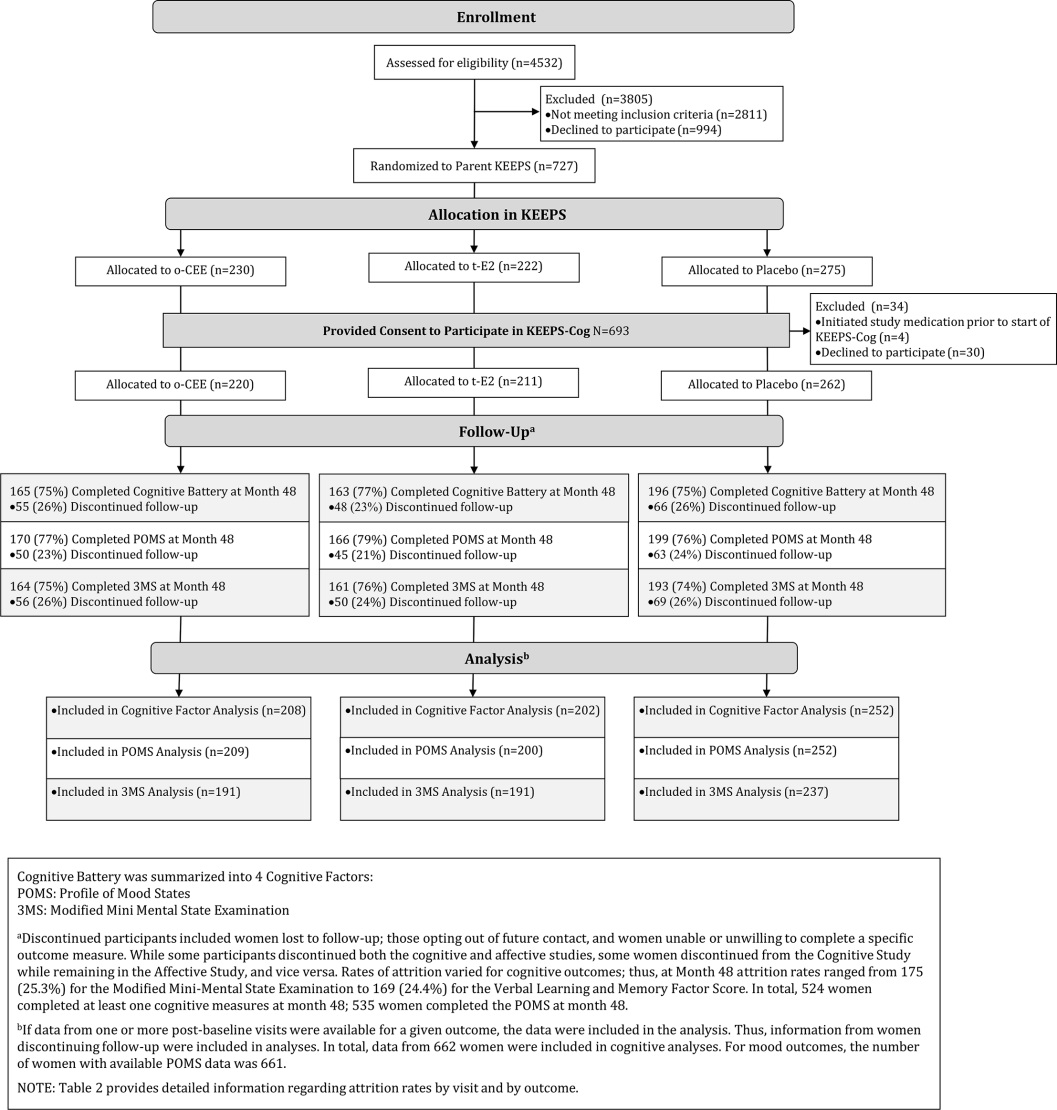
CONSORT diagram.

The KEEPS used print and radio advertisements to recruit and enroll 727 women; of these, 693 (95.3%) provided written informed consent for participation in the KEEPS-Cog. Thirty-four women from the full sample were ineligible, either because they had started study medications prior to the start of the ancillary study (*n* = 4) or because they declined to participate in the optional cognitive and mood assessments (*n* = 30). Enrollment occurred between August 2005 and July 2008, with final visits completed in 2012. Fig 1 depicts the number of women screened, enrolled, and followed. Entry into the KEEPS was limited to women in the late menopausal transition and early postmenopausal periods, defined as (1) being within 6–36 mo of their LMP and (2) having a plasma follicle stimulating hormone level of ≥35 ng/ml or serum estradiol level of ≤40 pg/ml. Additional eligibility criteria for the KEEPS involved risk for cardiovascular disease (CVD) (as determined by limits for baseline coronary artery calcium score), body mass index (BMI), blood pressure, fasting total cholesterol and glucose levels, and tobacco use [18–20]. Women were excluded if they had a history of severe psychiatric illness, including untreated major depression, but were allowed to enroll if they were taking anti-depressant medications to manage mild to moderate mood symptoms. If using MHT when enrolled, participants completed a 90-d washout period before study randomization.

In addition to meeting inclusion criteria for entry into the KEEPS [20], women in the KEEPS-Cog were cognitively healthy and free of clinical depression. Screening measures included the Mini-Mental State Examination (MMSE) [25] and Beck Depression Inventory, 2nd edition (BDI-II) [26]. For entry into the KEEPS-Cog, participants’ MMSE scores needed to be above age-, education-, ethnicity-, and race-adjusted cutoff scores suggestive of cognitive impairment [27–29]. To be inclusive, five women whose MMSE scores were below the typical cutoff of 24 of 30 points were included in analysis. For these women, MMSE scores were ≥22 and were determined to be related to language or educational differences rather than impaired cognition. To exclude women with clinical depression, women with screening BDI-II scores that exceeded 28/63 (the cutoff score for severe depression [26]) were excluded.

### Randomization and Interventions

Women were randomized in a ratio of 4:4:5, favoring placebo. A random number generator (Excel, Microsoft) was used to devise a randomization series, sequenced in blocks of 13. Two individuals, an unblinded officer at the KEEPS coordinating site and a study pharmacist, were aware of the treatment assignment; neither were study investigators. The randomization key was not available to study personnel. The order of randomization was incorporated into a KEEPS database, so that treatment allocation occurred when a study identification number was assigned.

Participants received (1) o-CEE (0.45-mg tablet daily) plus cyclical m-P (200-mg capsule daily for 12 d) and a placebo skin patch, (2) t-E2 (50-μg/d) plus cyclical m-P (200-mg capsule daily for 12 d) and a placebo tablet, or (3) all placebos (a placebo capsule, placebo tablet, and placebo patch). All women were asked (1) to take a study tablet from one bottle, once daily, (2) to take a capsule once daily from the second bottle for the first 12 d of each month, and (3) to apply a new study patch weekly.

KEEPS-Cog procedures were performed in conjunction with the parent study visits. Parent study visits occurred every 3 mo; cognitive and mood data were collected at four of the parent study visits: baseline, and months 18, 36, and 48. At the month 18 and 48 visits, cognitive and mood assessments occurred between days 12 and 30, when women took estrogen preparations (i.e., t-E2 or o-CEE) or matching placebos without m-P. To explore possible effects associated with administration of m-P, the month 36 assessment occurred during the first 12 d of the month, when women were taking both estrogen and progesterone, or corresponding placebos. Sample size estimates performed during study development suggested that allocation of 150 women per group would be adequate to detect differences in outcomes between groups.

### Primary Outcomes and Follow-Up

Primary outcomes were grouped into two categories: cognition and mood. Cognition was estimated with a global cognitive measure and four domain scores. Global cognition was evaluated with the Modified Mini-Mental State (3MS) examination [30], a 100-point expanded version of a widely used test, the MMSE [25]. The 3MS exam measures orientation, auditory registration, working memory, recall, language, and constructional skills consistent with the MMSE, and includes questions assessing personal information, verbal fluency, abstraction, and long-term recall. This test was selected for inclusion because it was the primary cognitive assessment used in the WHIMS [12–14].

In addition to measuring global cognition with the 3MS exam, we derived cognitive domain scores from a comprehensive battery of 11 cognitive tests that were summarized with factor analysis. In brief, we began the analysis with a total of 25 subscales (from 13 tests), and after iteratively examining competing models and overall fit, 17 subscales were selected for inclusion in the final factor analysis. Scores from subscales with a restricted distribution were excluded from the factor analysis. Ultimately, subscales from 11 of the 13 tests were included in the four factors. Tests and subscales included in the factors are as follows: (1) California Verbal Learning Test, 2nd edition [31], as administered in the Women’s Health Initiative Study of Cognitive Aging (WHISCA) [16,17]; (2) New York University Paragraph Recall [32]; (3) Benton Visual Retention Test [33]; (4) Controlled Oral Word Association Test–Phonemic Fluency [34]; (5) Controlled Oral Word Association Test–Category Fluency [34]; (6) Trail Making Test A [34]; (7) Trail Making Test B [34]; (8) Stroop Color Word Interference Test [35]; (9) Wechsler Adult Intelligence Scale, 3rd edition (WAIS-3) Letter-Number Sequencing [36]; (10) Wechsler Memory Scale, 3rd edition (WMS-3) Digit Span [37]; and (11) WAIS-3 Digit Symbol [36].

**Fig 2:**
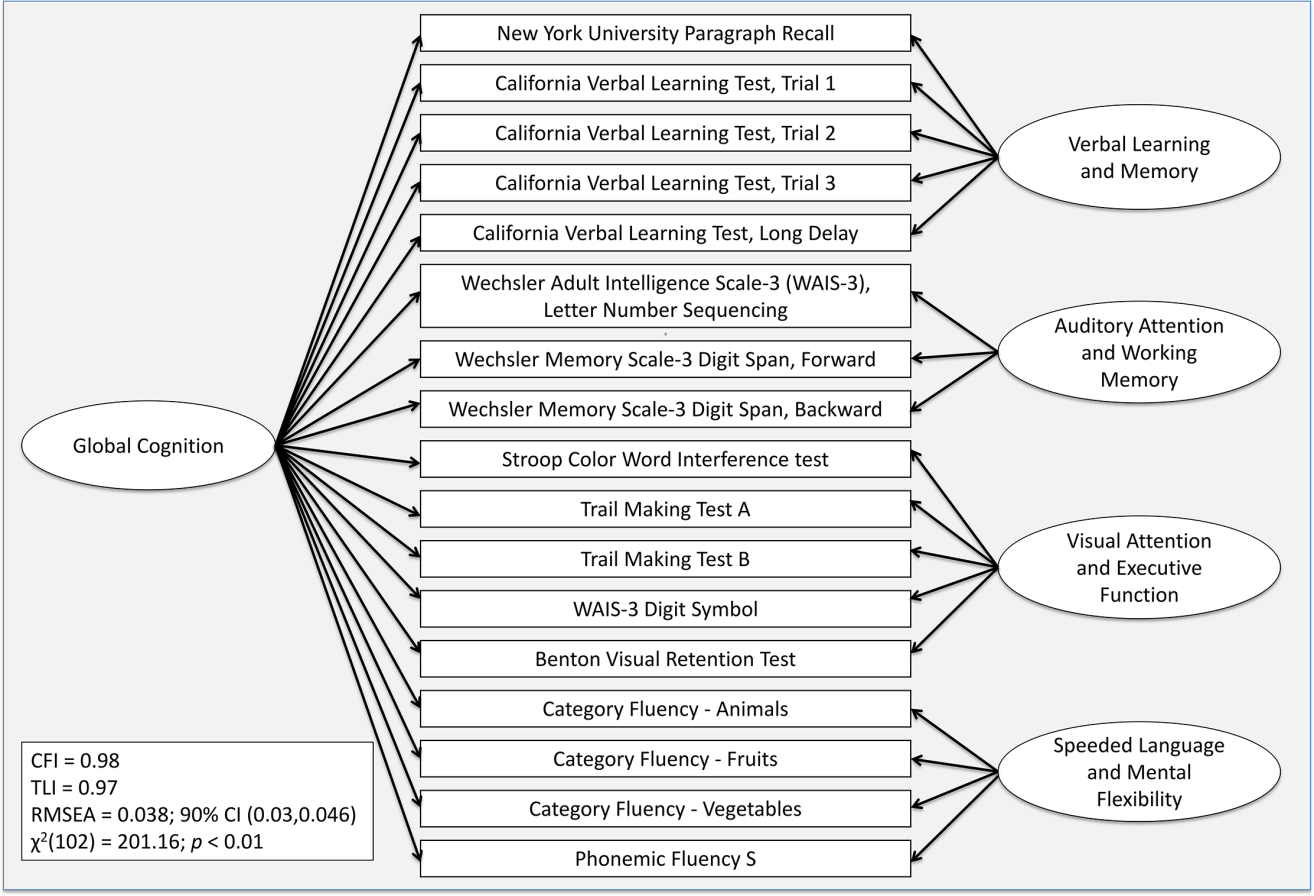
Factor model for the cognitive baseline data. CFI, comparative fit index; RMSEA, root mean square error of approximation; TLI, Tucker-Lewis index.

Fig 2 depicts the resultant four cognitive factors: verbal learning/memory, auditory attention/working memory, visual attention/executive function, and speeded language/mental flexibility. Baseline values for the cognitive factors, expressed as *T*-scores are provided in Table 1. By using multiple scores, summarized as factors, as opposed to individual test scores, we derived more reliable outcomes; the higher the reliability, the greater the power to detect true differences [38]. A full description of the cognitive battery, factor analysis, and summary scores is provided elsewhere [39].

**Table 1:** Baseline characteristics of the women in the KEEPS-Cog.

Characteristic	All Groups	Placebo	o-CEE	t-E2
	*n* = 693	*n* = 262	*n* = 220	*n* = 211
**Age (years), mean (SD), *n* = 687**	52.6 (2.6)	52.5 (2.5)	52.8 (2.7)	52.6 (2.6)
**Highest level of education completed, number (percent), *n* = 685**				
High school diploma, GED, or less	52 (7.5%)	25 (9.5%)	15 (6.8%)	13 (6.2%)
Some college/vocational	127 (18.3%)	44 (16.8%)	46 (20.9%)	37 (17.5%)
College graduate	277 (40.0%)	103 (39.3%)	92 (41.8%)	82 (38.9%)
Some graduate/professional school	31 (4.5%)	9 (3.4%)	8 (3.6%)	14 (6.6%)
Graduate or professional degree	197 (28.4%)	77 (29.4%)	58 (26.4%)	62 (29.4%)
**Self-reported race/ethnicity, number (percent), *n* = 658**				
Black	51 (7.4%)	22 (8.4%)	16 (7.3%)	13 (6.2%)
Asian	16 (2.3%)	5 (1.9%)	6 (2.7%)	5 (2.4%)
White	535 (77.2%)	201 (76.7%)	171 (77.7%)	163 (77.3%)
Hispanic	49 (7.1%)	20 (7.6%)	16 (7.3%)	13 (6.2%)
Other race/ethnicity	7 (1.0%)	2 (0.8%)	3 (1.4%)	2 (1.0%)
**MMSE** ^**a**^ **, mean score (SD) at screening, *n* = 683**	29.1 (1.9)	29.2 (1.5)	28.9 (2.6)	29.1(1.6)
**BDI-II** ^**b**^ **, mean score (SD) at screening, *n* = 683**	5.7 (5.4)	5.2 (5.1)	6.0 (6.0)	6.0 (5.3)
**BDI-II score, number (percent of full sample)**				
0–13 (suggests minimal depression)	612 (88.3%)	233 (88.9%)	187 (85.0%)	192 (91.0%)
14–19 (suggests mild depression)	58 (8.4%)	20 (7.6%)	21 (9.5%)	17 (8.1%)
20–28 (suggests moderate depression)	13 (1.9%)	5 (1.9%)	7 (3.2%)	1 (<1.0%)
Missing screening	10 (1.4%)	4 (1.5%)	5 (2.3%)	1 (<1.0%)
**BMI (kg/m** ^**2**^ **), mean (SD), *n* = 693**	26.3 (4.3)	26.6 (4.3)	26.1 (4.3)	26.1 (4.4)
**BMI (kg/m** ^**2**^ **), number (percent), *n* = 693**				
<20	58 (8.4%)	19 (7.3%)	21 (9.6%)	18 (8.5%)
20–24	215 (31.0%)	75 (28.6%)	73 (33.2%)	67 (31.8%)
25–29	289 (41.7%)	115 (43.9%)	87 (39.6%)	87 (41.2%)
30–34	114 (16.5%)	43 (16.4%)	37 (16.8%)	34 (16.1%)
≥35	17 (2.5%)	10 (3.8%)	2 (0.9%)	5 (2.4%)
***APOE* genotype, number (percent), *n* = 568**				
ε2/ε2	2 (0.3%)	2 (0.8%)	-	0
ε2/ε3	64 (9.3%)	23 (8.8%)	19 (8.8%)	22 (10.4%)
ε2/ε4	9 (1.3%)	2 (0.8%)	5 (2.3%)	2 (0.9%)
ε3/ε3	355 (51.5%)	145 (55.6%)	109 (50.2%)	101 (47.9%)
ε3/ε4	121 (17.6%)	43 (16.5%)	40 (18.4%)	38 (18.0%)
ε4/ε4	17 (2.5%)	11 (4.2%)	5 (2.3%)	1 (0.5%)
***APOE* ε4 carrier status, number (percent of 568), *n* = 568**	147 (25.9%)	56 (24.8%)	50 (28.1%)	41 (25.0%)
**Reported cigarette use, number (percent), *n* = 693**	45 (6.5%)	18 (6.9%)	13 (5.9%)	14 (6.6%)
**Baseline cardiovascular risk profile**				
Systolic blood pressure (mm Hg), mean (SD), *n* = 693	119.0 (15.0)	120.1 (14.5)	119.1 (14.8)	117.5 (15.7)
Diastolic blood pressure (mm Hg), mean (SD), *n* = 693	75.0 (9.2)	75.5 (9.5)	75.3 (8.3)	74.1 (9.6)
LDL (mg/dl), mean (SD), *n* = 688	110.9 (27.7)	111.0 (26.7)	111.0 (27.4)	110.8 (29.1)
HDL* (mg/dl), mean (SD), *n* = 688	72.0 (14.7)	70.1 (13.6)	72.9 (14.7)	73.4 (15.7)
Triglycerides (mg/dl), mean (SD), *n* = 688	87.9 (56.4)	92.9 (60.8)	84.6 (56.4)	85.1 (50.3)
HOMA-IR (mg/dl), mean (SD), *n* = 688	1.3 (2.4)	1.3 (1.4)	1.0 (0.9)	1.6 (3.8)
**Reported alcohol use** ^**c**^ **, number (percent), *n* = 693**	519 (74.9%)	198 (75.6%)	171 (77.7%)	150 (71.1%)
**Time since LMP (years), mean (SD), *n* = 691**	1.4 (0.7)	1.4 (0.7)	1.4 (0.8)	1.5 (0.7)
**Reported past use of MHT, number (percent), *n* = 693**	149 (21.5%)	48 (18.3%)	58 (26.4%)	43 (20.4%)
**Primary Mental Abilities** ^**d**^ **, mean score (SD), *n* = 661**	40.1 (10.2)	40.5 (9.9)	38.9 (11.2)	40.7 (9.4)
**3MS exam** ^**e**^ **, mean score (SD) at baseline, *n* = 619**	96.6 (4.3)	96.7 (4.4)	96.6 (4.3)	96.5 (4.0)
**Cognitive factor score** ^**f**^ **, mean standard score (SD) at baseline, *n* = 662**				
Verbal learning/memory	50.0 (8.7)	50.5 (8.7)	49.4 (8.3)	49.9 (8.9)
Auditory attention/working memory	50.0 (7.5)	50.0 (7.6)	48.9 (8.0)	51.1 (6.8)
Visual attention/executive function	50.0 (7.3)	49.9 (7.7)	49.9 (7.1)	50.2 (7.2)
Speeded language/mental flexibility	50.0 (7.9)	49.7 (7.8)	50.5 (7.8)	49.9 (8.2)
**POMS, mean score (SD) at baseline, *n* = 661**				
Tension-anxiety	6.4 (5.0)	6.2 (4.7)	6.9 (5.4)	6.3 (5.0)
Depression-dejection	5.5 (6.6)	4.5 (5.6)	6.4 (7.6)	5.7 (6.6)
Anger-hostility	5.3 (5.4)	4.8 (4.9)	5.4 (5.8)	5.8 (5.7)
Fatigue	6.5 (5.5)	6.2 (5.5)	6.6 (5.4)	6.8 (5.5)
Vigor	17.7 (6.4)	17.8 (6.7)	17.7 (6.2)	17.7 (6.2)
Confusion-bewilderment	5.0 (4.0)	4.9 (3.7)	5.1 (4.2)	4.9 (4.1)

^a^30-point scale; scores < 24/30 can suggest impairment.

^b^21-item scale; score ranges from 0 to 63, and a higher score indicates more symptoms of depression. Women were excluded from participation if they obtained a score of >28 at screening or baseline, suggesting severe depression.

^c^Defined as a binary variable (yes = 1; no = 0).

^d^50-point, multiple-choice test of vocabulary that provided estimate of intelligence.

^e^100-point scale; score < 85/100 can suggest impairment.

^f^Cognitive factor scores, produced by a factor analysis, have a metric similar to standardized scores. SDs, however, varied from factor to factor.

*p < 0.05.

GED, General Educational Development; HDL, high-density lipoprotein; HOMA-IR, homeostatic model assessment–insulin resistance; LDL, low-density lipoprotein; POMS, Profile of Mood States; SD, standard deviation.

The Profile of Mood States (POMS) [40] was used to examine potential treatment effects of MHT on specific affective states. In this questionnaire, women were asked to rate 65 adjectives using a five-point Likert scale to indicate the agreement between the adjective and their mood over the last week. Scores are summarized into six subscales. Validated as a measure of mood in postmenopausal women [41], the POMS was designed for use with non-depressed populations and is sensitive to subclinical variations in mood states. All six POMS subscales—tension-anxiety, depression-dejection, anger-hostility, fatigue, vigor, and confusion-bewilderment—were included in the analysis.

### Other Measures

The BDI-II [26] was used to screen for severe depression prior to enrollment and to monitor for adverse effects over the course of the study. The BDI-II is a 21-item inventory measuring attitudes and symptoms characteristic of depression. Participants were asked to select one of four statements for each symptom item, describing how they felt over the past 2 wk. Zero indicated the absence of a symptom; the numbers one through three corresponded to increasing symptom severity. A total score representing a sum value of responses was derived.

### Statistical Analysis

In the modified intent-to-treat analysis, participants with the baseline assessment and at least one post-baseline assessment were included in analyses in their original randomization group, regardless of drop-in or drop-out status in the parent KEEPS. Outcomes were selected based on their likelihood to detect group differences in response to MHT. Based on the number of women enrolled in the parent KEEPS, we expected a final sample size of 150 women per group, assuming an estimated 75% of women would be willing to participate in the ancillary KEEPS-Cog and an anticipated attrition rate of 4% per year. With 150 women per group, original power estimates suggested >80% power to detect group differences for the various outcome measures.

To examine the effect of treatment on cognition and mood throughout the study duration, MHT effects were analyzed using a linear mixed-effects (LME) [42] modeling approach; randomization group functioned as the grouping factor, and time since baseline functioned as a discrete fixed factor. Assuming that data are not missing due to systematic factors, LME models make full use of all available data from each participant [43], in contrast to models excluding participants with partial data.

To test the assumption that data were missing due to random factors, we compared the distribution of baseline variables in Table 1 for participants who completed the study and participants who were lost to follow-up. We also examined the pattern of missing data across variables, in order to detect possible patterns in the distribution of missing values related to various subgroup characteristics (e.g., age, race), and data appeared to be missing in a random manner. Additionally, LME model assumptions about linearity, normality, and the independence and homoscedasticity of errors were assessed graphically and analytically. Linear associations between predictors (treatment group) and cognitive and mood outcomes were verified by adding a quadratic term to models, and evaluating model fit. Treatment assignments were balanced by site; however, to address possible systematic differences in participants related to enrollment site, we included testing site as a random effect in all models. Age and education level were entered as fixed effects covariates, adjusting for baseline individual differences. Finally, for all models, the time factor included discrete values representing months elapsed since baseline, i.e., 0, 18, 36, and 48 mo. The fitted models are available in S3 Text.

The estimates provided by the LME models represent the interaction of time by treatment group (t-E2 and o-CEE), using the placebo group as reference. Our primary interest was to compare the effect of MHT versus placebo (t-E2 versus placebo and o-CEE versus placebo) on changes in cognitive performance and mood across time (where time represents months since baseline) after controlling for baseline age and education level. To determine the magnitude of the effects, we calculated an effect size (ES) by dividing the regression coefficient estimate by the baseline standard deviation of the outcome measures. The length of the study in a month metric was used to adjust the estimate. The estimate of ES can be interpreted as a Cohen’s *d* effect [44].

Our analyses of cognition and mood outcomes were performed separately, using the same analytic approach and *p*-value of 0.05, so that women with only one set of data could be included in the appropriate analysis. For example, mood analyses included data from the subset of women who provided data for the POMS outcomes, whether or not they provided data on the cognitive outcomes. To correct for multiple comparisons, we divided the *p*-value by the number of outcomes. For example, there were five cognitive outcomes (four domain factors and one global cognition score) and six POMS mood scores. Splitting the significance value of 0.05 equally between the five cognitive and six mood sub-domains resulted in a *p*-value of 0.01 for each of the two primary domains investigated (cognition and mood). We used the statistics software package R, version 2.13 (R Development Core Team; http://cran.r-project.org/), to explore the longitudinal data and model assumptions and SAS, version 9.3 (SAS Institute), to estimate the model parameters.

Prespecified secondary analyses examined the influence of participant characteristics and co-administration of m-P on MHT response. For example, the influence of *APOE* ɛ4 genotype, a genetic risk factor for dementia [23], on cognitive and mood outcomes was evaluated by adding a dichotomous variable (ɛ4 positive versus ɛ4 negative) to cognitive and mood outcome models as a moderator. The influence of time since LMP was examined in a model by adding in a continuous variable depicting time in months since LMP. In brief, we modeled the interaction of time since LMP by treatment group (o-CEE or t-E2), with placebo group as the reference. The effect of baseline mood as a moderator of cognitive response to MHT was also explored, using baseline BDI-II score in models examining cognitive effects over time. Baseline BDI-II score was evaluated as a continuous variable in models comparing the interaction of baseline BDI-II score by treatment group (o-CEE or t-E2), using the placebo group for reference in each model. Finally, to evaluate the effects of co-administration of m-P on cognitive and mood outcomes, change from baseline score obtained at month 36 was compared to change from baseline score obtained at month 48. Unlike other visits, month 36 assessments occurred during the first 12 d of the month, when women were taking combined estrogen and m-P or corresponding placebos.

## Results

The baseline characteristics of the 693 women who completed either cognitive testing or the POMS are presented by treatment group in Table 1. On average, participants were 52.6 y old and 1.4 y past their LMP at baseline. At screening, 71 (10.4%) of the 683 women for whom screening BDI-II scores were available reported symptoms suggestive of mild or moderately severe depression, although none were diagnosed with clinical depression; 58 women scored in the mildly depressed range (14 to 19 out of 63 points), and 13 women scored as having moderate depressive symptoms (20 to 28 out of 63 points). Consistent with published literature [45], the most common *APOE* allele was ɛ3 among 568 women genotyped, representing 78.8% of pooled alleles, while the ɛ4 allele represented 14.4% of the pooled alleles. Except for a small difference in serum high-density lipoprotein cholesterol levels, there were no statistically significant baseline differences detected among treatment groups in any of the variables measured. Overall, 34 women who participated in the KEEPS did not enroll in the KEEPS-Cog, and the KEEPS-Cog and KEEPS samples were similar in characteristics. In particular, cardiovascular risk profiles, smoking status, demographics, and past MHT profiles were highly consistent [19].

The number of women reporting a reduction in menopausal symptoms was similar in the KEEPS-Cog and the KEEPS. For example, in the KEEPS-Cog, vasomotor symptoms were common at baseline, with >40% of women reporting moderate to severe hot flashes. All women described a reduction in symptoms 6 mo after initiating study medications; however, women randomized to o-CEE and t-E2 demonstrated a statistically significantly greater reduction in symptoms, e.g., 28.6% of women in the placebo group described moderate to severe hot flashes at month 6, compared to 4.3% of women randomized to o-CEE and 6.7% of women randomized to t-E2. Overall, hot flashes were similarly controlled in the two MHT groups (see S1 Table). Notably, group differences in symptoms attenuated over time as hot flashes abated spontaneously for women in the placebo group.

Retention rates by visit and outcome are provided in Table 2. Women completing the baseline visit and at least one post-baseline visit were included in analyses. A total of 662 women were included in the final analytical sample for cognitive factors, and 619 for analysis of 3MS scores. The analyses of POMS outcomes included data from 661 women. The mean follow-up was 2.85 y (SD = 0.49) for cognitive outcomes and 2.76 y (SD = 0.57) for mood outcomes. Harman et al. [20] provide a full summary of adverse events and study withdrawal by randomization group for the KEEPS. For the KEEPS-Cog, 63 serious adverse events occurred in 56 women, 21 of whom were randomized to the o-CEE group, 17 to the t-E2 group, and 18 to the placebo group. Altogether, six cases of breast cancer occurred in women enrolled in the KEEPS-Cog (three in the o-CEE group, two in the t-E2 group, and one in the placebo group). Six women in the KEEPS-Cog experienced cardiac or cerebrovascular events. Specifically, in the o-CEE group, one woman experienced a transient ischemic attack. In the t-E2 group, one woman reported a suspected stroke (later determined not to be a stroke). Two cases of venous thrombotic disease occurred (one in the t-E2 group and one in the placebo group). Two women on o-CEE experienced a major depressive episode. No women developed a cognitive disorder while in the study.

**Table 2:** Retention rates by visit and outcome.

Test	Month 18		Month 36		Month 48		Final Analytic Sample	
	*N*	Retention (Percent)	*N*	Retention (Percent)	*N*	Retention (Percent)	*N*	Retention (Percent)
**3MS exam**								
Placebo	220	84.0%	200	76.3%	193	73.7%	237	90.5%
o-CEE	186	84.5%	173	78.6%	164	74.5%	191	86.8%
t-E2	172	81.5%	161	76.3%	161	76.3%	191	90.5%
Total sample	578	83.4%	534	77.1%	518	74.7%	619	89.3%
**Cognitive factor verbal learning/memory**								
Placebo	222	84.7%	203	77.5%	196	74.8%	252	96.2%
o-CEE	188	85.5%	174	79.1%	165	75.0%	208	94.5%
t-E2	173	82.0%	164	77.7%	163	77.2%	202	95.7%
Total sample	583	84.1%	541	78.1%	524	75.6%	662	95.5%
**Cognitive factor auditory attention/working memory**								
Placebo	222	84.7%	203	77.5%	196	74.8%	252	96.2%
o-CEE	188	85.5%	174	79.1%	165	75.0%	208	94.5%
t-E2	173	82.0%	164	77.7%	163	77.2%	202	95.7%
Total sample	583	84.1%	541	78.1%	524	75.6%	662	95.5%
**Cognitive factor visual attention/executive function**								
Placebo	222	84.7%	203	77.5%	196	74.8%	252	96.2%
o-CEE	187	85.0%	173	78.6%	164	74.5%	208	94.5%
t-E2	173	82.0%	164	77.7%	163	77.2%	202	95.7%
Total sample	582	84.0%	540	77.9%	523	75.5%	662	95.5%
**Cognitive factor speeded language/mental flexibility**								
Placebo	221	84.4%	201	76.7%	194	74.0%	252	96.2%
o-CEE	187	85.0%	172	78.2%	165	75.0%	208	94.5%
t-E2	173	82.0%	164	77.7%	162	76.8%	202	95.7%
Total sample	581	83.8%	537	77.5%	521	75.2%	662	95.5%
**POMS**								
Placebo	227	86.6%	205	78.2%	199	76.0%	252	96.2%
o-CEE	190	86.3%	180	81.8%	170	77.3%	209	95.0%
t-E2	174	82.5%	168	79.6%	166	78.7%	200	94.7%
Total sample	591	85.3%	553	79.8%	535	77.2%	661	95.4%

At baseline, the total KEEPS-Cog sample (*n* = 693), consisted of *n* = 262 women randomized to placebo, *n* = 220 women randomized to receive o-CEE + m-P, and *n* = 211 women randomized to receive t-E2 + m-P. Values in the table reflect percent of women providing data at each follow-up visit in relation to the number originally randomized to each treatment group.

### Cognitive Outcomes

Global cognitive performance over time, as measured with the 3MS score, did not differ between treatment groups. Similarly, models investigating MHT effects on the four cognitive factors were also statistically nonsignificant for each active treatment arm versus placebo. Table 3 includes the beta estimates and corresponding *p*-values for cognitive outcomes by treatment group.

**Table 3:** Treatment efficacy: beta estimates and confidence intervals for cognitive factor scores and affective scores across treatment duration.

Outcome Category	Test	o-CEE versus Placebo					t-E2 versus Placebo				
		Beta Estimate^a^	SE	*p*-Value	99% CI		Beta Estimate^a^	SE	*p*-Value	99% CI	
			
					Lower	Upper				Lower	Upper
**Cognitive outcomes**	**Cognitive factors**										
	Verbal learning/memory	−2.80 × 10^−3^	3.64 × 10^−3^	0.442	−9.94 × 10^−3^	4.34 × 10^−3^	−9.30 × 10^−4^	3.68 × 10^−3^	0.801	−8.14 × 10^−3^	6.29 × 10^−3^
	Auditory attention/working memory	9.53 × 10^−4^	1.70 × 10^−3^	0.575	−2.38 × 10^−3^	4.28 × 10^−3^	−8.10 × 10^−4^	1.72 × 10^−3^	0.639	−4.17 × 10^−3^	2.56 × 10^−3^
	Visual attention/executive function	3.73 × 10^−4^	1.93 × 10^−3^	0.846	−3.41 × 10^−3^	4.15 × 10^−3^	−4.70 × 10^−4^	1.95 × 10^−3^	0.808	−4.29 × 10^−3^	3.35 × 10^−3^
	Speeded language/mental flexibility	−3.40 × 10^−3^	2.59 × 10^−3^	0.189	−8.48 × 10^−3^	1.67 × 10^−3^	−1.15 × 10^−4^	2.62 × 10^−3^	0.661	−6.28 × 10^−3^	3.99 × 10^−3^
	**3MS examination**	1.02 × 10^−2^	7.46 × 10^−3^	0.178	−4.45 × 10^−3^	2.48 × 10^−2^	−9.40 × 10^−4^	7.52 × 10^−3^	0.840	−1.57 × 10^−2^	1.38 × 10^−2^
**Mood outcomes**	**POMS** ^**b**^										
	Tension-anxiety^c^	−3.01 × 10^−2^	1.06 × 10^−2^	0.004	−5.09 × 10^−2^	−9.34 × 10^−3^	−8.10 × 10^−3^	1.07 × 10^−2^	0.450	−2.91 × 10^−2^	1.29 × 10^−2^
	Depression-dejection^d^	−5.36 × 10^−2^	1.49 × 10^−2^	<0.001	−8.27 × 10^−2^	−2.44 × 10^−2^	−1.15 × 10^−2^	1.50 × 10^−2^	0.444	−4.10 × 10^−2^	1.80 × 10^−2^
	Anger-hostility^e^	−2.74 × 10^−2^	1.16 × 10^−2^	0.018	−5.02 × 10^−2^	−4.64 × 10^−3^	−1.42 × 10^−2^	1.18 × 10^−2^	0.228	−3.73 × 10^−2^	8.88 × 10^−3^
	Fatigue	−1.81 × 10^−2^	1.18 × 10^−2^	0.125	−4.11 × 10^−2^	5.01 × 10^−3^	−5.74 × 10^−3^	1.19 × 10^−2^	0.630	−2.91 × 10^−2^	1.76 × 10^−2^
	Vigor	3.01 × 10^−3^	1.18 × 10^−2^	0.799	−2.02 × 10^−2^	2.62 × 10^−2^	−1.81 × 10^−2^	1.20 × 10^−3^	0.125	−4.18 × 10^−2^	5.12 × 10^−3^
	Confusion-bewilderment	−4.58 × 10^−3^	7.92 × 10^−3^	0.563	−2.01 × 10^−2^	1.10 × 10^−2^	5.07 × 10^−3^	8.03 × 10^−3^	0.528	−1.07 × 10^−2^	2.08 × 10^−2^

Table reports the average rate of change over time. Study visits at which cognitive and mood data were collected occurred at baseline, 18 mo, 36 mo, and 48 mo after baseline. Beta estimates provided in the table describe the effect of treatment group (o-CEE versus placebo and t-E2 versus placebo) on the linear change over time for the cognitive factor scores, 3MS score, and the POMS subscales.All LME models controlled for age and education level. Models were estimated using restricted maximum likelihood and robust standard errors.

^a^Negative estimates suggest reduction in self-reported symptoms.

^b^65-item scale providing factors score for six mood states (subscales).

^c^An ES of 0.259 for the tension-anxiety subscale corresponds a small to medium ES.

^d^An ES of 0.486 for the depression-dejection subscale corresponds a medium ES.

^e^An ES of 0.218 for the anger-hostility subscale corresponds a small to medium ES.

SE, standard error.

### Mood Outcomes

Analysis of POMS scores revealed statistically significant findings (Table 3). Women treated with o-CEE demonstrated improvements over time in POMS tension-anxiety and depression-dejection scores compared to women receiving placebo. Based on a conservative alpha level of 0.01, the anger-hostility index *p*-value (*p* = 0.018) was statistically nonsignificant. As listed in Table 3, the beta estimate for the effect of treatment group (o-CEE versus placebo) on linear change over time on the POMS depression-dejection subscale was −5.36 × 10^−2^ (ES = 0.49, *p <* 0.001). That is, treatment was a significant predictor of linear improvements in POMS depression-dejection score. The beta estimate associated with o-CEE suggests a medium ES for the improvement in depression-dejection score relative to placebo. The ES for the statistically significant difference on the POMS tension-anxiety subscale with o-CEE was in the small to medium range: −3.01 × 10^−2^ (ES = 0.26, *p <* 0.001).

Standards for interpreting the clinical significance of POMS score changes were established for clinical trials examining pain control [46]. An expert panel recommended that a change of one standard error of the mean or half of a standard deviation should be considered clinically significant. When normative data are used as the standard, this corresponds to a two- to five-point change on the individual POMS subscales. S4 Text provides additional information, including a table listing the change in raw scores on the POMS subscales at each visit. According to these guidelines, the o-CEE group demonstrated clinically meaningful change at month 48 on the depression-dejection and tension-anxiety subscales and a borderline meaningful change on the anger-hostility subscale.

Compared to placebo, women assigned to receive t-E2 did not show improvement on any POMS subscales. Moreover, neither treatment group differed from placebo on the POMS confusion-bewilderment or fatigue subscales over time (*p*-values ranged from 0.125 to 0.630).

In a secondary analysis requested at peer review, we found no statistically significant effect of MHT on BDI-II score (S3 Table). Like other comparisons, the models examined performance between groups across treatment duration (i.e., treatment by time interaction).

### Prespecified Secondary Analyses

Four secondary analyses explored the influence of *APOE* ɛ4 carrier status, time since LMP, co-administration of m-P, and baseline depressive symptoms on response to treatment. The inclusion of *APOE* status (i.e., ɛ4 positive versus ɛ4 negative) did not influence cognitive or affective response to MHT, nor did time since LMP predict response to MHT for either mood or cognitive outcomes. Comparison of cognitive and mood outcomes obtained at visits occurring either during co-administration of estrogens and m-P (month 36) or during administration of estrogens alone (month 48) revealed no statistically significant differences, suggesting no overt cognitive or mood effects of m-P.

To explore whether baseline depression moderated the cognitive effects of MHT, baseline BDI-II score was entered into the model as a continuous variable, and its interaction with treatment evaluated. Sixty-four (10.0%) of the 637 women for whom both baseline BDI-II scores and cognitive factor scores were available reported symptoms suggestive of mild or moderately severe depression, although none were diagnosed with clinical depression; 52 women scored in the mildly depressed range (14 to 19 out of 63 points), and 12 women scored as having moderate depressive symptoms (20 to 28 out of 63 points). The results of this analysis were nonsignificant. For example, parameter estimates for women treated with o-CEE whose BDI-II scores suggested mild to moderately severe symptoms of depression at baseline, compared to women on placebo, were as follows: 5.65 × 10^−4^ for the auditory attention/working memory factor (*p* = 0.08) and 6.50 × 10^−4^ for the visual attention/executive function factor (*p* = 0.09). Of note, the range of mood symptoms as measured with the BDI-II was restricted in that women with scores indicative of severe depression were excluded from the study at screening. Findings from these planned secondary analyses were considered exploratory.

## Discussion

The KEEPS-Cog is, to our knowledge, the first large randomized clinical trial designed to examine cognition and mood effects for up to 4 y of two forms of MHT therapy in healthy, non-hysterectomized women in late menopausal transition and the early postmenopausal period (i.e., within 36 mo of LMP). Findings demonstrated that treatment with o-CEE resulted in statistically significant reduction in symptoms of tension-anxiety and depression-dejection, corresponding to medium and small to medium ESs, respectively. No mood effects were found for t-E2, and neither form of MHT affected cognition.

### Cognitive Effects of Menopausal Hormone Therapy

In this trial, neither MHT formulation affected cognitive function in healthy recently postmenopausal women. Contrary to our hypothesis, treatment with t-E2 did not improve cognition in recently postmenopausal women. The KEEPS-Cog findings are consistent with findings from the Women’s Health Initiative Memory Study of Younger Women (WHIMSY) [2] and a recent meta-analysis [47] suggesting that MHT use in the early postmenopausal period has no cognitive effects. The authors of the WHIMSY trial contrasted their findings to those from the WHIMS and others, which indicated adverse cognitive effects of both opposed and unopposed CEE in older postmenopausal women [12–17]. Together with WHIMSY, the findings from the KEEPS-Cog suggest that MHT is neither deleterious nor beneficial for cognition in younger women. Conflicting results of the WHIMS and others [12–17] and the WHIMSY [2] and KEEPS-Cog are likely due to study design differences discussed below.

### Oral Conjugated Equine Estrogens Improved Mood

The effects found in this trial are supported by previous studies [21] and results demonstrating that estrogens and progestins favorably influence neurotransmitters involved in mood regulation, including serotonergic and noradrenergic systems [48]. Similarly, our findings were highly consistent with results from a meta-analysis of clinical studies—the majority of which used o-CEE [49]—that found that MHT with or without progesterone exerts beneficial effects on mood during the menopausal transition and early postmenopausal periods, with a medium ES of 0.45.

In the Study of Women’s Health Across the Nation (SWAN), women experienced an increase in affective symptoms during menopause, and were two to four times more likely to experience depression during the menopausal transition compared to the premenopausal stage ([50,51]). Several studies report an increased incidence of affective symptoms during the menopausal transition [50–54]. Moreover, plasma E2 levels are lower among depressed women [55], suggesting that low estrogens may be associated with mood disorders. In contrast, women in the KEEPS-Cog who were in the late menopausal transition or early postmenopausal period and treated with o-CEE demonstrated improved depression and anxiety symptoms over women on placebo.

In comparison, studies involving CEE, with or without medroxyprogesterone (MPA), in women a decade or more past the menopausal transition [16,17] reported neutral effects of MHT on mood as assessed by general measures of mood [56]. The WHISCA findings together with those from the KEEPS-Cog suggest that CEE-mediated improvements in mood may occur only in recently postmenopausal women and women transitioning to postmenopause. Consequently, the effects of MHT on mood might be most relevant for symptoms occurring during or immediately after menopause [57,58]―a period when women exhibit a particular vulnerability for depression [50,51,59]. Still, it must be acknowledged that these potentially beneficial effects were found only in a subset of POMS outcomes, and ESs were in the medium and small to medium ranges. Moreover, mood effects were detected with the POMS and not the BDI-II. The two tests differ in that the POMS is sensitive to subclinical variations in multiple mood states, whereas the BDI-II assesses symptoms associated with major and minor depression. The BDI-II was included to monitor for major depressive episodes: an analysis of treatment effects on depression as measured with the BDI-II revealed no statistically significant differences between groups.

In contrast to the present findings for o-CEE and previous findings for t-E2 [7], therapy with t-E2 did not improve mood in the KEEPS-Cog. Although the potential mechanisms underlying these findings are currently unclear, and our findings offer only indirect comparisons, it is possible that differences in o-CEE and t-E2 formulations could account for the discrepancy. Results from the KEEPS confirm that women randomized to t-E2 and o-CEE demonstrate divergent estradiol and estrone profiles while on study medications [20]. Compared to placebo, women randomized to receive o-CEE demonstrated markedly increased levels of estrone, and small but statistically significant increases in estradiol levels at months 12, 36, and 48. In contrast, women receiving t-E2 exhibited statistically significant increases in estradiol, and small but statistically significant elevations in estrone levels at the same time points. Additionally, compared to estradiol, CEE is associated with higher levels of estrone sulfate, as well as many other metabolic products, the neurobiology of which are essentially unknown [60]. Only a direct comparison of the two formulations would address whether differential cognitive and mood efficacies exist. Indeed, other studies suggest that various CEE hormones exert differential degrees of neuroprotection [61,62] and that beneficial neuronal effects may be achieved by combining human and nonhuman hormones [61].

### Timing of Initiation of Hormone Therapy

Evidence from prospective cohort as well as laboratory studies supports the “critical window” hypothesis. Recent findings from the Cache County Study [1], a prospective cohort study of women starting unopposed MHT within 5 y of menopause, reported a reduced risk for Alzheimer disease compared to those initiating therapy beyond early menopause and compared to women who used opposed MHT. Similarly, results from several laboratory and animal studies that generated the “healthy cell bias” hypothesis [63–66] also support the critical window hypothesis by demonstrating that estrogens protected only non-diseased cells. Unlike the WHIMS and WHISCA [12–14,16,17], the KEEPS-Cog exclusively enrolled recently postmenopausal women, all of whom were cognitively and physically healthy.

### Forms and Administration of Progesterone

In the KEEPS-Cog, participants on active estrogens received cyclic m-P, a compound identical to endogenous progesterone, rather than a continuous MPA, as in the Women’s Health Initiative studies. Several studies point to adverse neurobiological and clinical effects of MPA on cognition and mood [67–70]. Moreover, while MPA is androgenic, there is evidence that m-P exerts multiple neuroprotective effects in the brain [70]. Supporting evidence from a small randomized, controlled trial suggested that CEE + m-P, but not CEE + MPA, improved cognition in recently postmenopausal women [71]. While the present study found no cognitive or mood effects of co-administration of estrogen and m-P compared to estrogen alone, the KEEPS-Cog design provided only a cursory examination of the potential effects of m-P.

### Limitations

The KEEPS-Cog results cannot be extrapolated to treatment extending beyond 4 y. This limitation is noteworthy because extended therapy is associated with elevated risks for adverse events, depending upon the formulation of MHT used and the health status of the woman at the time MHT is initiated. While some observational studies report prolonged beneficial effects of MHT initiated early in menopause [1,9–11], evaluation of women years after participation in MHT trials revealed a range of effects on cognitive and neuroimaging outcomes [2,72–74].

It is possible that women were aware of their treatment assignment due to reductions in menopausal symptoms. Indeed, between 80% and 90% of women assigned to the o-CEE and t-E2 groups accurately guessed that they were receiving MHT (see S2 Table); still, only women in the o-CEE group demonstrated improved mood, making it unlikely that the improvements in mood were related solely to reductions in menopausal symptoms or to inadvertent unblinding.

Several factors limit the study’s generalizability. Women in the KEEPS were predominantly white, generally well-educated, and at low risk for CVD. Thus, while KEEPS participants were not fully representative of the general postmenopausal US population, they may define a group of women for whom MHT would be appropriate. Additionally, hysterectomized women were excluded from the trial; thus, the findings may not apply to hysterectomized women.

Another limitation of the KEEPS-Cog is that it was neither large enough nor of sufficient duration to assess clinical events such as dementia; rather, the KEEPS-Cog was designed to detect changes in markers of cognitive and mood outcomes, arguably of less importance than incidence of clinical diagnoses. Finally, while a conservative *p*-value was selected to account for multiple comparisons, the possibility of type-1 error still exists.

### Conclusions

The results of the KEEPS-Cog demonstrate that MHT did not improve cognition when initiated in healthy recently postmenopausal women compared to placebo. For mood outcomes, administration of low-dose o-CEE for up to 4 y was associated with statistically significant improvements in symptoms of anxiety and depression, mood symptoms commonly seen in recently postmenopausal women; however, administration of t-E2 did not benefit mood. The statistically significant findings associated with o-CEE administration corresponded to small to medium ESs.

It is notable that neither MHT regimen altered cognition. In other words, results did not indicate adverse or beneficial cognitive effects associated with MHT. The divergent affective findings for o-CEE and t-E2 formulations are interesting, and perhaps expected, given the formulations’ differential chemical composition, mode of access to the systemic circulation, and binding affinity of the ligands for estrogen receptors. Importantly, the KEEPS-Cog finding that o-CEE and not t-E2 was associated with improved mood is contrary to our hypothesis. Additional studies directly comparing the long-term effects and mechanisms of action of these two formulations is warranted.

In total, we propose that the findings of the KEEPS and the KEEPS-Cog could be incorporated into clinical decisions for cardiovascularly healthy, non-hysterectomized women considering whether or not to use MHT. For example, a woman considering MHT for management of menopausal symptoms, including vasomotor, cognitive, and mood concerns, could be informed that MHT will not address her cognitive symptoms. Moreover, while various forms of MHT address vasomotor symptoms, not all MHTs demonstrate improvements in mood outcomes over placebo. She may also be reassured that time-limited MHT use around the age of menopause does not appear to have deleterious cognitive effects such as those observed in the older women enrolled in the WHIMS [12–14]. Finally, discussions of the risks associated with MHT, for example, the risk for breast cancer associated with extended therapy, must be considered alongside the benefits for mood and vasomotor symptoms. While limited, preliminary data suggest that use of low-dose MHTs for a brief period of time may be associated with a lower risk for breast [75] and endometrial [76] cancers. The effects of low-dose and brief MHT on CVD risk factors are mixed [77,78] compared to traditional doses of MHT. Overall, the KEEPS [20] may provide some of the most comprehensive safety data for two low-dose MHTs used for brief duration. Further examination is needed, especially considering the variations in dose, hormone formulation, and route of administration used in studies examining the safety profile of low-dose estrogens.

Together with her health care provider, a woman experiencing problematic symptoms around her menopausal transition can weigh the known risks of MHT against potential benefits for her unique symptom profile, and make an informed decision as to whether she would benefit from MHT, or whether she would prefer to manage symptoms through other means. Overall, the KEEPS-Cog findings provide valuable information to women considering the various options for managing menopausal symptoms.

## Supporting Information

S1 Table Among women completing the symptom scale, the number and percent of women reporting moderate to severe hot flashes, by treatment group and visit. (PDF)

S2 Table Assessment of participant blinding for women in the KEEPS-Cog. Women were asked whether they thought they were on active MHT or placebo at the end of the study.
(PDF)

S3 Table Treatment efficacy: beta estimates for BDI-II scores across treatment duration. (PDF)

S1 Text KEEPS trial protocol. (PDF)

S2 Text CONSORT statement. (DOC)

S3 Text Supplementary information about LME models. (PDF)

S4 Text Clinical relevance of statistically significant findings. (PDF)

S5 Text IRB approval letter. (PDF)

## Abbreviations:

3MS: Modified Mini-Mental State
BDI-II: Beck Depression Inventory, 2nd edition
BMI: body mass index
CEE: conjugated equine estrogens
CVD: cardiovascular disease
ES: effect size
IRB: institutional review board
KEEPS: Kronos Early Estrogen Prevention Study
KEEPS-Cog: Kronos Early Estrogen Prevention Study–Cognitive and Affective Study
LME: linear mixed-effects
m-P: micronized progesterone
LMP: last menstrual period
MHT: menopausal hormone therapy
MMSE: Mini-Mental State Examination
MPA: medroxyprogesterone
NIH: US National Institutes of Health
o-CEE: oral conjugated equine estrogens
POMS: Profile of Mood States
SD: standard deviation
t-E2: transdermal estradiol
WHIMS: Women’s Health Initiative Memory Study
WHIMSY: Women’s Health Initiative Memory Study of Younger Women; WHISCA, Women’s Health Initiative Study of Cognitive Aging
WMS-3: Wechsler Memory Scale, 3rd edition
