# Supplementary material for: Effects of Hormone Therapy on Cognition and Mood in Recently Postmenopausal Women: Findings from the Randomized, Controlled KEEPS–Cognitive and Affective Study
Source: PLoS Med. 2015 Jun 2;12(6):e1001833. doi: 10.1371/journal.pmed.1001833 (PMC4452757; doi:10.1371/journal.pmed.1001833)
Supplement: S1 Table — (PDF) [file pmed.1001833.s001.pdf]

S1 Table. Among Women Completing the Symptom Scale, the Number and Percent of Women Reporting Moderate to Severe Hot Flashes, by Treatment Group and Visit.

	Placebo<br>N = 262	o-CEE<br>N = 220	t-E2<br>N = 211
Hot flashes, N (% of randomization group) reporting moderate to severe hot flashes			
At Baseline, N=693	120/262<br>(45.8%)	96/220<br>(43.6%)	88/211<br>(41.7%)
At 6 months,* N=589	64/224<br>(28.6 %)	8/185<br>(4.3%)	12/180<br>(6.7%)
At 12 months,* N=590	46/222<br>(20.7%)	7/190<br>(8.4%)	15/178<br>(3.7%)

*p-value <0.01
