# Supplementary material for: Effects of Hormone Therapy on Cognition and Mood in Recently Postmenopausal Women: Findings from the Randomized, Controlled KEEPS–Cognitive and Affective Study
Source: PLoS Med. 2015 Jun 2;12(6):e1001833. doi: 10.1371/journal.pmed.1001833 (PMC4452757; doi:10.1371/journal.pmed.1001833)
Supplement: S2 Table — Women were asked whether they thought they were on active MHT or placebo at the end of the study. (PDF) [file pmed.1001833.s002.pdf]

S2 Table. Assessment of Participant Blinding for Women in KEEPS-Cog. Women were asked whether they thought they were on active MHT or placebo at the end of the study.

	Placebo N = 208	o-CEE N = 167	t-E2 N = 161
Unblinding: Women were asked whether they thought they were on active MHT or placebo at the end of the study, N=536			
Number (%) guessed MHT assignment	72 (34.6%)	144 (89.4%)	133 (80.2%)
Number (%) guessed Placebo assignment	136 (65.4%)	33 (19.8%)	17 (10.6%)
