# Supplementary material for: Effects of Hormone Therapy on Cognition and Mood in Recently Postmenopausal Women: Findings from the Randomized, Controlled KEEPS–Cognitive and Affective Study
Source: PLoS Med. 2015 Jun 2;12(6):e1001833. doi: 10.1371/journal.pmed.1001833 (PMC4452757; doi:10.1371/journal.pmed.1001833)
Supplement: S3 Table — (PDF) [file pmed.1001833.s003.pdf]

S3 Table. Treatment efficacy: beta estimates BDI-II scores across treatment duration.

	Treatment Groups Compared to Placebo[b]						
	o-CEE vs Placebo				***t-E2 vs Placebo***		
	Beta Estimate[d]	***SE***	***p-value***		***Beta Estimate[d]***	***SE***	***p-value***
BDI-II	$-8.66x10^{-3}$	$1.11x10^{-2}$	0.436		$-3.13x10^{-3}$	$1.11x10^{-2}$	0.782
