# Supplementary material for: Effects of Hormone Therapy on Cognition and Mood in Recently Postmenopausal Women: Findings from the Randomized, Controlled KEEPS–Cognitive and Affective Study
Source: PLoS Med. 2015 Jun 2;12(6):e1001833. doi: 10.1371/journal.pmed.1001833 (PMC4452757; doi:10.1371/journal.pmed.1001833)
Supplement: S1 Text — (PDF) [file pmed.1001833.s004.pdf]

Protocol Number: KLRI-04-1, Version 5.7 March 14, 2007

WIRB Protocol #20040792

The Kronos Early Estrogen Prevention Study (KEEPS)

Title: Effects of estrogen replacement on atherosclerosis progression in recently menopausal women

Sponsor: Kronos Longevity Research Institute
2222 East Highland, Suite 220
Phoenix, AZ 85016

Principal Investigator: S. Mitchell Harman, M.D., Ph.D.

TABLE OF CONTENTS

GLOSSARY 3
HYPOTHESES: 4
SIGNIFICANCE AND BACKGROUND: 4
1. Significance 4
2. Background 5
RESEARCH OBJECTIVES: 12
EXPERIMENTAL DESIGN: 13
SPECIFIC METHODOLOGIES: 14
1. Human Subjects 14
2. Study medications 17
3. Study Procedures 18
4. Outcome Measures 22
5. Statistical Analyses and Sample Size Estimation 34
6. Safety Monitoring and Procedures for Protecting Against Risks 38
POTENTIAL PITFALLS 50
FACILITIES AVAILABLE 52
CONFIDENTIALITY 53
COMPENSATION and CHARGES 53
REFERENCES 54

GLOSSARY

CEE ------- Conjugated equine estrogens, mixed estrogens, mainly sulfate salts, derived from pregnant mare urine

CRP ------- C reactive protein, a markers and probable mediator of inflammation in the arterial wall

CVD------ Coronary vascular disease

DEXA ---- Dual X-ray absorptiometry for bone density and body composition

EBT ------- Electron beam tomography (for measuring coronary calcium burden)

ERT --------Estrogen replacement therapy taking of an effective estrogen without a progestin

HERS ----- The Heart and Estrogen/progestin Replacement Study

HRT-------- Hormone replacement therapy: taking of aneffective estrogen with a progestin either continuously or intermittently

CIMT-------- Intimal medial thickness of common carotid artery

MDCT ---- Multidetector X-ray computerized tomography (coronary calcium)

KEEPS ---- The Kronos Early Estrogen Prevention Study

MHT -------Menopausal hormone treatment taking of an effective estrogen with or without a progestin

MPA ------- Medroxyprogesterone acetate, a commonly used synthetic progestin

NCEP ------ National Cholesterol Education Program

PEPI ------- The Postmenopausal Estrogen/Progestin Interventions trial

QOL ------- Quality of life

WHI -------- Women’s Health Inititative

HYPOTHESES:

1. Menopausal female hormone treatment (MHT) initiated at, or shortly after, the menopause will prevent or retard progression of atherosclerosis.
2. Reduction in rate of atherosclerosis progression is related to effects of MHT on measurable risk factors for atherosclerosis.
3. Transdermal delivery of 17β-estradiol (E_2) provides:
   a. protection against atherosclerosis similar to oral conjugated equine estrogens (CEE).
   b. differential effects on risk factors for atherosclerosis and thromboembolic disease compared with oral CEE.

SIGNIFICANCE AND BACKGROUND:

1. Significance –

Because the chronic diseases potentially affected by menopausal hormone therapy MHT (heart disease, breast cancer, stroke, osteoporosis) are among the most common killers and cripplers of women, with many billions of dollars of health care costs per year at issue, obtaining accurate information as to the risk/benefit ratio of MHT in various target groups is of great importance. Several recent randomized controlled trials have reversed the long-standing conclusion, based on many years of observational studies, that MHT reduces heart disease incidence by approximately 50%. However, studies leading to this reversal were conducted in women who either had existing clinical heart disease, or who were, on average, many years older, and many years further from the menopause than women in the prior observational studies, or women who typically initiate MHT. Heart disease is far and away the greatest single killer of women, accounting for 45% of total mortality (vs. about 5% for breast cancer). Osteoporotic bone fractures, which MHT has been shown to prevent, account for significant additional morbidity and mortality. If the conclusion that MHT is not cardioprotective is inapplicable to newly menopausal women, many millions of women may endure cardiac events and bone fractures that could have been prevented over the next 30 years, as the "baby-boom" generation transits old age. Therefore, we believe that it is vital that this issue be further explored.

2. Background

Before 1998, the majority of studies supported the conclusion that the balance between risks and benefits of long-term MHT, given as estrogen (ERT) or combined estrogen/progestin hormone (HRT) replacement therapy, was favorable for most women [1, 2]. Epidemiologic data, derived from large, carefully analyzed cohort and retrospective studies, demonstrated that, while long-term MHT was associated with a small increase in breast cancer risk [3, 4], in most [1, 5-7], but not all [8, 9], studies there appeared to be high degrees of protection (30-50% reductions) against coronary heart disease, as well as all-cause mortality [10-14] and osteoporotic fractures [15-17]. Favorable estimates of net risk/benefit owed largely to the fact that atherosclerotic heart disease is approximately five times more likely to kill women over age 60 than is breast cancer and that osteoporotic hip fractures contribute about as much to morbidity and mortality as does breast cancer in women over 70 [6].

Interpretation of epidemiological and observational studies has been confounded by the fact that women choosing to take MHT tend to be better educated and have higher income levels and better general health habits than non-users, factors associated with *a priori* reductions in risk of coronary events [14]. Various attempts to match subpopulations or control statistically for these confounders usually showed persistent cardiovascular protection by HRT [5, 12, 14, 18]. However, no MHT-related protection against coronary vascular disease (CVD) was found after correction for socioeconomic factors in a recent large meta-analysis of previous population studies [19]. Comparisons of age-matched women with continuing menstrual cycles vs. an early natural menopause, where choice of using or not using estrogen was not an issue, revealed an earlier occurrence of coronary disease in the estrogen-deficient women [20, 21].

The biological plausibility of cardioprotection by MHT is supported by a body of basic investigations demonstrating that estrogens improve a variety of risk factors for atherosclerosis. A recent large prospective trial, the Postmenopausal Estrogen/Progestin Interventions (PEPI) trial, which compared CEE with 3 different CEE-progestin combinations and placebo in 875 healthy postmenopausal women aged 45 to 64 years, showed increases in HDL and decreases in LDL cholesterol and fibrinogen in women receiving active estrogen regimens [22]. Many other studies have shown favorable lipid

effects, including lowering of LDL-C and Lp(a) and raising of HDL-C levels [23, 24]. Oral, but not transdermal, estrogen has also been shown to decrease plasma levels of homocysteine [25], a non-lipid risk factor for atherosclerosis. Favorable effects on arterial wall function include improvement of arterial compliance [26-28] and blood pressure lowering [29-31]. Potential beneficial effects on inflammatory factors, include reduced endothelial expression of adhesion factors such as e-selectin, ICAM-1 and VCAM-1 plus increased Fas ligand [32-34]. Estrogens also appear to act as antioxidants, with potential, but not proven, benefits for reducing LDL oxidation and the oxidative component of arterial wall inflammatory processes [35-38].

Before 1998, there were no controlled, randomized trials of sufficient power for rates of clinical events to confirm or refute putative cardioprotective benefits observed in the epidemiological studies [39]. The only early prospective trial with clinical endpoints was a small study showing no difference in cardiac event rates in 84 women randomized to estrogen + progestin or placebo after 10 years [40]. Other randomized, prospective studies of MHT employed surrogate endpoints. In one such study of 86 women, carotid intimal medial thickness (CIMT) increased in the placebo group and regressed among MHT users, which difference appeared to be independent of lipoprotein concentrations [41]. In a trial of estrogen in the prevention of atherosclerosis (EPAT), among 77 women who received unopposed estrogen and no lipid-lowering drugs, the average rate of progression of CIMT was significantly lower in the E_2- than in placebo-treated group [42]. A recent study in 2,213 postmenopausal women of whom 1,172 (53%) were current users of MHT has shown that current MHT users were significantly more likely to have a coronary artery calcium score <100 and less likely to have a score >400 than non-MHT users, after adjustment for cardiac risk factors [43], suggesting that estrogen use is associated with less progression to complex atheromatous lesions.

The concept of estrogen cardioprotection was called into question in 1998 by publication of the Heart and Estrogen/progestin Replacement Study (HERS) [44], a randomized controlled trial of secondary prevention, showing that women with known CVD given MHT had slightly worse cardiac outcomes than those on placebo after 4 years. Consistent with the above findings were CIMT measurements from a subset of HERS patients [45] demonstrating no significant difference between the rates of progression of arterial wall

thickening in the MHT-treated and placebo groups (26 vs. 31 μm/year; P=0.44) and findings from another study, employing serial quantitative coronary angiography, which showed that coronary narrowing progressed at equal rates in estrogen- and placebo-treated women [46]. However, because both these trials were done in women with prevalent CVD neither addressed the issue of primary prevention.

The Women's Health Initiative (WHI) hormone replacement study E+P arm [47] was a randomized, controlled, blinded trial in approximately 16,000 women, comparing a marketed MHT combination tablet (PremPro®, Wyeth; 0.625 mg CEE and 2.5 mg medroxyprogesterone acetate) with placebo. Subjects were postmenopausal women ages 50-79 (mean: 62.7) generally without clinical CVD. Women experiencing vasomotor instability symptoms of estrogen deficiency were discouraged from joining the study. There was an excess of coronary events in year 1 and an increase of borderline statistical significance in the rate of coronary events per 10,000 women/year of CHD (37 vs. 30) in the E+P vs. the placebo group over 5.2 years. The E+P group also had more breast cancer (38 vs. 30) as well as more strokes (29 vs. 21), and thromboembolic disease (34 vs. 16), but no difference in numbers of deaths (52 vs. 53). Beneficial effects were reductions in the rates of colon cancer (45 vs. 67), and bone fractures (147 vs. 191). The investigators in the WHI study concluded that combined estrogen-progestin MHT was not beneficial overall in postmenopausal women, based on the observed excess of breast cancer and the failure to protect against CVD.

The inconsistency between results of this WHI study, and those in prior observational studies, requires explanation. As pointed out by Lemay et al. [48] the older age distribution and late start of MHT in the WHI study does not correspond to the traditional use of MHT in the earlier studies. Women in the observational studies generally started MHT in the perimenopausal phase (ages 45-55) for symptoms of estrogen deficiency (such as hot flashes, insomnia, mood swings and dyspareunia), whereas the vast majority of women in the WHI study had been postmenopausal without estrogen treatment for many years before randomization to MHT or placebo. Thus, if plausible evidence supports the concept that starting MHT "late," after a significant period of estrogen deprivation, is likely to have lesser or even opposite effects on atherosclerosis, compared with MHT initiated in the

perimenopausal period, then the older age of the WHI population might account for the different effects observed.

One possible mechanism leading to different cardiovascular outcomes between early- and late-start MHT is the increase in tendency of blood to clot produced by oral estrogens absorbed into the hepatic-portal circulation during "first-pass" through the liver. Oral estrogen has been shown to increase hepatic production of clotting factors, decrease anti-clotting factors, and result in greater production of fibrin split products, consistent with accelerated intravascular thrombus formation, effects not observed with estrogen delivered directly into the systemic circulation by the transdermal route [49-51]. In women with pre-existing complex "at risk" atherosclerotic lesions, increased clotting tendency could predispose to thrombus formation and propagation, hence a greater incidence of cardiovascular events. This mechanism could also have contributed to the excess of strokes and thromboembolic disease observed in the HRT group.

Additional data, indirectly supporting this hypothesis, comes from an analysis of the Nurses Health Study data published in 2000 [52]. As shown in Table 1, the effects of estrogen use appeared to be dose-dependent with similar approximately 40% reductions in risk in women taking 0.3 or 0.625 mg/day, but less protection in women on higher doses of 1.25 mg/day or more, perhaps because at high doses the effects of oral estrogen on clotting begin to supersede effects on atherosclerosis development, even in women who initiated MHT early.

Table 1: Effects of Conjugated Estrogen Use on Cardiovascular Event Risk in the Nurses Health Study [52]

CEE Use	Women	Cases	Adjusted Risk	(95% C.I.)
Never	313,661	609	1.0	- -
0.3 mg/day	19,964	19	0.58	(0.37 – 0.92)
0.625 mg/day	116,150	99	0.54	(0.44 – 0.67)
1.25+ mg/day	39,026	41	0.70	(0.51 – 0.97)

There is also evidence suggesting that the effects of ERT and HRT may be divergent depending on the stage of the atherosclerotic lesions. Potentially negative effects of estrogen on atherosclerosis include increases in C reactive protein (CRP) [53-55] and increased activity of matrix metalloproteinases (MMP2 and MMP9) [56, 57]. CRP has been implicated as an independent risk factor for clinical cardiac events [58, 59], probably by contributing to

the inflammatory processes that convert "fatty streak" stage plaques into complex lesions (foam cells, necrosis, calcification, etc.) [60]. CRP is not closely associated with other known risk factors for prevalent atherosclerosis, suggesting that elevated CRP may be a stronger marker of event risk than of early plaque development [61]. Local activation of metalloproteinases have been implicated as a proximate cause of rupture of the fibrous cap of late-stage atherosclerotic plaques and estrogens increase metalloproteinase activity [62, 63]. Plaque rupture induces thrombus formation, which, when extensive enough to occlude arterial blood flow, produces an acute coronary event. Thus, in women with established complex atherosclerotic lesions, estrogen-induced increases in tendency for plaque rupture and thrombosis might be expected to cause a greater incidence of clinical CHD events. Such an increase was seen in the first year of MHT treatment both in the HERS [44] and WHI [47] trials.

Direct evidence indicates that in surgically postmenopausal cynomolgus monkeys athero-protective effects of estrogen are limited to the early stages of atherogenesis. These primates, which develop atheromatous lesions indistinguishable from those in humans when fed an atherogenic diet, have consistently shown estrogen replacement with CEE to reduce coronary atherosclerosis by as much as 50-70% if treatment is begun immediately after ovariectomy [64-66]. However, no beneficial effect is seen when CEE treatment is delayed for 2 years [67], leading the investigators to conclude that, in the delayed treatment model, "Hormone replacement therapy did not enhance regression of established coronary atherosclerosis." These findings are entirely consistent with the above-noted failure to observe secondary prevention by MHT in human trials [44-46].

The same mechanisms affecting coagulation and inflammation, which we hypothesize may have contributed to the higher rates of cardiovascular events, could also have been responsible for the higher rates of stroke and loss of cognitive function observed in the WHI study. A more complete analysis of the stroke endpoint [68] revealed that 79.8% of strokes were ischemic. The adjusted hazard ratio (HR) for MHT vs. placebo was significant for ischemic (HR=1.44; 95% CI, 1.09-1.90) but not for hemorrhagic or combined strokes, suggesting an etiologic role for hypercoagulability. However, higher levels of inflammation-associated factors (C-reactive protein, IL-6, e-selectin) appeared to be more predictive of stroke risk than those related to clotting (fibrinogen, Factor VIII). Whether more

sophisticated determinations of coagulation factors, or changes therein, might have been more indicative is a matter for speculation. As with CVD, data from the Nurses Health Study [52] may shed some light on this issue. As shown in Table 2, the risk of ischemic stroke increases with increasing doses of CEE, with a 57%, (nonsignificant) reduction in risk at the 0.3 mg dose and progressively increased risks at higher doses.

Table 2: Effects of Conjugated Estrogen Use on Ischemic Stroke Risk in the Nurses Health Study [52]

CEE Use	Women	Cases	Adjusted Risk	(95% C.I.)
Never	313,661	160	1.0	- -
0.3 mg/day	19,964	4	0.43	(0.16 – 1.16)
0.625 mg/day	116,150	73	1.44	(1.07 – 1.93)
1.25+ mg/day	39,026	29	2.00	(1.32 – 3.05)

In the WHI E+P study, there was also an increase in risk of new-onset dementia in the MHT group (HR=2.05; 95% CI, 1.21-3.48), equivalent to 23 excess cases of dementia per 10,000 women per year [69]. Approximately 80% of cases were classified as Alzheimer's disease (AD) in both study groups. However, as the authors pointed out, in living patients there is considerable overlap and ambiguity between multi-infarct dementia and AD [70], and infarcts due to small vessel occlusion are believed to contribute to AD pathogenesis [71]. Mild cognitive impairment defined by MiniMental examinations did not differ between treatment groups, but MHT did not improve cognitive function, and there was a tendency for more women in the MHT group to have large decreases in MiniMental scores [72]. Taken together, the brain-related findings in the WHI study are surprising in that, parallel to the situation with CVD, most prior epidemiological [73-75] and prospective observational studies [76, 77], as well as two meta-analyses [78, 79], have suggested that menopausal women who take estrogen show reduced risk of AD dementia. Moreover, there are several well-described biological mechanisms by which estrogens appear to enhance neurological function and exert neuroprotective effects [80]. However, estrogens do not appear to improve or slow progression of established AD [81, 82] and may need to be administered at the menopausal transition to be significantly preventive [83, 84]. In addition, progestins have been shown to antagonize estrogen's beneficial effects on the CNS in both animal [85] and human [75] studies. To summarize, it seems likely that the failure to demonstrate neuroprotection by MHT and the increases in stroke and dementia risks observed in the WHI

study were also a consequence of studying older women, the great majority of whom were many years postmenopausal. It is possible that the greater risk of dementia in the MHT group was a consequence of multiple small infarcts due to the same changes in coagulation and inflammation factors that produced the observed increases in ischemic stroke and thrombosis-related disease in general. The findings of a higher risk of stroke [86] and the greater incidences of dementia and decreased cognitive function in the old (but not the young) E-only treated WHI women [87, 88] are also consistent with the hypothesis that oral estrogen generates both macro- and micro-cerebrovascular thrombosis in older women.

Thus, if the older women studied in the WHI E+P trial had significantly greater prevalence of advanced (but asymptomatic) atherosclerosis, actions of MHT on clotting, inflammation, and local plaque enzyme activities could well have interacted to increase cardiac events (and strokes). Two additional bits of evidence support this supposition. Cardiac event rates are very low in cycling women, but increase exponentially after the menopause [89]. Second, in a large series of women with no symptoms of heart disease, coronary artery calcification measured by EBT, an indicator of advanced atherosclerotic lesions, is practically absent in women up to menopausal age, but increases rapidly after age 54 [90]. These data are consistent with the concept that before menopause very few women have significant numbers of advanced plaques, but that the number of "at risk" plaques increases substantially within a few years of ovarian failure.

The most compelling data suggesting that elapsed time post-menopause is a critical determinant of estrogen effect on cardiovascular risk comes from the WHI study itself. In the definitive report on cardiovascular outcomes by Manson, *et al.* [91] there was no effect of age *per se* on risk ratio for cardiac events. However, risk ratios (HRT vs. placebo group) of 0.89, 1.22, and 1.71 were calculated for women randomized at menopausal durations of, respectively, <10 years, 10 - 19 years, and ≥20 years. Although this apparent trend was non-significant, it is of note that the women with the shortest menopausal duration had a risk ratio less than 1.0, a finding consistent with the contention that time of initiation of estrogen is critical.

More recently the estrogen only (E-only) arm of the WHI hormone trial was discontinued because there was an excess of strokes in the absence of evidence of reduced risk of heart disease [86]. However, in contrast to the E+P arm, in the E-only arm of the study, the

previously observed trend for an excess of heart disease in the first year was not seen, nor was there an excess of breast cancer. Moreover, younger women (ages 50-59) randomized to CEE in the estrogen-alone study had no increase in stroke risk and an approximately 50% reduction in cardiac events (which was non-significant; RR= 0.56, 95% CI 0.30-1.03) compared with those randomized to placebo [86]. A similar trend for decrease in CHD was also seen in women of all ages in years 6 to 8. Thus, the weight of available evidence leads us to conclude that further trials of MHT in a target population younger than that studied in the WHI study will be required to elucidate the risk/benefit ratio of estrogen replacement initiated early. As detailed below, we expect very low rates of serious adverse events in women younger than 58 years during five years of study. Thus, we believe the proposed "Kronos Early Estrogen Intervention Study (KEEPS) will be both useful and ethical.

We estimate that a randomized prospective trial with clinical cardiovascular and other endpoints would require a minimum of 9,000 women per study group and 7 to 10 years to complete. Given the evident value of comparing the effects of oral vs. systemically administered estrogen, such a study would require a minimum of three study groups, hence approximately 27,000 women completing the protocol. Before any such gargantuan effort can be recommended, we suggest that it is advisable to obtain more and better data as to whether early-initiated MHT inhibits progression of atherosclerosis and/or prevents development of complex atheromata, as determined by modern quantitative imaging techniques. Such a study should also investigate whether baseline values for, or changes in, known atherosclerosis risk factors predict arterial response to MHT in order to better identify candidates more (and less) likely to benefit from MHT in future studies. To this end, we propose to conduct the KEEPS, as outlined below.

RESEARCH OBJECTIVES:

1. Demonstrate in a randomized, placebo-controlled clinical trial whether 4 years of MHT with estrogen initiated at, or shortly after, the menopause retards:
    a. progression of carotid intimal/medial thickness (CIMT), as determined by B-mode ultrasound.

   b. development of complex atherosclerotic lesions in the coronary arteries as indicated by measurements of vascular calcium with computerized X-ray tomography.
2. Compare effects of MHT using oral CEE with those of transdermal 17β-E_2 on:
   a. lipid risk factors for atherosclerosis including: total LDL and HDL cholesterol; LDL subfractions, triglycerides; Lp(a);
   b. inflammatory markers including homocysteine; C-reactive protein, interleukin-6 and prothrombin activator inhibitor 1 (PAI-1)..
   c. risk factors for thromboembolic disease and markers of blood hypercoagulability including activated factor XII, tissue factor, D-dimer, soluble CD-40, antithrombin –III (AT-III), and tissue plasminogen activator (TPA)
3. Investigate the extent to which effects of MHT on CIMT and coronary calcium progression are predicted by baseline levels of, or changes during treatment in, the above-enumerated atherosclerosis risk factors.
4. Examine effects of MHT vs. placebo on
   a. body composition by dual X-ray absorptiometry
   b. bone density by dual X-ray absorptiometry
   c. cognitive, affect, and quality of life measures
5. Compare adverse effect profiles of oral CEE with those of transdermal 17β-E_2, examining incidences of:
   a. cancers, especially breast and endometrial cancer
   b. thromboembolic disease, including thrombophlebitis, pulmonary embolus, and stroke
   c. symptoms or new diagnoses of gallbladder disease
   d. vaginal bleeding, nausea, edema and headache

EXPERIMENTAL DESIGN:

The proposed study will be a randomized, placebo-controlled double-blinded, prospective trial with two active treatment groups and one placebo group. It will be a multi-center trial with 8 centers, at which subjects will be entered and followed, and a separate coordinating center, which will oversee and administer the study. Duration of the study is proposed for 4 years after

randomization for each study subject, with assessment of the primary endpoint, CIMT, twice at baseline, once at 12, 24, and 36, and twice at 48 months or on exit from the study, whichever comes first and, as a secondary endpoint, coronary calcium by EBT at baseline and 48 months. Other secondary endpoints include lipid profiles, Lp(a), clotting factors and inflammatory markers at 12, 36, and 48 months, DEXA measurements of body composition and bone density at baseline, 12, 24, 36, and 48 months. and measures of cognitive function, affect, and quality of life and at baseline, 18, 36, and 48 months. Laboratory determinations for safety considerations (serum chemistries, CBC, U/A) will be carried out at screening and 48 months. Imaging procedures for safety considerations will be performed (mammogram at baseline, 12, 24, 36, and 48 months; endometrial thickness by ultrasound at baseline). Data will be evaluated and analyzed only at the end of the study.

SPECIFIC METHODOLOGIES:

1. Human Subjects
   a. Study Subjects- Subjects will be healthy female volunteers recruited from the local regions surrounding each participating study center. A total of 720 women will be randomized to placebo or one of two active treatments in the ratio of 17:14:14 or 272 to placebo and 224 to each of the active treatment groups. We expect that we will lose 4% per year of follow-up. Thus, at each center 34 women will be randomized to placebo, 28 to oral CEE and 28 to transdermal E_2. Women will be 42-58 years of age, will have had cessation of menses at or after age 40 and no menses for a minimum of 6 and a maximum of 36 months at screening. Subjects may or may not have current vasomotor estrogen deficiency symptoms, will not have taken estrogen- or progestin-containing medication (oral contraceptive or hormone replacement) within 3 months of randomization, and will have plasma FSH levels measured at $\geq$35 ng/ml and plasma E_2 levels of <40 pg/ml.
   b. Recruitment - Subjects will be recruited from local ambulatory, home-dwelling populations using methods outlined below under "Study Procedures." Initial contact will be by phone call from the candidates to a trained study screener who will collect basic information determining eligibility for study and arrange a first (screening) appointment.

c. Consent- The study protocol and informed consent document will be reviewed and approved by a national IRB for the coordinating center and the local IRB at each study center before recruiting begins. Subjects will be mailed a copy of the study consent form at least 5 days before the scheduled screening visit. At the screening visit a study investigator or other trained clinical study center professional will meet privately with each individual subject, explain the study, solicit and answer any questions, and test each subject as to her understanding of the purpose, requirements, and risks of the study (using a standard printed question set) before requesting signature on the consent document.

d. Inclusion Criteria:

- 42-58 years of age at date of randomization
- menses absent for at least 6 months and no more than 36 months
- last spontaneous menses occurring after age 40
- good general health
- plasma FSH level ≥ 35 mIU/ml (μu/L) and E_2 levels < 40 pg/ml or one of these two hormone criteria plus absence of menses for at least one year. (FSH and E_2; FSH and E_2 assays may be repeated once if out of range on initial evaluation).
- normal mammogram within 1 year of randomization

e. Exclusion Criteria:

- use of estrogen- or progestin-containing medication or phytoestrogen containing supplements (e.g. soy concentrates or extracts) within 3 months of randomization; soy containing foods (e.g. tofu, soy milk) will be permissible
- Use of selective estrogen receptor modulators (SERMs) such as Raloxifene, Tamoxifen, etc.
- Self reported, known BrCa positive genotype (KEEPS will not screen or advise screening for BrCa genes)
- endometrial thickness >5 mm by vaginal ultrasound, unless complex endometrial hyperplasia with or without atypia and endometrial cancer are excluded by biopsy
- *in utero* exposure to diethylstilbestrol (DES) (maternal treatment) by self-report
- current smoking- more than 10 cigarettes/day by self report

- obesity- body mass index (weight in kg/height in meters^2) > 35
- history of clinical CVD including myocardial infarction, angina, or congestive heart failure
- history of cerebrovascular disease including stroke or transient ischemic attack (TIA)
- history of thromboembolic disease (deep vein thrombosis or pulmonary embolus)
- known carrier of Factor V Leiden, prothrombin G20210A or other prothrombotic allele
- coronary calcium score ≥ 50 units
- history of untreated (no cholecystectomy) gallbladder disease
- dyslipidemia – LDL cholesterol >190 mg/dl, or current NCEP criteria for statin treatment based on Framingham Risk Score, if personal physician prescribes and patient initiates lipid-lowering medication
- hypertriglyceridemia - triglycerides >400 mg/dl
- medications – current or recent (3 months) use of lipid lowering medications or supplements (e.g. statin, fibrate, > 500 mg/day of niacin, red rice yeast)
- nut allergy (Prometrium® includes peanut oil)
- uncontrolled hypertension – systolic BP >150 and/or diastolic BP > 95
- hysterectomy
- history of, or prevalent, chronic diseases including any cancer (other than basal cell skin cancers), renal failure, cirrhosis, uncontrolled hypertension, diabetes mellitus, and endocrinopathies other than adequately treated thyroid disease
- known HIV infection and/or medications for HIV infection
- Active severe clinical depression (BDS score > 17)
- Dementia (MMSE score < 23)
- Results of any safety laboratory test (chemistries, TSH, CBC, U/A) more than 20% above or below limits of normal for center laboratory at which value is measured, unless cleared by either a repeat value within acceptable limits or further medical screening by a qualified medical provider documenting absence of

any other evidence of pathology predicted by the out-of-range laboratory value in question.

2. Study medications –

Study medications will be shipped in bulk to the designated research pharmacy (to be named). Shipments will be labeled by the manufacturer as to batch, dates of production and expiration, and whether drug is active or placebo. A registered, licensed pharmacist will supervise receipt, storage, dispensing, and shipping of study medications. Medications will be dispensed as packages containing 3 month supplies, consisting of 93 conjugated estrogen (CEE) tablets, 15 estradiol (E_2) skin patches, and 36 progesterone capsules. Dispensing and labeling will be according to study subject number based on charts showing the randomization scheme for each center. There will be 3 types of packages:

	Tablet	Patch	Capsule
1.	Active CEE	Placebo	Active progesterone
2.	Placebo	Active E_2	Active progesterone
3.	Placebo	Placebo	Placebo

Packages will be shipped to each center monthly for those subjects due for a renewed 3-month study drug supply in the following month. Each subject will apply a patch once weekly, take a tablet daily, and take a progesterone capsule from the 1st to the 12th day of each month. In each month in which cognitive studies are done during estrogen treatment (months 18 and 48), subjects will be asked not to take the progesterone/placebo capsules. Subjects and investigators will be blinded to treatment group. Subjects will return containers and any unused medications at each visit to be weighed (tablets, capsules) or counted (patches) to determine noncompliance. Subjects will also be asked about missed doses.

a. Estrogens – Study subjects will take oral CEE (Premarin® 0.45 mg daily) with a placebo patch or transdermal E_2 via skin patch changed weekly (Climara® 50μg/day and a placebo tablet) or placebo patches and tablets. Both subjects and investigators will be blinded to drug identity. Premarin® at doses of 0.3, 0.45 mg and 0.625 mg/day and Climara® at doses of 50 and 100 μg/day are FDA-approved for relief of menopausal symptoms and prevention of bone loss in menopausal women. As shown in Tables 1 and 2, above (pages

8 and 10, respectively), in a large observational study [52] 0.3 mg of CEE was both cardioprotective and appeared to reduce the incidence of ischemic stroke. A study examining systemic effects of estrogens has suggested approximate dose-equivalence for 50μg of transdermal E_2 with 0.3 mg of CEE and for 100μg of transdermal E_2 with 0.625 of CEE with regard to changes in urinary calcium excretion and vaginal epithelial maturation [92].

b. Progestin – Subjects receiving active estrogens will take Prometrium® (micronized progesterone USP encapsulated with peanut oil) 200 mg daily for the first 12 days of each month at bedtime. Subjects not receiving an active estrogen will take placebo capsules. Prometrium® is USFDA approved for antagonism of estrogen effect on the endometrium in women taking MHT. According to a review of studies of oral progesterone in menopausal women [93], use of progesterone, the progestational steroid produced by the human corpus luteum, minimizes side effects seen with synthetic progestins. The bioavailability of oral micronized progesterone is similar to that of other natural steroids, and interindividual and intraindividual variability of area under the curve is similar to that seen with synthetic progestins. Long-term protection of the endometrium by Prometrium given as 200 mg/day for 12 days/month has been established. In a randomized double-blind clinical trial, 358 postmenopausal women with uterus intact were treated for up to 36 months with Prometrium® 200 mg/day for 12 days per 28 day cycle in combination with CEE 0.625 mg/day (n=120); with CEE 0.625 mg/day alone (n=119); or with placebo (n=119). The group receiving Prometrium® showed a significantly lower rate of hyperplasia (6%) compared with the group on estrogen alone (64%). (see package insert, attached). No patients developed endometrial cancer. Oral micronized progesterone at a dose of 200 mg/ day is well tolerated, with the only specific side effect being mild and transient drowsiness.

3. Study Procedures

a. Recruiting and Screening– Subjects will be recruited from local ambulatory, home-dwelling populations by advertisements for normal volunteers in print and broadcast news media, both local and national, posting of flyers at the local hospital and clinics, and at public gathering places such as community centers, by solicitation of referrals from physicians at menopause and women's health clinics at the participating study

centers, and/or by mass mailing of recruiting brochures to eligible candidates identified from commercially available mailing lists. Initial contact will be by phone call from the candidates to a trained study screener who will collect basic information determining eligibility for study and arrange a first (screening) appointment. This telephone interview to establish whether women are qualified in terms of basic inclusion and exclusion criteria (age, smoking, body mass index, nut allergy, general health), followed by a screening visit at which informed consent will be solicited. After informed consent, women will undergo a complete medical and reproductive history, and physical examination including height, weight, waist and hip circumference measurements, breast examination, pelvic examination, and PAP smear. At the time of pelvic examination a vaginal ultrasound study will be obtained to rule out endometrial hyperplasia. Screening procedures will include administration of the Beck Depression Scale (BDS) and the MiniMental State Examination (MMSE) to exclude depression and dementia, respectively. Blood (38 ml) will be drawn and a urine sample taken for the screening laboratory profile (Chemistries, TSH, CBC, U/A, FSH, E_2, lipid profile) and a resting electrocardiogram (ECG) will be obtained and evaluated. Coronary calcium study will be measured by x-ray tomography as a final screening procedure (see below) and the first of two baseline carotid intimal medial thickness (CIMT) determinations by carotid ultrasound will be done at a screening visit and the second baseline CIMT within 6 weeks of the first.

Subjects will excluded for use of active estrogens, SERMs, or supplements known to have significant estrogenic activity, such as isoflavones, soy extracts within the past 3 months. Subjects will be excluded for endometrial thickness on ultrasound >5 mm, unless follow-up endometrial biopsy is negative for complex endometrial hyperplasia with or without atypia and for endometrial cancer. Subjects with LDL ≥ 190 mg/dl or triglyceride ≥ 400 at screening will not be eligible for study. For all other subjects, a Framingham Risk Score [94] will be calculated based on data obtained at screening. Any woman who meets current NCEP criteria for treatment with a lipid-lowering drug [95] will be informed of that fact, and referred to her personal physician. If treatment with a lipid lowering drug or red rice yeast is initiated, subject will not be eligible for study, otherwise she will still be considered eligible. Subjects initiating treatment with

lipid lowering drugs (statins, fibrates) or herbal preparations after randomization will be continued on study medication.

Women qualified according to results of the above examinations and willing to participate will return for safety imaging studies, which will consist of:

i. Mammography- (if not done in previous 12 months)
ii. X-ray or electron beam tomography to determine coronary calcium

Those whose endpoint measurements and safety study outcomes fall within specified acceptable limits (see inclusions and exclusions) will be randomized into the study.

b. Randomization and blinding- Ninety subjects will be randomized in six blocks of 13 and one block of 12 at each center, using a random number table to sort subjects into 3 groups. The assortment will be weighted to increase numbers in the placebo group (n=34) vs. transdermal E_2 and oral CEE groups (n=28). Study drugs will be supplied to centers identified only by the subject's unique study ID number. Therefore, neither research subjects nor investigators will know which agents subjects are receiving. The research pharmacist, the national study coordinator at the Coordinating Center, and one non-investigator monitor at each study center will be unblinded as to treatment.

c. Study Visit 1- Women will have blood drawn (124 ml) for secondary endpoint studies (lipids, hormones, coagulation factors, inflammatory markers) and for DNA banking. Subjects who decline permission for DNA banking will still be allowed to participate in the study. Women will be sent to the ultrasound imaging laboratory for acquisition of CIMT images (30 min) and the DEXA center for scanning of bone and body composition (30 min). The cognitive, affective, and quality of life profiles and diet questionnaires will be administered (approximately 2 hours). Finally, subjects will be instructed regarding use of study medications, including symptoms of adverse events to be aware of and study drug will be dispensed.

d. Follow-up

i. Short safety visits – At 3 month intervals women will visit the study center to return any unused study drug and receive a new 3 month supply. At each such visit women will return a completed 3 month bleeding diary and respond to a structured questionnaire regarding compliance, symptoms of menopausal estrogen deficiency, heart disease, stroke, deep vein thrombophlebitis, pulmonary embolus, cholecystitis,

breast changes or pain, edema, bloating, nausea or vomiting, headache, weight or appetite changes, and libido. If a woman is unable to attend a particular short safety visit, she will be contacted by phone to respond to the follow-up history questionnaire and her renewal drug supply will be mailed to her.

ii. Long safety visits- At months 6, 12, 24, 36, and 48 in addition to the follow-up history questionnaire, an interim physical examination, including height, weight, waist and hip circumference measurements, will be performed, which, except for month 6, will include a pelvic and breast examination. An EKG will be obtained and read at yearly (but not 3 and 6 months) visits. Results of annual mammography and Pap smear will also be obtained and evaluated at yearly visits.

iii. Visit/study endpoints – At baseline, 12, 36, and 48 month visits blood (116 ml) will be drawn fasting before 11 am for measurement of secondary endpoint studies listed above (with the exception of the 8 ml for DNA). Blood will be drawn while participants are on estrogen alone at 12 and 48 months and while they are taking progesterone (days 4-12) at 36 months. At baseline and 12 months, a first morning void urine sample will be acquired and stored for measurement of oxidative stress markers (isoprostanes, DNA damage products, protein damage products) and other ancillary studies. DEXA will be done at baseline and repeated at 12, 24, 36, and 48 months; two replicate CIMT measurements (3 days to 6 weeks apart) will be done at baseline, and one at 12, 24, 36 and 48 months with a replicate CIMT scan at exit or 48 months, whichever comes first, cognitive/affective studies at baseline 18, 36, and 48 months; and coronary calcium at baseline and 48 months only.

iv. Study scheduling timeline- Recruitment will begin in July, 2005 and continue through March, 2008. Randomization of subjects will begin Sept. 1, 2005. Exit study visits for those completing the protocol will begin in August. 2009, starting with the subjects first randomized and continue with a cut-off date for the last study visit of Feb. 28, 2012. Thus, subjects randomized early may be studied on protocol for as long as 51 months and subjects randomized late for as little as 40 months. Average length of treatment for subjects completing protocol is expected to be 48 months with a “window” of + 3 and -5 months.

Figure 1- Calendar for study- Calendar for study- months in gold = recruiting; months in yellow = recruiting and randomizing; months in green = final exit (48 month) visits; months in rose = data clean up, statistical analyses, drafting of initial study reports.

	Jan	Feb	Mar	Apr	May	Jun	Jul	Aug	Sept	Oct	Nov	Dec
2005									1	2	3	4
2006	5	6	7	8	9	10	11	12	13	14	15	16
2007	17	18	19	20	21	22	23	24	25	26	27	28
2008	29	30	31	32	33	34	35	36	37	38	39	40
2009	41	42	43	44	45	46	47	48	49	50	51	52
2010	53	54	55	56	57	58	59	60	61	62	63	64
2011	65	66	67	68	69	70	71	72	73	74	75	76
2012	77	78	79	80	81	82						

v. Time windows for visits- Time windows of ± 2 weeks will be acceptable for 3 month study visits and windows of ± 3 weeks for completion of all procedures at annual or other study visits during which endpoint procedures (e.g. CIMT, blood draws, etc.) are obtained. For cognitive/affective studies a longer time window for testing (± 6 weeks) will be allowed. At the 36 month time point cognitive testing will be carried out between day 4 and 12 while the subject is taking progesterone or placebo capsules.

vi. Exit visits- at 48 months, or when a subject leaves the study early, a complete physical examination will be performed and a full set of safety laboratory assays will be obtained and recorded. In addition two exit CIMT ultrasound study will be done if 6 months or more have elapsed since the most recent CIMT study and an exit coronary calcium scan will be done if the exit visit occurs 3 or more years after randomization.

4. Outcome Measures

a. Carotid intimal medial thickness by high resolution B-mode ultrasonography-

Justification for method - Several studies [42, 96-98], have shown that non-invasive determination of CIMT by ultrasound is a safe, sensitive, and accurate method for the estimation of degree of subclinical atherosclerosis. Repeated measurements of CIMT with the computerized edge detection method of image analysis reduce the sample size necessary for study [99]. Comparison of CIMT data with measurements in excised vessel segments has confirmed the accuracy of ultrasonographic estimates of carotid atherosclerosis [100, 101].

Measurement of CIMT is informative regarding coronary artery status. Carotid artery atherosclerosis is significantly correlated with the degree of atherosclerosis in coronary arteries at autopsy [102]. There is a strong relationship of carotid wall thickness with angiographic presence of coronary artery disease [103-105] and with confirmed history of CAD [106] in both men and women. In addition, CIMT progression is associated with clinical progression of atherosclerotic disease and reduction in CIMT progression mirrors reduction in clinical events as observed in primary [107-111] and secondary [96-98, 112-114] prevention trials of lipid-lowering therapy.

There is also a significant correlation of CIMT progression with coronary artery disease progression measured by serial coronary angiography [115, 116] and the relationship between clinical events and progression of CIMT is as strong as the relation between events and progression of coronary atherosclerosis as determined by angiography [116]. These data are consistent with other studies in which CIMT has been found to be a strong predictor of cardiovascular events [115, 117-120].

Finally, several atherosclerosis intervention trials have demonstrated that a 2 to 3 year intervention period is generally sufficient for detecting treatment group differences [121].

a. ***Methodology-*** CIMT B-mode carotid artery images are acquired at each study center by certified ultrasound technicians trained at the core CIMT center (PI: Dr. Howard Hodis, USC) to perform a standard acquisition sequence. Electrocardiogram (ECG), external time code information and ultrasound images are simultaneously recorded with a videotape recorder. Image acquisition procedures are optimized for minimal measurement variability [Selzer, 1994 #121; Beach, 1989 #144; O'Leary, 1987 #146; Wendelhag, 1991 #147]. The ultrasound power, echo detector gain and dynamic range are recorded to establish identical conditions for serial examinations. All instruments are high-resolution imagers with a linear array 7.5 MHz probe. Electrocardiogram (EKG) external time code information and ultrasound images are simultaneously recorded on digital videotape and processed images are stored on CD’s. A copy of each individual's baseline image is used as a guide to match the vascular and surrounding soft tissue structures for follow-up examinations and reproducing the probe angle. All images are evaluated by an experienced investigator at the core CIMT study center.

For image acquisition, subjects are placed supine and positioned in a 45 degree molded head block to present the optimal angle for ultrasound examination. Using B-mode, the right common carotid artery is imaged in cross section and the scan head moved laterally until the jugular vein and common carotid artery are stacked with the former above the latter. In this position, the central image line passes along the common diameter of both vessels. The scan head is then rotated around the central image line 90 degrees maintaining the jugular vein stacked above the common carotid artery while obtaining a longitudinal view of both vessels. In this longitudinal view, the common carotid artery far wall is horizontal. The proximal portion of the carotid bulb is included in all images as a reference point for standardization of CIMT measurements. Stacking the jugular vein and common carotid artery determines a repeatable probe angle, which allows the same portion of the wall to be imaged at each examination [122], and decreases measurement variability [99]. Images are acquired from the carotid bulb and internal carotid artery, but emphasis of ultrasound imaging is on the distal centimeter of the CCA because least variability occurs in this area [123]. The far wall is used for statistical purposes since measurement of near wall thickness is less accurate [124].

Each ultrasound scan is recorded on tape and processed images are stored on disks. The ultrasound power, echo detector gain and dynamic range are recorded to establish identical conditions for serial examinations. This establishes a standardized instrument setup for all tests within a subject. A copy of each individual's baseline image is used as a guide to match the vascular and surrounding soft tissue structures for follow-up examinations and reproducing the probe angle. The brightness and contrast settings of the image display are checked daily and standardized. These techniques have significantly reduced measurement variability between scans [99]. All images are evaluated by an experienced investigator at a single core CIMT study center (Howard Hodis, M.D., University of Southern California School of Medicine). CIMT technical personnel at each study center are trained in the laboratory of Dr. Hodis to standardize image quality and reduce variation among centers.

Clinical precautions – Because it is possible that in a few subjects CIMT may detect clinically significant carotid atherosclerosis, whenever the reading center detects a lesion of the carotid artery causing narrowing of the lumen of 20% or greater, this finding will be

reported to the study center PI so that the subject can be informed that she may require further diagnostic investigation to determine the extent of atherosclerosis."

b. Coronary artery calcium

Justification for method - The presence of calcium in atherosclerotic lesions is a marker for progression from simple fatty streaks (cholesterol infiltration and foam cells) to complex (inflammatory, fibrosed, necrotic) plaques. There is a direct relation between coronary calcium and histologic [125, 126], as well as with *in vivo* intravascular measures of atheromatous plaque [127]. The ability of EBT to accurately quantify coronary calcium has been validated in many studies [127-132]. In a recent study, coronary calcium scores were superior to the Framingham risk factors in predicting the measured proximal stenosis burden determined from coronary angiography [133]. Moreover, in a new cross-sectional study of 17,967 men and, women [134] there was an increased risk for prevalent CHD at all levels of coronary calcium scores >0, with the greatest increase occurring in patients with scores >95. The odds ratios for prevalent clinical CHD increased significantly across increasing quartiles of coronary artery calcium and scores in the fourth quartile were associated with an odds ratio of 33.8 for CHD.

The development of novel calcium volume scoring system [135] and novel ECG-gating algorithms have allowed for a higher degree of reproducibility between scans, making it possible to use EBT to detect changes in atherosclerotic plaque during sequential scans in individuals [136]. Although there are differences in the design of studies that have used X-ray tomography to track longitudinal changes, including the duration of follow-up and the method used for quantifying calcium (Agatson vs. volumetric method), recent studies are in agreement that EBT can be used successfully to track changes in coronary atherosclerosis over time [136, 137]. Percentile scores will be calculated using the Rochester Age and Gender Demographic database [138]. Coronary calcium scores in asymptomatic men and women increase by a mean of 33% per year, predicting that the coronary calcium scores would double every 2.5 to 3 years, and changes in calcium scores also predict the progression of coronary artery disease [137]. In a recent study progression of coronary calcium was associated with 5-13 fold greater risk of cardiac events [139]. Moreover,

individuals with hypercholesterolemia treated with statins have lower rates of atherosclerosis progression than those not receiving statins [137].

For the purposes of this study, coronary calcium is defined as a plaque of at least 3 contiguous pixels (area 1.02 mm^2) with a density of >130 Hounsfield units. The lesion (Agatston) score is calculated by multiplying the lesion area by a density factor derived from the maximal Hounsfield unit within this area. A total CAC score is determined by summing the individual lesion scores from each of 4 anatomic sites (left main, left anterior descending, circumflex, and right coronary). A volume score, independent of density is also calculated using a standard algorithm. A single experienced investigator, blinded to the group assignment and subject identity, interprets all the scans using commercially available software (Neo Imagery Technologies, City of Industry, CA). Inter-reader variability is assessed by a second reader in 5% of cases, and similarly, 5% of cases will be re-read to assess for intra-reader variability. Comparability among centers is assured by regular calibration using a standard phantom.

Either of two methods for obtaining calcium measures will be acceptable:

<u>Electron beam computerized tomography (EBT</u> - For measurement of coronary calcium, the entire length of the coronary arteries will be visualized without contrast using C150XP or C300 electron beam tomography scanners (GE/Imatron, Inc.). At least 30 consecutive images will be obtained at 3 mm intervals. Coronary calcium will be defined as a plaque of at least 3 contiguous pixels (area 1.02 mm2) with a density of >130 Hounsfield units. The lesion score will be calculated by multiplying the lesion area by a density factor derived from the maximal Hounsfield unit within this area. A total calcium score will be determined by summing the individual lesion scores from each of the 4 anatomic sites (left main, left anterior descending, circumflex, and right coronary). A volume score, independent of density, will be calculated using a standard algorithm. Density, Agatston score, volume score and number of lesions within the entire coronary tree will be assessed in each participant at each measure. Furthermore, quantification of mitral, aortic and aortic valve calcification will also be performed in each EBCT scan. A single experienced investigator, blinded to the group assignment or subject identity, will interpret all the scans using commercially available software (Neo Imagery Technologies, City of Industry, CA),

and inter-reader variability will be assessed with a second reader in 5% of cases, and similarly, 5% of cases will be re-read to assess for intra-reader variability.

Multidetector Computerized Tomography – Numerous manufactures and models are available; uniformity of equipment at participating sites is extremely unlikely. Since the specifications for each model and manufacturer are different, and since each patient will serve as her own control, the studies should be acquired in exactly the same fashion for each exam, using the same acquisition parameters. The minimum requirement will be 4 detector heads. Analysis of the data will be performed using the method described above for EBCT.

Procedure for Scan Acquistion- The technologist will instruct the subject on the importance of breath holding and immobility during scanning. An interpreter will assist in the instruction of subjects who are not fluent in English. All scanning will be done with a single breath hold. Total imaging time will be approximately 30 to 40 seconds. The technologist will instruct the subject to take three deep breaths, and then to hold his/her breath (at end-inspiration), while acquiring an 11 cm scout image, beginning 180 mm below the sternal notch. This will provide views of the chest on the image monitor at the operator console. From this, the technologist will check patient centering and choose the position for the highest scan (at the lower margin of the bifurcation of the main pulmonary artery).   The couch will be moved to the start position. The technologist will check subject and phantom positioning in the scout image. At least 10.5 cm of data in the z direction will be acquired with each scan and the scan field of view will be 35 cm for all scanners (to incorporate the phantom in the image). Since the specifications for each model and manufacturer are different, and since each patient will serve as her own control, the studies are acquired in exactly the same fashion for each exam, using the same acquisition parameters. Spiral scanners will use a partial scan tube rotation (~240 degree) with optimized reconstruction techniques that provide 250 – 300 msec temporal resolution in the center area of the scan field of view. For each scanner, the default settings will be as follows:

- Imatron EBT scanners: 130 kVp, 630 mA, scan time 100 msec, 3mm collimation, sharp reconstruction filter. For EBCT scans, prospective cardiac gating will be used with scanner triggering at 50% of the electrocardiographic RR interval. The EBCT

scanner table will scan after each table increment of 3 mm (sequential axial scans) . The technologist will acquire 40 image slices to ensure that the entire heart is scanned

- General Electric helical scanners are set at KV120 (mAs variable according to local protocol for body habitus, 500 msec). Siemens scanners will be set at 140 kV (mAs according to local protocol, 500 ms). Triggering is set at 50% of the R-R interval, using prospective gating, with image acquisition scan time set at 100 to 300 msec, and matrix to 512. The technologist will set the image slice thickness to 3 mm and will acquire 40 slice images. The technologist will use the 35 cm field of view and the sharp reconstruction kernel for all EBCT scans. Standard kernel will be used for all spiral scans. The GE helical scan data are reconstructed using a segmented scan reconstruction algorithm on the scanner console immediately following the study. Siemens scanners are set at (140 kV, mAs according to local protocol, 500 ms). This dependence on heart rate is needed in order to provide gapless continuous volume coverage. The equation is: pitch = 1.5 * (BPM/60).

Triggering for both GE and Siemens scanners will be at 50% of the R-R interval, using prospective gating, with image acquisition scan time set at 100 to 300 msec, and matrix to 512. The technologist will set the image slice thickness to 3 mm and will acquire 40 slice images.

The technologist will use the 35 cm field of view and the sharp reconstruction kernel for all EBCT scans. Standard kernel will be used for all spiral scans. The GE helical scan data are reconstructed using a segmented scan reconstruction algorithm on the scanner console immediately following the study. Spiral scanners will use a partial scan tube rotation (~240 degree) with optimized reconstruction techniques that provide 250 – 300 msec temporal resolution in the center area of the scan field of view.

c. Lipid risk factors for CHD

i. Total, HDL, and LDL cholesterol and triglycerides- Numerous studies support the hypothesis that high levels of LDL cholesterol and low levels of HDL cholesterol are associated with increased risk of CVD [140] and that these lipid particles as well as triglycerides [141] play important etiologic roles in atherosclerosis. Moreover

interventions that decrease LDL cholesterol and/or increase HDL cholesterol have been demonstrated to decrease rates of CHD [142]. Finally, multiple studies have demonstrated that both oral and transdermal estrogen treatment tends to lower LDL and raises HDL cholesterol levels, although the transdermal route may have somewhat less effect on HDL-cholesterol [143-146]. Lipids will be measured by a standard multichannel analyzer method using NCEP standards.

ii. LDL subfractions and Lp(a) – The excess risk of CHD associated with high level of LDL cholesterol appears to be mediated by the small dense LDL particles (LDL III), with little or no risk associated with the larger, less dense fractions (LDL I + II) [147]. In one study [148], a combined regimen of 0.625 mg/day of CEE with continuous MPA at 5 mg/day caused a significant reduction in LDL cholesterol levels (11.1%; P<0.01), but mainly as a result of a decrease in the LDL I + II subfraction, a result similar to that seen with oral E_2 monotherapy in which the observed reduction in LDL was due to a decrease in the light LDL-subfraction with an apparent shift in distribution towards the heavy subfraction, but no absolute increase in the latter [149]. However, in a another study using 0.625 mg of CEE and 2.5 mg of MPA daily in postmenopausal women with type 2 diabetes mellitus, no changes were observed in the average diameter of VLDL, LDL, or HDL particles; or the cholesterol concentrations of LDL subfractions [150]. A modified lipid fraction, Lp(a), has been reported to be a CHD risk factor, independent of LDL- and HDL cholesterol levels [24]. Estrogen's effect to lower Lp(a) may contribute to cardioprotection [151]. LDL subfractions and Lp(a) will be measured by a high resolution microvolume Vertical Auto Profile (VAP) method for the simultaneous measurement of cholesterol in all lipoprotein classes, including lipoprotein(a) (Lp(a)) and intermediate density lipoprotein (IDL) [152]. This VAP-II method uses a nonsegmented continuous flow (controlled-dispersion flow) analyzer for the enzymatic analysis of cholesterol in lipoprotein classes separated by a short spin (47 min) single vertical ultracentrifugation. Cholesterol concentrations of high (HDL), low (LDL), very low (VLDL), and intermediate (IDL) density lipoproteins, as well as Lp(a), are determined by decomposing the spectrophotometric absorbance curve, obtained from the continuous analysis of the centrifuged sample, into its components using software

developed specifically for this purpose. Analysis by VAP-II is rapid and sensitive (as little as 40 p1 plasma is required per assay). Total and lipoprotein cholesterol values obtained by VAP-II correlate well with the values obtained by Northwest Lipid Research Laboratories (NWLRL). VAP-II Lp(a) cholesterol values also correlated well with the Lp(a) mass values obtained by an immunoassay technique performed at NWLRL (r = 0.907). The reproducibility and accuracy of the method are within the requirements of the CDC-NHLBI (Centers for Disease Control-National Heart, Lung, and Blood Institute) Lipid Standardization Program

d. Blood Coagulation Indicators - In a large metanalysis [153] HRT was associated with decreases in levels of fibrinogen, factor VIII, antithrombin III, and proteins C and S, and increased plasminogen. HRT was associated both with changes that could explain the increased rate of venous thrombotic events, and also with some changes that could account for beneficial vascular effects. The addition of progestins induced favorable changes in some cases and transdermal use appeared to be associated with less potentially harmful effects than oral regimens. In another study [154] of 2 mg of E_2 valerate combined after 3 months with 10 mg of medroxyprogesterone for 10 days every third month, in the HRT group, Factor VII increased, whereas fibrinogen, antithrombin III, PAI-1, and total protein S decreased at 3 months. By 12 months, fibrinogen, total protein S, tissue plasminogen activator and antithrombin III were decreased, leading the authors to conclude that effects of HRT on coagulation are more pronounced early and with unopposed treatment.

We will measure serum and plasma markers which both potentially predict risk of thrombosis and which reflect the ongoing activation of the coagulation system. These factors will be measured at baseline and blood will be processed on an ongoing basis throughout the study to evaluate effects of treatment on the ongoing activity of the coagulation cascade. Because we anticipate that changes in the levels of the time-dependent markers will be continuous, it is proposed that these markers will be evaluated at multiple time points in all enrolled patients.

Markers to be analyzed at baseline and with periodic blood assessments throughout the study include the levels of

i. Activated factor XII

ii. Tissue factor
iii. Anti-thrombin III
iv. Soluble CD-40
v. D-Dimer
vi. Tissue plasminogen activator

These are markers of available clotting substrate, functional circulating thrombin, thrombin activation, and platelet activation. [155]. All the factors listed will be measured at the KEEPS core laboratory.

e. Inflammatory markers –

Research over the last 10 years has increasingly identified a role for inflammation as an important mechanism underlying formation of atherosclerotic plaques [156, 157]. A variety of markers or mediators of inflammation have been suggested as risk factors for coronary artery disease, independent of the lipid risk factors listed above. Estrogen treatment has been shown to affect circulating levels of a number of these factors. For example, in the PEPI trial [158] estrogen treatments increased concentrations of C-reactive protein by 85% compared with baseline. In studies comparing oral and transdermal estrogens oral treatment increases levels of CRP [55] and decreases plasma levels of homocysteine [25] whereas transdermal does not. A prospective, nested case-control study of women in the WHI hormone trial [113] assessed the association between baseline levels of CRP and interleukin 6 (IL-6) and incident coronary heart disease (CHD) and examined relationships between vascular risk and baseline use of HRT, CRP, and IL-6 levels. With occurrence of first myocardial infarction or death from CHD as the primary variable, median baseline levels of CRP and IL-6 were significantly higher among cases compared with controls and odds ratios for in the highest vs. lowest quartile were 2.3 for CRP (95% CI 1.4-3.7; P for trend =.002) and 3.3 for IL-6 (95% CI, 2.0-5.5; P for trend <.001). Use of HRT was associated with significantly elevated median CRP levels but no association between HRT and IL-6 was observed.

The following markers of inflammation will be measured at the inflammation core laboratory. Specific determinations will be as follows:

i. C-reactive protein (CRP)

ii. Interleukin-6 (IL-6)
iii. Plasminogen activator inhibitor I (PAI-1)_
iv. Homocysteine

f. Hormones – Hormone measurements will be conducted on blood samples taken in the morning at baseline and between days 13 and 30 of the month (when subjects are taking no oral progesterone) at months 12, and 48 and while on progesterone (between days 4-12) at month 36. Serum levels will be estimated by standard immunofluorescent assay methods at the core hormone laboratory. Hormones and hormone binding proteins to be determined are:

i. Estradiol
ii. Estrone
iii. Progesterone
iv. Testosterone
v. Sex hormone binding globulin

g. Storage and use of plasma and serum samples- Serum and plasma volumes beyond those needed for the above-specified core studies will be stored frozen in convenient aliquots at -80C at the KEEPS core laboratory facility and made available to investigators for KEEPS ancillary studies. Such studies will be limited to investigations relevant to the underlying concept of the KEEPS including beneficial and harmful actions of estrogens and factors potentially contributing to cardiovascular risk such as (but not limited to) inflammation, coagulation, lipid metabolism, and insulin action and resistance. Samples may be retained in the KEEPS plasma bank for up to 10 years after the completion of the KEEPS core protocol. At the end this period remaining samples will be destroyed, or, if requested, returned to the KEEPS study center institutions from which they were received. Samples will be supplied to ancillary study investigators "stripped" of identifying information (i.e. with randomization code referable to treatment group in the KEEPS database, but no personal identifiers accessible to the investigators.).

h. Genetic studies – Buffy coat nucleated cells will be obtained from blood samples taken at the baseline visit. DNA will be extracted by standard methodology and stored indefinitely for future genetic studies at the DNA center. Studies will be limited to

evaluation of allelic variation of the estrogen receptor alpha and beta genes, identification of alleles of genes related to clotting (e.g. Leiden factor V), inflammation, and lipid metabolism and other genes known or suspected to influence CHD risk (e.g. Apo-E). Studies may extend to other genes involved in or potentially related to estrogen metabolism and action. No DNA samples will be released to outside investigators with any identifying information.

i. Determinations of bone density and body composition- Dual X-ray absorptiometry (DEXA) remains the most accurate and reproducible method for determining bone density at multiple sites [159-161]. Estrogen treatment has been shown to reduce bone calcium loss as measured by DEXA in numerous prior studies [162-165]. Appendicular lean and fat mass content, percent of fat mass, total body muscle mass can also be analyzed from a single DEXA scan [161, 166-168]. The precision of regional body composition using DEXA is less than that for the whole body [161]. Appendicular skeletal muscle mass can be derived as the sum of the fat-free masses of the arms and legs [166]. The precision, reproducibility, and the ease with which DEXA scanning can be performed make it attractive in the proposed study population. DEXA scanning will be done using either GE Lunar, Prodigy, or Hologic dual X-ray scanners (depending on the study center). For modern scanners, the procedure requires less than 10 minutes. For AP image acquisition, subjects will recline in a supine position. For vertebral density, lateral scans will be obtained by placing subjects on their sides, supported by foam pillows. QA will be done daily with a spine phantom, and DEXA machines will be compared across centers quarterly using a common phantom. All technicians will be ISCD certified and certified to do research.

j. Cognitive function- A comprehensive battery of standardized neuropsychological tests will be administered by an individual trained by the core cognition center (U. of Wisconsin, PI, Dr. Sanjay Asthana). Ideally, this individual will have a background, in psychology, i.e., be a psychologist or postgraduate student in psychology. The battery will consist of tests shown previously to be affected by estrogen treatment [169, 170], to include: the Modified Mini-Mental State Examination (MMMSE), Primary Mental Abilities-Vocabulary, Profile of Mood States, Beck Depression Inventory, Prime MD, Memory Function Questionnaire, California Verbal Learning Test-2, NYU Paragraph

recall, Benton Visual Retention Test, Prospective, Verbal Fluency FAS/Animals/Fruits/Vegetables Memory Test, Trail Making Test version A & B, Stroop Letter-Number Sequencing WMS-3, Digit Span WMS-3 Test (Golden Version, Digit Symbol, *3D Mental Rotation,* Visual Sensitivity Test, and the Utian Quality of Life Questionnaire. Descriptions of these tests with appropriate citations are in the attached Addendum 1, entitled "*KEEPS Neuropsychological and Affective Battery: Description of Tests*." These assessments will be carried out at baseline, during estrogen treatment at 18 and 48 months and during progesterone treatment at 36 months. With the exception of the Primary Mental Abilities Vocabulary Test, the complete cognitive and affective battery will be administered at baseline, 18, 36, and 48 months. The Primary Mental Abilities Vocabulary Test will be administered at baseline only.

k. Quality of life - This will be assessed at baseline, 18, 36, and 48 months using the Utian Quality of Life (UQOL) Scale, a validated self-report instrument designed to objectify quality of life in otherwise healthy postmenopausal women [171]. In addition, ,Nutritional status will be assessed using the Rapid Eating Assessment for Patients (REAP) at these same times. Sleep quality will be assessed using the Pittsburg Sleep Quality Index (PSQI) at baseline 6, 18, 36, and 48 months.

l. Affect - will be assessed by the Beck Depression Inventory (BDI The Profile of Mood States (POMS) affective scale and the Prime MD, administered on the same schedule as for cognitive testing, baseline, 18, 36, and 48 months.

m. Libido and sexual activity – this will be assessed using the Female Sexual Function Inventory (FSFI) [172] on the same schedule as for cognitive testing.

4. Statistical Analyses and Sample Size Estimation

a. Overview of analysis for primary endpoint

Rates of progression of CIMT in each treatment group will be estimated using repeated measures multivariate linear mixed models. We will attempt to obtain full follow-up data on all randomized participants and the primary analysis will be intention to treat. All data points (regardless of compliance to study drug) will be included and there will be no imputation of missing values for CIMT. The statistical tests will assess the statistical

significance of the treatment by time interaction term. Separate analyses will be done for the oral HT vs. placebo and for the patch vs. HT. In addition to the primary ITT analysis, secondary per-protocol analyses will be performed as specified in a detailed analysis plan to be written prior to study unblinding.

a. Overview of secondary analyses

   i. Changes in EBT coronary and aortic calcium will be analyzed as continuous variables in a manner similar to that described for CIMT. In addition, distributions of women classified as showing significant progression vs. no progression of coronary and aortic calcium levels will be tested for significance by comparing oral and transdermal estrogen groups with the placebo group using Fishers exact test.

   ii. In order to investigate the extent to which measured risk factors predict arterial response to MHT we will employ augmented linear mixed models to determine whether rates of change of CIMT and EBT calcium are modified by baseline values for, and with observed changes in, risk factor measurements

   iii. Analysis of continuous coagulation markers: The mean levels of each of the continuously measured markers of activation of the coagulation cascade will be presented descriptively. The levels of these markers between patients allocated to the study medication and placebo will be compared using linear mixed models. Since there are no reliable data to hypothesize how study drug will influence the level of these markers, this analysis will be considered exploratory. However, if we demonstrate that the levels of these markers do change differentially between the study groups they may be evaluated in future studies to determine if they have predictive power for the development of thrombotic or other complications.

b. Interim analysis

We will not perform a formal interim analysis (other than that required for the DSMB to evaluate safety) with any intention of stopping the trial. However, in order to apply for additional funding, it may be necessary to tabulate results part way through the trial. If this is necessary, a formal procedure will be developed to make certain that all investigators and staff remained blinded to study results. Only persons who have no contact with patients and who also are not involved with making decisions about study efficacy or safety endpoints will be eligible to be unblinded. Each unblinded person will be required to sign a confidentiality

statement and an ongoing list will be kept and evaluated by the DSMB as to who has access to what level of blinded data or results.

c. Power analyses

   i. CIMT - Our primary analysis will use a repeated measures analysis to compare change in CIMT in the actively treated groups to placebo. This analysis will be performed separately for the oral HT vs. placebo and patch vs. HT. CIMT measurements will be done in duplicate at baseline, single studies at 24, and 36 months and in duplicate at 48 months (closeout) or exit, for subjects leaving the study before 48 months. In order to assess the power for the study, we need to estimate the true difference in rate of change between the treatment groups, as well as the variance and covariance of the repeated CIMT measurements.

   ii. **Difference in the rate of change.** We base our estimate of the treatment effect on two studies, which have CIMT as a primary endpoint: the EPAT study of HT vs. placebo (42) and a study of pravastatin vs. placebo [173]. In EPAT there was a difference of <u>0.013 mm/year</u> and in the MacMahon study, a difference of 0.062 mm over 4 years <u>(0.015mm/year)</u>. Based on these data, we base the power calculations on a difference of 0.008 mm/year in the increase of CIMT between the HT group and placebo. This is approximately 60% of the difference observed by Hodis and colleagues in the EPAT study and is about ½ of that seen in the MacMahon study.

   Variance and covariance of CIMT: The rate of change will be estimated using a repeated measures linear mixed model [174] and estimation of power requires that we specify the form and parameters of the covariance matrix of the repeated outcome measurements. Since there is substantial measurement error in the CIMT measurement, we believe the primary source of between-measurement variation will be measurement (not true biologic) variability and we have therefore assumed that the correlation between any two measurements will be equal, regardless of the time interval between them. Based on the EPAT study, we estimate that the cross-sectional standard deviation will be 0.15 mm and based on the MacMahon study, we estimate a correlation between measurements of 0.5. Note that the correlation may be somewhat higher in which case we will underestimate the true power of the study.

Statistical parameters: We calculate power for the study based on a recruited number of 720 participants (272 to placebo and 224 to each active treatment group), a significance level of 0.05 (two-sided, not adjusted for multiple comparisons) and a loss to follow-up rate of 4% per year (approximately 17% for the expected mean follow-up of 4.33 years).

Based on the assumptions detailed in the paragraphs above, we estimate that we will have a power of 92% for the primary analysis. The table below shows the power of the study under varying assumptions about the effect size (from 0.005 to 0.011/year) and for correlations between measurements.

Table 3. Power for varying assumptions about treatment effect and correlation between CIMT measurements

	Correlation between CIMT measurements		
Difference between treatments for CIMT (mm/year)	0.5	0.6	.07
.005	56%	65%	77%
.0065	78%	86%	94%
.008	92%	96%	99%
.0095	98%	99%	>99%
.0110	>99%	>99%	>99%

iii. Coronary calcium - The primary analysis anticipated for this measurement is an estimate of the difference in numbers of women progressing from no or non-significant to significant amounts of coronary calcium in each group (i.e. a non-continuous distribution). In published data from large observational studies, the magnitude of protection against coronary events varies from 40% to 60% [5, 12, 14, 18]. According to Raggi et al. [90] over a 4 year period approximate 4% of women aged 45-49 and 10% of women aged 50-54 progress from no coronary calcium to the 90th percentile and an additional 4% of women 50-54 years of age progress from the 90th to the 75th percentile. If we, therefore, assume an 18% progression rate in untreated women, and 272 in placebo and 224 in each active treatment, with a power of 0.9, we will be able to detect a reduction of about 50% (to about 9%) in an active treatment group.

B. Safety Monitoring and Procedures for Protecting Against Risks

a. General risks-

i. Blood drawing – Risks of inserting needles or catheters into veins include moderate pain, bleeding, and hematoma. Vary rarely, serious complications such as a thrombosis or infection may occur. Occasional subjects become hypotensive when blood is drawn. To reduce risks sterile all blood drawing will be done by experienced medical personnel, sterile disposable needles will be employed and the skin will be prepared with an antiseptic to avoid risk of infection.

ii. Blood loss- Over the entire 4 year course of the study, approximately 700 ml of blood will be drawn. The most taken at any one visit will be 165 ml. Intervals between blood draws will be 3 months or more in every case. Therefore, this study has little to no risk of causing anemia (low blood counts).

b. Specific risks-

Table 4. ESTIMATED EXCESS RISK OF ADVERSE EVENTS IN EACH KEEPS ARM AFTER 4 YEARS

	**Breast Cancer**			
Risk =12.9/10,000 woman years		Risk	New	Excess
Group	Woman Years	ratio	Cases	Cases
Placebo	1260	1	1.6	-
Oral E	1169	1.3	2.0	0.4
Patch E	1169	1.3	2.0	0.4
	**Thromboembolic Disease**			
risk= 10.0/10,000 woman years		Risk	New	Excess
	Woman Years	ratio	Cases	Cases
Placebo	1260	1	1.3	-
Oral E	1169	2.1	2.5	1.2
Patch E	1169	1.6	1.8	0.6
	**Coronary Events**			
risk= 5.3/10,000 woman years		Risk	New	Excess
	Woman Years	ratio	Cases	Cases
Placebo	1260	1	0.6	-
Oral E	1169	1.2	0.8	0.2
Patch E	1169	1.2	0.8	0.2
	**Stroke**			
Risk= 11.4/10,000 woman years		Risk	New	Excess
	Woman Years	ratio	Cases	Cases
Placebo	1260	1	1.4	-
Oral E	1169	1.4	1.8	0.4
Patch E	1169	1.2	1.6	0.2

i. Estrogen and progestin use- The potential serious risks of E + P include breast cancer, endometrial cancer, myocardial infarction, ischemic stroke, thrombophlebitis, pulmonary embolus, and cholelithiasis/cholecystitis. The results of the WHI trial have also raised questions regarding possible adverse effects of MHT on dementia and cognitive function [69, 72]. In order to quantify

risks in the proposed study, we have obtained, from the published epidemiologic literature estimates of spontaneous age-appropriate incidence rates, (events/10,000 women/year) of clinical adverse events thought to be associated with, or increased by, estrogen treatment. In Table 4, using our year-by-year estimates of number of women active in our trial, we have estimated the total number of woman-years in each treatment arm and then used the published endogenous rate and the published estimated relative risk (RR) rates for estrogen-treated groups in the WHI and other studies to calculate the number of excess cases expected in the KEEPS after 4 years. Risk estimates for cardiovascular complications below are generally "worst case" because we propose to use lower doses of estrogens and randomize lower risk women (younger, non- or light smokers, less obesity) than those from whom the relative risk estimates were derived.

1. Myocardial Infarction- In both the HERS [44] and the E+P arm of the WHI study [91] trials there were excess CHD events in the first 1-2 years. In contrast, a recent report [175] combined data from two randomized controlled trials of European women younger than those in the latter two trials (mean age 53.6 years; average duration from last menses 4.9 years), who were treated with placebo (n=284 patient years) or varying doses (0.3, 0.45, or 0.625 mg/day) of oral CEE with and without medroxyprogesterone acetate (n=3,577 patient years), during the first year of treatment there was one cardiovascular event in the placebo group (3.0/1000 patient years) and none in the estrogen-treated groups, values not significantly different from the expected rates of about 2.0/1000/year. The E-only arm of the WHI [86] also did not demonstrate any early excess of CHD events and showed a non-significant reduction in events in the younger (age 50-59) year old women. To minimize CHD risk, women with a history of myocardial infarction or angina or a coronary calcium score > 50 by EBT at screening will be excluded from study. Women with a complaint of chest pain consistent with angina or prior MI will be referred to their personal physician and will only be admitted to the study on submission of a report showing normal coronary function during a

non-invasive imaging test (i.e. stress echo or nuclear study).

2. Stroke – There were 29 cases per 10,000 patient years in women treated with oral estrogen vs. 21 in placebo-treated women in the WHI E+P study, giving a risk ratio of 1.38 [47] and a similar risk ratio of 1.44 in the E-only arm of the WHI [86]. However, there was no significant excess of strokes in the 50-59 year old women in the latter report. We calculated the expected number of new strokes in women 42-58 as 11.4/10,000 per year assuming 20% of the study population will be African-American [176, 177]. With approximately 1,575 patient years for the placebo group, we would expect 1.8 cases. A risk ratio of 1.38 gives 2.3 expected cases in the oral estrogen group and, assuming half the excess risk for transdermal estrogen, 2.0 cases in the latter group for a total excess of 0.7 additional cases over the 4-year course of the study. Data reviewed above (see Table 2) suggests that the lower dose of oral estrogen proposed for this study (and presumably the transdermal estrogen) should produce less excess risk. In addition because younger women in the E-only arm of the WHI and the combined data report in European women [175] showed no excess stroke risk [86], we believe that even the estimate of 0.7 excess cases is pessimistic. In order to reduce stroke risk, no woman with a history of stroke or TIA will be admitted to the study. In addition, we will not study women with uncontrolled hypertension, will monitor blood pressure regularly during the trial, and will recommend antihypertensive therapy for blood pressure elevation, as appropriate.
3. Thromboembolic disease – MHT may be associated with as much as a doubling of risk of deep vein thrombophlebitis (DVT) and pulmonary embolus (PE) over a 6 year period. Occurrence was 34 cases per 10,000 patient years in women treated with oral estrogen vs. 16 placebo-treated older women in the WHI E+P study [47], for a relative risk of 2.1. with an increased relative risk at 1.33 in the E-only arm [86]. However, the combined rate of occurrence of DVT and PE at younger (42-58 year) ages is estimated at only 10/10,000 women/year [178, 179]. This is similar to the incidence of thromboembolic disease in the estrogen-treated younger European women in the study cited

above of 8.4/10,000 patient years [175]. Given approximately 1,575 patient years in the placebo group in the proposed study, we would expect 1.6 total cases in the placebo group and no more than 3.1 in the oral estrogen group. Assuming transdermal estrogen to have about half the adverse effect on clotting of oral estrogen, we have estimated another 2.3 cases in the transdermal group, for a total excess of 2.2 cases of thromboembolic disease over the 4 year course of the study. To reduce this risk, women with a history of deep vein thrombosis (DVT) or pulmonary embolus (PE) will be excluded from study. In addition, due to the interaction of oral estrogen with known prothrombotic alleles, such as Factor V Leiden or prothrombin G20210A, to increase risk of DVT and PE [180, 181], women known to carry one of these prothrombotic mutant genes will be excluded, even if they have no personal history of thromboembolic disease. All women will be warned about and monitored for symptoms of DVT or PE and any diagnosed episode of either will require discontinuation of study medications.

4. Breast cancer - use of estrogen containing MHT for greater than $\underline{4}$ years has been associated in some studies with modest increases in breast cancers with relative risks on the order of 1.1 to 1.3 compared with untreated age-matched women, and concomitant use of a constant progestin (medroxyprogesterone acetate) may increase risk ratios into the 1.3-1.4 range [3, 4, 182]. An increase in breast cancer deaths has also been detected in long-term MHT users [14, 182]. Based on statistics from the NCI SEER database available on the internet [183], incidence of breast cancer in women 45-54 and 55-64 are 13.2 and 12.6 (average 12.9) new cases/10,000 women per year. Based on our 4 year estimate of 1,260 woman-years in the placebo group, as many as 2.0 new cases of breast cancer would be expected to occur spontaneously and (assuming a risk ratio of 1.3) 2.5 cases in each active treatment group, an excess due to estrogen treatment of 1 additional case during 5 years. This is probably an overestimate because:

a. No significant excess breast cancer risk was observed in the WHI E+P arm until after 5 years of study [47], and then only in women with a prior

history of estrogen use. The expected dropout rate indicates that approximately 68% of women taking active estrogen will complete 4 years of study. Presumably, relative risk will be less for women dropping out.

b. The risk ratio assumed is from the highest level observed in studies of combined estrogen and medroxyprogesterone acetate, was lower when estrogen was given alone [4] and no increase in breast cancer risk was seen in the E-only arm of the WHI trial [86]. Breast cancer risk is not known to be affected by intermittent natural progesterone.

To minimize risk, mammography and careful manual breast examination will be conducted regularly (before randomization, and then yearly). Women with a history of breast cancer or biopsy showing ductal carcinoma *in situ* (DCIS) will be excluded from study. Suspicious findings on mammography or manual examination will be followed up by appropriate biopsy. New findings of DCIS or significant atypia in biopsy samples will be cause for discontinuation of study medications.

5. Cholelithiasis/cholecystis – Cholelithiasis and cholecystitis- a small increase in the incidence of gallbladder disease has been described in women taking oral estrogens. Women will be cautioned to consult their physicians for symptoms of right upper quadrant pain, postprandial bloating, jaundice, or unexplained fever.

6. Endometrial cancer- In reports of 2-3 fold greater doses than are contemplated, estrogen use, unopposed by progestin has resulted in increased numbers of patients with endometrioid (low grade) [184] endometrial cancer with risk increasing with years of use. Maximum observed risk ratios have been on the order of 1.5-2.1 [185-187]. Observations from the WHI study show that menopausal women taking continuous estrogen/progestin are at no greater risk (HR = 0.81; 95% CI, 0.48-1.36) for endometrial cancer than untreated women [86]. In other studies, women taking cyclic estrogen/progestin at least 10 days per month were also at no greater risk of endometrial cancer than age-matched untreated women [188]. From a metaanalysis of 23 randomized controlled trials [189], it was concluded that women on cyclic estrogen, progestin therapy had no greater occurrence of endometrial hyperplasia than untreated women. No increase in endometrial cancer deaths has been observed in

combined estrogen-progestin users [190] or in unopposed estrogen at the doses to be used. The hormone regimen proposed results in regular withdrawal bleeding, and no excess risk of endometrial cancer is expected. To minimize risk, women will be screened before admission to the study by transvaginal ultrasound and excluded if endometrial thickness is >5 mm, unless follow-up endometrial biopsy shows no evidence of complex endometrial hyperplasia with or without atypia or of endometrial cancer. At 90 day intervals women will be monitored for vaginal bleeding as recorded on a 3 month daily bleeding diary (see attached). At any time during the 4 years of study women who have unscheduled bleeding, defined as 2 or more episodes of vaginal bleeding more than 7 days after progesterone withdrawal in any 12 month period, will undergo a Pipelle® aspiration endometrial biopsy. Women with a diagnosis of complex endometrial hyperplasia with atypia or endometrial cancer will instructed to discontinue study medications and be referred for appropriate care.

7. Breast swelling and tenderness- This is a common adverse effect of MHT use, occurring in 10-15% of women within days to a few weeks of initiation of treatment and tending to improve, despite continuation of treatment, with time (2-3 months).
8. Cognitive Disorders and Dementia - Most prior (epidemiological and observational) studies of MHT have shown significant protection against Alzheimer's dementia [73-79]. However, in the WHI study there was an increase in dementia in women taking MHT of the order of 2-fold vs. placebo, about 80% of which was classified as Alzheimer's type in both groups [69, 72]. In these reports, only patients over 65 years of age were studied. Given the reported excess of thromboembolic disease and thrombotic stroke in this population, we believe that the excess of dementia in the WHI study was probably due to occult small vascular occlusions which can produce dementia independently and also may accelerate Alzheimer's disease. It is unlikely that any excess of dementia will be observed in the younger women treated in the proposed study.
9. Psychiatric Symptoms – Depression, nervousness, somnolence, fatigue, and reduced libido have been reported in various studies of MHT. Women will be evaluated at 3 month intervals by questionnaire for these symptoms and if serious psychiatric

problems are detected will have their study medications placed on hold and be referred for further evaluation..

10. Vaginal bleeding- Withdrawal bleeding is expected for 3-6 days after each course of progesterone. Bleeding diaries will be evaluated every 3 months and women with bleeding at unexpected times or excessively heavy bleeding will be further evaluated as outlined above.
11. Headaches, especially migraine headaches- An increase in headaches and migraine headaches of the order of 15-20% has been reported in women taking MHT.
12. Peripheral edema- Swelling of feet or, rarely, hands occurs in small numbers (10-15%) of women taking MHT. This is mainly a "nuisance" side effect, which tends to improve with time on treatment.
13. Hypertension- Oral estrogens occasionally result in modest elevations of blood pressure due to enhanced hepatic production of angiotensinogen (renin substrate). Women will be monitored for elevations in blood pressure at 3-month intervals in the first year, and yearly thereafter. Hypertension will be treated appropriately (thiazide, ACE inhibitor, or angiotensin receptor blocker).
14. Continued menopausal symptoms- In the event of continued vasomotor instability symptoms (hot flashes, night sweats) the principal investigators or participant's primary care physician will be able to prescribe serotonin reuptake inhibitors (SSRI's), and for complaints of vaginal dryness or dsyparenuia estrogen-containing vaginal cream(s),

ii. Risks related to Study Procedures-

1. CIMT – There are no known risks of B-mode ultrasound determinations

Radiation Dose Considerations. A millirem is a unit of measurement of radiation. For the sake of comparison, estimated doses of typical medical and dental radiation procedures are: chest x-ray (25 mrem), dental x-rays (750 mrem), barium enema x-ray (2000 mrem). Non-medical doses are: natural radiation exposure living at sea level, 100 mrem per year and watching TV 1 hour per day, 1 mrem annually.

2. Coronary Calcium –will be determined by electron beam tomography (EBT) or multidetector tomography (MDT), depending on the study center: The total radiation dose based on 2 cardiac scans done sequentially at each sitting (two will done at

baseline and two again at 48 months) is shown below [191]. The radiation dose during two sets of scans is approximately 1.2 mSev (skin dose) for Electron Beam Computed tomography, 2.0 mSev for Siemens and General Electric Scanners used in this protocol. This will be applied to the thorax covering 12 cm in the z axis. Each EBCT examination adds the equivalent risk of one year of background ionizing radiation, each spiral CT adds the equivalent of three years of background ionizing radiation.

Table 5-

Study Total Radiation Doses from X-ray Tomography for Coronary Calcium

**Scanner**	**Skin Dose**	**Effective Dose**	Intrinsic Background Effective Dose/year
Imatron (EBT)	2520 mrem	2.4 mSv	2.3 – 3.0 mSv
Siemens (MDT)	3080 mrem	4.0 mSv	2.3 – 3.0 mSv
GE (MDT)	2562 mrem	4.0 mSv	2.3 – 3.0 mSv

3. Mammography- The radiation dose varies, depending on equipment employed and the thickness of breast tissue to be imaged. Using default values of peak voltage of 25.0 kilovolts with a filtration of 0.27 mm, in a compressed breast of 4.0 cm thickness and a glandular fraction of 0.50, dose is estimated at 143 mrem per roentgen. For a skin entrance exposure of 0.943 R, the total dose to glandular breast tissue is 135 mrem. Because it is standard of care for women in the age group to be studied to undergo yearly mammography, there is no excess risk to study subjects engendered by study participation.
4. DEXA scanning - The estimated dose of radiation from the DEXA machine is less than 25 mrem. Cumulative dose from four DEXA scans over the 4 year period of study is thus approximately 100 mrem.
5. Vaginal ultrasound- this procedure has no serious adverse risks. It is moderately invasive. Some (30-50%) women find it uncomfortable and rare women complain of pain exceeding simple discomfort. Pain is more likely in older women with estrogen deficiency leading to vaginal atrophy.
6. Endometrial aspiration biopsy- This procedure is invasive. Risks include allergy or

vaginal irritation from antiseptic solution used to sterilize the cervix, pain on entry into the cervix, syncope, perforation of the uterine wall, bleeding, and endometrial infection. Symptoms of weakness, sweating, dizziness, lightheadedness, and nausea may occur rarely during an endometrial biopsy. Bradycardia, possibly related to pain, has been reported. With older methodology perforation rates were as high as 4 per 1000 patients [192]. Perforation is thought to be less likely with a plastic cannula such as the Pipelle®. A small amount of vaginal bleeding is common for 1-3 days after biopsy, but serious hemorrhage is extremely rare. All complications are more common in postmenopausal women who have atrophy of the cervix and uterus. Complications will be minimized by training and oversight of operators by the gynecologist investigator, cleansing of the cervix with antiseptic solution (povidone iodine) before entry, and use of the Pipelle® pre-sterilized aspiration straw. The Pipelle® has an O.D. of 3.1mm and is readily inserted in most cases without the need for dilation. The Pipelle® curette is flexible to reduce risk of perforation and shaped to facilitate adaptation to normal uterine curvature thus promoting contact with the wall. Endometrial biopsy is standard of care in clinical practice for women with unscheduled (other than at expected menstrual intervals) vaginal bleeding.

c. Management of Adverse Events – Emergency care of acute adverse events suffered by subjects while participating in a study procedure will be managed by the study center hospital at the expense of the study center. If necessary the subject will be conveyed to the institution's emergency facility. This responsibility will extend to include costs of endometrial ultrasound and Pipelle® aspiration endometrial biopsy for subjects with abnormal vaginal bleeding. Neither the sponsor (KLRI) nor the study center will assume responsibility for management or care of adverse events whose risk is known or thought to be affected by use of MHT, such as deep vein thrombosis, pulmonary embolism, stroke, myocardial infarction, etc., unless they appear to be directly related to a study procedure.

Women with persistent, intolerable menopausal symptoms may be treated by their primary care physicians or the study center provider(s) with Effexor®, other SSRI medication, or other agents shown to be helpful for relief of these symptoms. Women who are started on effective estrogenic medications by their private physicians will be asked to discontinue their study medications and will continue to be followed.

d. Reporting of Serious Adverse Events- Subjects will be asked to report immediately all serious adverse events (AE’s) including, but not limited to: heart attack or chest pain resulting in hospitalization; pulmonary embolus, or thrombophlebitis; acute cholecystitis; new diagnosis of any cancer; cerebrovascular accident or TIA; severe nausea and vomiting (hyperemesis); migraine headaches; bone fractures unrelated to severe trauma; heavy and persistent (more than 3 days) vaginal bleeding; and onset of severe depression. Subjects will be requested to call a number at the local study center to leave a voice mail message for the local study coordinator. Center study coordinators or their deputies will check for AE messages at least every 24 hours, and contact the reporting subject to verify the AE report. All AE’s will be recorded in the central study database. The report form for AE's employs ICD-9 diagnosis codes in order to assure that the each event is always identified in common with other occurrences of the same type of event. Adverse events will be tabulated quarterly and reported to the DSMB (see below). At the end of the study, numbers of adverse events will be summarized and reported in a peer reviewed publication. All serious AE’s will be communicated by the study center PI (or his/her designate) by phone or email to the core (KLRI) study center. The core center (KLRI) study coordinator or her designate will notify the Data Safety Monitoring Board and also put out an email “medical alert” to the PI and the study coordinator at the other 7 study centers within 72 hours of the receiving a serious AE report.

e. Data Safety Monitoring Board - We have established a Data Safety Monitoring Board (DSMB) to oversee research subject safety during progress of the proposed study. Appropriate amounts of money have been budgeted to cover the costs associated with the operation of the DSMB. This committee will consist of five nationally recognized experts in: cardiology (David Herrington, M.D., Professor of Medicine, Wake Forest University, School of Medicine, Winston-Salem, North Carolina, *Chair*); cardiac imaging (Robert Detrano, M.D. Professor of Medicine and Cardiology, Harbor - University of Los Angeles Research and Education Institute, Los Angeles, CA); obstetrics and gynecology and reproductive endocrinology (Robert Rebar, M.D., Executive Director, American Society for Reproductive Medicine, Birmingham Alabama); family medicine and women’s health (Tamsen L. Bassford, M.D., Chair, Department Family and Community Medicine, University of Arizona College of Medicine, Tucson, AZ); and epidemiology and biostatistics (Kathryn Davis

Kennedy, Ph.D. Professor of Biostatistics University of Washington School of Public Health, Seattle, WA). In the planning phase of the study, the DSMB will establish guidelines and operating procedures for treatment of adverse effects and for discontinuing study medications in subjects reporting adverse events, as well as for terminating a study arm for excess adverse effects. At initiation of the study, the DSMB will assume the oversight of the study and will monitor the randomization and recruitment, the progress of the studies, compliance with the protocol, and subject safety. The Data Safety and Monitoring Board will have the authority to determine whether the trial should be terminated prematurely for safety reasons. A designated unblinded safety monitor at the each site will manage participants that develop significant adverse effects according to the guidelines, independent of the local investigators who will remain blinded.

f. Stop points – The DSMB, in collaboration with the study center principal investigators will establish pre-set stop points for the study, based on incidences of new cardiovascular events, stroke, pulmonary embolus, breast cancer, and death from all causes, and an index for combinations of the these. The stop points will be set to detect statistically significant increases in these adverse events above the rates expected from the risk calculations described above. If either treatment group exceeds the pre-established stop points, women will be notified and that study arm will be terminated.

Stop points (study medications permanently stopped) for individual study subjects will consist of occurrence of new diagnoses of cardiovascular events (myocardial infarction, physician-diagnosed angina, coronary revascularization), stroke or TIA), deep vein thrombophlebitis, pulmonary embolus, breast cancer, endometrial cancer, cholecystitis or gallstones. In the case of gallbladder disease study medications will be held until after the subject has had definitive treatment (cholescystectomy). In case one of the above diagnoses is uncertain, study medications will be held until subsequent testing defines the diagnosis. Study medications may be restarted if testing fails to confirm or eliminates the critical diagnosis. Finally diagnosis of certain other cancers (e.g. melanoma, meningioma) known or suspected to be estrogen- or progestin-sensitive, will be stop points.

Any subject leaving the study before 48 months for any reason will be asked to undergo an exit assessment, to consist of all procedures described for the 48 month study visit, including complete physical examination, CIMT, coronary calcium, blood draws for all

safety and study endpoints, DEXA, and cognitive, affective, and QOL assessments.

POTENTIAL PITFALLS

A perceived problem with the study design is the study size, which requires use of surrogate endpoints for CVD, rather than "hard" clinical endpoints. The study is not powered to detect differences between treatment groups in events. This means that any result, even one showing 50% or greater slowing of arterial wall thickening by CIMT and/or calcium deposition by computerized tomography in the treated vs. placebo groups, will have to be interpreted conservatively as consistent with, but not demonstrating, cardiovascular protection. Much larger randomized controlled trials would be required to verify and extend the findings in the proposed study, in order to confirm the hypothesis that there is a "window of opportunity" in the peri-menopause during which MHT is significantly cardioprotective. Whether such studies will ever be conducted, even in the event of positive findings in the proposed study, is unknown.

Another potential pitfall is the difficulty of truly blinding a study in which estrogen is given to women with menopausal symptoms, since those experiencing symptom relief will suspect that they are taking active drug and vice-versa. This is further complicated by the use of cyclic progestin, which will lead to withdrawal bleeding in most women, leading them to conclude that they are getting active estrogen. This pitfall is moderated by the fact that investigators conducting and evaluating primary and secondary endpoints will remain blinded to treatment group. Moreover the endpoints evaluated, with the exception of quality of life and cognitive outcomes should not be affected by the subject's impression regarding treatment.

Recruiting is another potential problem. Given the negative publicity attendant on publication of the WHI hormone trial data, many women now believe that MHT is too dangerous for use by women of any age or status. Although this conclusion is not justified by the data, it nonetheless may produce a barrier to recruitment of adequate numbers of subjects in the time-frame projected. The Kronos Longevity Research Institute in cooperation with our study centers will conduct a national public information/awareness campaign including a website, printed material, and press releases and interviews with the lay press to clarify the evidence regarding MHT in younger peri-menopausal women and raise awareness that this remains an open issue. No direct efforts to recruit for KEEPS will be made as part of this public information initiative.

Non-compliance and dropouts are also likely to be issues in the proposed study. Besides usual adverse effects of MHT such as nausea, edema, and breast tenderness, women in the

placebo group may be unwilling to endure continued manifestations of estrogen deficiency, such as vasomotor symptoms and dyspareunia. On the other hand, menopausal women on active therapy may be intolerant of continued regular vaginal withdrawal bleeding. To minimize these problems women will be carefully counseled regarding these issues before being randomized into the trial. Study coordinators will establish rapport with and encourage women who are experiencing problems to retain them in the study. Finally, we have allowed for higher drop-out rates in the placebo group (42%) and substantial drop-out rates in the treatment group (32%) which should allow us to achieve numbers of subjects completing that will allow for statistically significant results.

An events flow sheet is shown in Table 6. Visits at which research and safety procedures will be carried out are marked with an "x." A more detailed flow sheet showing all visits (including 3 month follow-ups) is attached as an appendix. The study is tentatively scheduled to begin (depending on receipt of IRB approvals) in autumn, 2005. It is anticipated that it will require approximately 2 years to recruit 90 subjects at each study center. Each subject will be studied for four years, therefore completion of study subject visits is anticipated for Spring 2012.

TIMELINE:

Table 6. Course of Events Flow Sheet

	Visit 0	Visit 1	Visit 2	Visit 3	Visit 5	Visit 7	Visit 9	Visit 13	Visit 17
	**screen**	**baseline**	**mo 3**	**mo 6**	**mo 12**	**mo 18**	**mo 24**	**mo 36**	**mo 48**
Event									
Screening	X								
Informed consent	X								
Medical history	X								
Complete PE	X								X
Interim H & P				X	X		X	X	
Safety labs	X								X
CIMT	X	X			X		X	X	XX
Coronary Ca	X								X
DEXA		X			X		X	X	X
Lipid Profile		X			X			X	X
Coagulation Profile		X			X			X	X
Inflammatory Markers		X			X			X	X
Hormone Levels		X			X			X	X
Cognitive Studies		X				X		X	X
Sleep Index		X		X		X		X	X

FACILITIES AVAILABLE

The proposed study will be conducted at eight academic medical institutions besides KLRI, each of which has dedicated space and is fully equipped and staffed for carrying out sophisticated patient-oriented research. Resources at each institution include clinical and office space, laboratories for blood sample preparation, freezers for sample storage, and clinical pathology laboratory, radiological, and ultrasound support for obtaining imaging endpoints and safety monitoring determinations. Several of these centers have NIH sponsored General Clinical Research Centers where the study visits will take place. The eight study centers and PI's are:

INSTITUTION	PRINCIPAL INVESTIGATOR
University of Utah School of Medicine<br>410 Chipeta Way, Room 167<br>Salt Lake City, UT 84108	Eliot Brinton, M.D.<br>Associate Professor of Medicine
University of California at San Francisco<br>2356 Sutter Street, 7th floor<br>San Francisco, CA 94115-0916	Marcelle Cedars, M.D.<br>Professor, Obstetrics and Gynecology
Harvard Medical School<br>Brigham and Women's Hospital<br>900 Commonwealth Avenue, 3d FL<br>Boston, MA 02215	JoAnn Manson, M.D.<br>Professor, Medicine<br>Chief of Preventive Medicine
Mayo Clinic and School of Medicine<br>200 First St. S.W.<br>Rochester, MN 55905	Virginia Miller, Ph.D.<br>Professor<br>Director, Office of Women's Health
Columbia University<br>College of Physicians and Surgeons<br>622 West 168th Street<br>New York, NY 10032	Rogerio Lobo, M.D.<br>Professor, Obstetrics and Gynecology
University of Washington School of Medicine<br>Research A-151, VA Puget Sound Sd HCS<br>9600 Veterans Drive SW (18C/127)<br>Tacoma, WA 98493	George R. Merriam, M.D.,<br>Professor, Medicine
Yale University College of Medicine<br>333 Cedar Street, 331 FMB<br>New Haven, CT 06520-8063	Hugh S. Taylor, M.D.<br>Assoc. Professor, Obstetrics and Gynecology

CONFIDENTIALITY

All hard copy records with personal identifying data (subject names, addresses, phone numbers etc.) will be kept as confidential files in locked file cabinets by the study coordinator at the participating clinical study centers. These data will be accessible only to the center PI and the study coordinator. All digital files containing personal identifying information will be protected by password access and will be stored behind a HIPAA-compliant firewall. Study results for the 8 centers will be stored in a central relational digital database, accessible via the web to password-authorized investigators. Study subjects will be identified in the central database only by a coded identification (study ID) number. No personal identifying information will be entered into the central database. It will be possible to identify individual study subjects only by comparing ID numbers to a confidentially maintained key file at the study center where that person is participating. No subject's name or other identifying data will be revealed in any study publication without prior written consent by the subject to do so.

COMPENSATION and CHARGES

Subjects will be compensated for time and travel at the rate of 25.00 for short safety visits, and 50.00 for baseline and for each long visit. Telephone visits will not be compensated. Subjects will be encouraged to maintain their own health insurance. There will be 8 visits at 50.00 each and 10 at 25.00 each, for a total of 650.00 in compensation for those subjects completing the study. Subjects will not be charged for specific study-related procedures, study drugs, or materials. However, subjects will be asked to obtain their routine annual mammograms, to be paid by their personal health insurance. In the event that a subject does not have, or loses, insurance coverage for mammography, this cost will be met by the study center.

REFERENCES

1. Col NF, Eckman MH, Karas RH, Pauker SG, Goldberg RJ, Ross EM, et al. Patient-specific decisions about hormone replacement therapy in postmenopausal women. JAMA. 1997;277(14):1140-7.
2. Manson JE, Martin KA. Clinical practice. Postmenopausal hormone-replacement therapy. N Engl J Med. 2001 Jul 5;345(1):34-40.
3. Bergkvist L, Adami H-O, Persson I, Hoover R, Schairer C. The risk of breast cancer after estrogen and estrogen-progestin replacement. N Engl J Med. 1989;321(5):293-7.
4. Schairer C, Lubin J, Troisi R, Sturgeon S, Brinton L, Hoover R. Menopausal estrogen and estrogen-progestin replacement therapy and breast cancer risk. JAMA. 2000;283(4):485-91.
5. Stampfer MJ, Colditz GA, Willett WC, Manson JE, Rosner B, Speizer FE, et al. Postmenopausal estrogen therapy and cardiovascular disease. Ten-year follow-up from the nurses' health study. N Engl J Med. 1991;325(11):756-62.
6. Grady D, Rubin SM, Petitti DB, Fox CS, others a. Hormone therapy to prevent disease and prolong life in postmenopausal women. Ann Int Med. 1992;117:1016-37.
7. Bush TL. Evidence for primary and secondary prevention of coronary artery disease in women taking oestrogen replacement therapy. Eur Heart J. 1996;17 Suppl D:9-14.
8. Thompson SG, Meade TW, Greenberg G. The use of hormonal replacement therapy and the risk of stroke and myocardial infarction in women. J Epidemiol Community Health. 1989 Jun;43(2):173-8.
9. Hemminki E, McPherson K. Impact of postmenopausal hormone therapy on cardiovascular events and cancer: pooled data from clinical trials. Bmj. 1997 Jul 19;315(7101):149-53.
10. Bush TL, Cowan LD, Barrett-Connor E, Criqui MH, Karon JM, Wallace RB, et al. Estrogen use and all-cause mortality. Preliminary results from the Lipid Research Clinics Program Follow-Up Study. JAMA. 1983;249(7):903-6.
11. Bush TL, Barrett-Connor E, Cowan LD, Criqui MH, Wallace RB, Suchindran CM, et al. Cardiovascular mortality and noncontraceptive use of estrogen in women: results from the Lipid Research Clinics Program Follow-up Study. Circulation. 1987 Jun;75(6):1102-9.
12. Henderson BE, Paganini-Hill A, Ross RK. Decreased mortality in users of estrogen replacement therapy. Arch Intern Med. 1991 Jan;151(1):75-8.
13. Ettinger B, Friedman GD, Bush T, Quesenberry CPJ. Reduced mortality associated with long-term postmenopausal estrogen therapy. Obstetrics and Gynecology. 1996;87:6-12.
14. Grodstein F, Stampfer MJ, Colditz GA, Willett WC, Manson JE, Joffe M, et al. Postmenopausal hormone therapy and mortality. N Engl J Med. 1997;336(25):1769-75.
15. Michaelsson K, Baron JA, Farahmand BY, Johnell O, Magnusson C, Persson PG, et al. Hormone replacement therapy and risk of hip fracture: population based case-control study. The Swedish Hip Fracture Study Group. Bmj. 1998 Jun 20;316(7148):1858-63.
16. Randell KM, Honkanen RJ, Kroger H, Saarikoski S. Does hormone-replacement therapy prevent fractures in early postmenopausal women? J Bone Miner Res. 2002 Mar;17(3):528-33.
17. Mosekilde L, Beck-Nielsen H, Sorensen OH, Nielsen SP, Charles P, Vestergaard P, et al. Hormonal replacement therapy reduces forearm fracture incidence in recent postmenopausal women - results of the Danish Osteoporosis Prevention Study. Maturitas.

2000 Oct 31;36(3):181-93.
18. Wolf PH, Madans JH, Finucane FF, Higgins M, Kleinman JC. Reduction of cardiovascular disease-related mortality among postmenopausal women who use hormones: evidence from a national cohort. Am J Obstet Gynecol. 1991 Feb;164(2):489-94.
19. Humphrey LL, Chan BK, Sox HC. Postmenopausal hormone replacement therapy and the primary prevention of cardiovascular disease. Ann Intern Med. 2002 Aug 20;137(4):273-84.
20. Colditz GA, Willett WC, Stampfer MJ, Rosner B, Speizer FE, Hennekens CH. Menopause and the risk of coronary heart disease in women. N Engl J Med. 1987 Apr 30;316(18):1105-10.
21. Kannel WB, Hjortland MC, McNamara PM, Gordon T. Menopause and risk of cardiovascular disease: the Framingham study. Ann Intern Med. 1976;85:447-52.
22. trial TWGftP. Effects of estrogen or estrogen/progestin regimens on heart disease risk factors in postmenopausal women. The Postmenopausal Estrogen/Progestin Interventions (PEPI) Trial. The Writing Group for the PEPI Trial. JAMA. 1995;273(3):199-208.
23. Spencer C, Crook D, Ross D, Cooper A, Whitehead M, Stevenson J. A randomised comparison of the effects of oral versus transdermal 17beta-oestradiol, each combined with sequential oral norethisterone acetate, on serum lipoprotein levels. Br J Obstet Gynaecol. 1999 Sep;106(9):948-53.
24. Futterman LG, Lemberg L. Lp(a) lipoprotein--an independent risk factor for coronary heart disease after menopause. Am J Crit Care. 2001 Jan;10(1):63-7.
25. Smolders RG, van der Mooren MJ, Teerlink T, Merkus JM, Kroeks MV, Franke HR, et al. A randomized placebo-controlled study of the effect of transdermal vs. oral estradiol with or without gestodene on homocysteine levels. Fertil Steril. 2003 Feb;79(2):261-7.
26. Rajkumar C, Kingwell BA, Cameron JD, Waddell T, Mehra R, Christophidis N, et al. Hormonal therapy increases arterial compliance in postmenopausal women. J Am Coll Cardiol. 1997 Aug;30(2):350-6.
27. Kawecka-Jaszcz K, Czarnecka D, Olszanecka A, Rajzer M, Jankowski P. The effect of hormone replacement therapy on arterial blood pressure and vascular compliance in postmenopausal women with arterial hypertension. J Hum Hypertens. 2002 Jul;16(7):509-16.
28. Moreau KL, Donato AJ, Seals DR, DeSouza CA, Tanaka H. Regular exercise, hormone replacement therapy and the age-related decline in carotid arterial compliance in healthy women. Cardiovasc Res. 2003 Mar;57(3):861-8.
29. McCubbin JA, Helfer SG, Switzer FS, 3rd, Price TM. Blood pressure control and hormone replacement therapy in postmenopausal women at risk for coronary heart disease. Am Heart J. 2002 Apr;143(4):711-7.
30. Angerer P, Stork S, von Schacky C. Influence of 17beta-oestradiol on blood pressure of postmenopausal women at high vascular risk. J Hypertens. 2001 Dec;19(12):2135-42.
31. Scuteri A, Bos AJ, Brant LJ, Talbot L, Lakatta EG, Fleg JL. Hormone replacement therapy and longitudinal changes in blood pressure in postmenopausal women. Ann Intern Med. 2001 Aug 21;135(4):229-38.
32. Stork S, von Schacky C, Angerer P. The effect of 17beta-estradiol on endothelial and inflammatory markers in postmenopausal women: a randomized, controlled trial. Atherosclerosis. 2002 Dec;165(2):301-7.
33. Guzic-Salobir B, Keber I, Seljeflot I, Arnesen H, Vrabic L. Combined hormone

replacement therapy improves endothelial function in healthy postmenopausal women. J Intern Med. 2001 Dec;250(6):508-15.
34. Seljeflot I, Arnesen H, Hofstad AE, Os I. Reduced expression of endothelial cell markers after long-term transdermal hormone replacement therapy in women with coronary artery disease. Thromb Haemost. 2000 Jun;83(6):944-8.
35. Telci A, Cakatay U, Akhan SE, Bilgin ME, Turfanda A, Sivas A. Postmenopausal hormone replacement therapy use decreases oxidative protein damage. Gynecol Obstet Invest. 2002;54(2):88-93.
36. Yen CH, Hsieh CC, Chou SY, Lau YT. 17Beta-estradiol inhibits oxidized low density lipoprotein-induced generation of reactive oxygen species in endothelial cells. Life Sci. 2001 Dec 14;70(4):403-13.
37. Bhavnani BR, Cecutti A, Gerulath A, Woolever AC, Berco M. Comparison of the antioxidant effects of equine estrogens, red wine components, vitamin E, and probucol on low-density lipoprotein oxidation in postmenopausal women. Menopause. 2001 Nov-Dec;8(6):408-19.
38. Arteaga E, Rojas A, Villaseca P, Bianchi M. The effect of 17beta-estradiol and alpha-tocopherol on the oxidation of LDL cholesterol from postmenopausal women and the minor effect of gamma-tocopherol and melatonin. Menopause. 2000 Mar-Apr;7(2):112-6.
39. Barrett-Connor E, Wenger NK, Grady D, Mosca L, Collins P, Kornitzer M, et al. Hormone and nonhormone therapy for the maintenance of postmenopausal health: the need for randomized controlled trials of estrogen and raloxifene. J Womens Health. 1998;7(7):839-47.
40. Nachtigall LE, Nachtigall RH, Nachtigall RD, Beckman EM. Estrogen replacement therapy II: a prospective study in the relationship to carcinoma and cardiovascular and metabolic problems. Obstet Gynecol. 1979 Jul;54(1):74-9.
41. Espeland MA, Applegate W, Furberg CD, Lefkowitz D, Rice L, Hunninghake D. Estrogen replacement therapy and progression of intimal-medial thickness in the carotid arteries of postmenopausal women. ACAPS Investigators. Asymptomatic Carotid Atherosclerosis Progression Study. Am J Epidemiol. 1995 Nov 15;142(10):1011-9.
42. Hodis HN, Mack WJ, Lobo RA, Shoupe D, Sevanian A, Mahrer PR, et al. Estrogen in the prevention of atherosclerosis. A randomized, double-blind, placebo-controlled trial. Ann Intern Med. 2001 Dec 4;135(11):939-53.
43. Akhrass F, Evans AT, Wang Y, Rich S, Kannan CR, Fogelfeld L, et al. Hormone replacement therapy is associated with less coronary atherosclerosis in postmenopausal women. J Clin Endocrinol Metab. 2003 Dec;88(12):5611-4.
44. Hulley S, Grady D, Bush T, Furberg C, Herrington D, Riggs B, et al. Randomized trial of estrogen plus progestin for secondary prevention of coronary heart disease in postmenopausal women. Heart and Estrogen/progestin Replacement Study (HERS) Research Group. JAMA. 1998;280(7):605-13.
45. Byington RP, Furberg CD, Herrington DM, Herd JA, Hunninghake D, Lowery M, et al. Effect of estrogen plus progestin on progression of carotid atherosclerosis in postmenopausal women with heart disease: HERS B-mode substudy. Arterioscler Thromb Vasc Biol. 2002 Oct 1;22(10):1692-7.
46. Herrington DM, Reboussin DM, Brosnihan KB, Sharp PC, Shumaker SA, Snyder TE, et al. Effects of estrogen replacement on the progression of coronary-artery atherosclerosis. N Engl J Med. 2000;343(8):522-9.

47. trials TWGftW. Risks and benefits of estrogen plus progestin in healthy postmenopausal women: principal results From the Women's Health Initiative randomized controlled trial. JAMA. 2002 Jul 17;288(3):321-33.
48. Lemay A. The relevance of the women's health initiative results on combined hormone replacement therapy in clinical practice. J Obstet Gynaecol Can. 2002 Sep;24(9):711-5.
49. De Lignieres B, Basdevant A, Thomas G, Thalabard JC, Mercier-Bodard C, Conard J, et al. Biological effects of estradiol-17 beta in postmenopausal women: oral versus percutaneous administration. J Clin Endocrinol Metab. 1986;62(3):536-41.
50. Scarabin PY, Alhenc-Gelas M, Plu-Bureau G, Taisne P, Agher R, Aiach M. Effects of oral and transdermal estrogen/progesterone regimens on blood coagulation and fibrinolysis in postmenopausal women. A randomized controlled trial. Arterioscler Thromb Vasc Biol. 1997 Nov;17(11):3071-8.
51. Alkjaersig N, Fletcher AP, de Ziegler D, Steingold KA, Meldrum DR, Judd HL. Blood coagulation in postmenopausal women given estrogen treatment: comparison of transdermal and oral administration. J Lab Clin Med. 1988 Feb;111(2):224-8.
52. Grodstein F, Manson JE, Colditz GA, Willett WC, Speizer FE, Stampfer MJ. A prospective, observational study of postmenopausal hormone therapy and primary prevention of cardiovascular disease. Ann Intern Med. 2000 Dec 19;133(12):933-41.
53. Pradhan AD, Manson JE, Rossouw JE, Siscovick DS, Mouton CP, Rifai N, et al. Inflammatory biomarkers, hormone replacement therapy, and incident coronary heart disease: prospective analysis from the Women's Health Initiative observational study. JAMA. 2002 Aug 28;288(8):980-7.
54. Manning PJ, Sutherland WH, Allum AR, de Jong SA, Jones SD. Effect of hormone replacement therapy on inflammation-sensitive proteins in post-menopausal women with Type 2 diabetes. Diabet Med. 2002 Oct;19(10):847-52.
55. Zegura B, Keber I, Sebestjen M, Koenig W. Double blind, randomized study of estradiol replacement therapy on markers of inflammation, coagulation and fibrinolysis. Atherosclerosis. 2003 May;168(1):123-9.
56. Wingrove CS, Garr E, Godsland IF, Stevenson JC. 17beta-oestradiol enhances release of matrix metalloproteinase-2 from human vascular smooth muscle cells. Biochim Biophys Acta. 1998 Mar 5;1406(2):169-74.
57. Zanger D, Yang BK, Ardans J, Waclawiw MA, Csako G, Wahl LM, et al. Divergent effects of hormone therapy on serum markers of inflammation in postmenopausal women with coronary artery disease on appropriate medical management. J Am Coll Cardiol. 2000;36:1797-802.
58. Lagrand WK, Visser CA, Hermens WT, Niessen HW, Verheugt FW, Wolbink GJ, et al. C-reactive protein as a cardiovascular risk factor: more than an epiphenomenon? Circulation. 1999 Jul 6;100(1):96-102.
59. Sakkinen P, Abbott RD, Curb JD, Rodriguez BL, Yano K, Tracy RP. C-reactive protein and myocardial infarction. J Clin Epidemiol. 2002 May;55(5):445-51.
60. Willerson JT. Systemic and local inflammation in patients with unstable atherosclerotic plaques. Prog Cardiovasc Dis. 2002 May-Jun;44(6):469-78.
61. Folsom AR, Pankow JS, Tracy RP, Arnett DK, Peacock JM, Hong Y, et al. Association of C-reactive protein with markers of prevalent atherosclerotic disease. Am J Cardiol. 2001 Jul 15;88(2):112-7.
62. Ikeda U, Shimada K. Matrix metalloproteinases and coronary artery diseases. Clin Cardiol.

2003 Feb;26(2):55-9.
63. Loftus IM, Naylor AR, Goodall S, Crowther M, Jones L, Bell PR, et al. Increased matrix metalloproteinase-9 activity in unstable carotid plaques. A potential role in acute plaque disruption. Stroke. 2000 Jan;31(1):40-7.
64. Adams MR, Register TC, Golden DL, Wagner JD, Williams JK. Medroxyprogesterone acetate antagonizes inhibitory effects of conjugated equine estrogens on coronary artery atherosclerosis. Arterioscler Thromb Vasc Biol. 1997 Jan;17(1):217-21.
65. Clarkson TB, Anthony MS, Jerome CP. Lack of effect of raloxifene on coronary artery atherosclerosis of postmenopausal monkeys. J Clin Endocrinol Metab. 1998 Mar;83(3):721-6.
66. Clarkson TB, Anthony MS, Morgan TM. Inhibition of postmenopausal atherosclerosis progression: a comparison of the effects of conjugated equine estrogens and soy phytoestrogens. J Clin Endocrinol Metab. 2001 Jan;86(1):41-7.
67. Williams JK, Anthony MS, Honore EK, Herrington DM, Morgan TM, Register TC, et al. Regression of atherosclerosis in female monkeys. Arterioscler Thromb Vasc Biol. 1995 Jul;15(7):827-36.
68. Wassertheil-Smoller S, Hendrix SL, Limacher M, Heiss G, Kooperberg C, Baird A, et al. Effect of estrogen plus progestin on stroke in postmenopausal women: the Women's Health Initiative: a randomized trial. JAMA. 2003 May 28;289(20):2673-84.
69. Shumaker SA, Legault C, Thal L, Wallace RB, Ockene JK, Hendrix SL, et al. Estrogen plus progestin and the incidence of dementia and mild cognitive impairment in postmenopausal women: the Women's Health Initiative Memory Study: a randomized controlled trial. JAMA. 2003 May 28;289(20):2651-62.
70. Kalaria RN, Harshbarger-Kelly M, Cohen DL, Premkumar DR. Molecular aspects of inflammatory and immune responses in Alzheimer's disease. Neurobiol Aging. 1996 Sep-Oct;17(5):687-93.
71. Jellinger KA. The pathology of ischemic-vascular dementia: an update. J Neurol Sci. 2002 Nov 15;203-204:153-7.
72. Rapp SR, Espeland MA, Shumaker SA, Henderson VW, Brunner RL, Manson JE, et al. Effect of estrogen plus progestin on global cognitive function in postmenopausal women: the Women's Health Initiative Memory Study: a randomized controlled trial. JAMA. 2003 May 28;289(20):2663-72.
73. Lerner A, Koss E, Debanne S, Rowland D, Smyth K, Friedland R. Smoking and oestrogen-replacement therapy as protective factors for Alzheimer's disease [letter]. Lancet. 1997;349(9049):403-4.
74. Waring SC, Rocca WA, Petersen RC, O'Brien PC, Tangalos EG, Kokmen E. Postmenopausal estrogen replacement therapy and risk of AD: a population-based study. Neurology. 1999;52(5):965-70.
75. Rice MM, Graves AB, McCurry SM, Gibbons LE, Bowen JD, McCormick WC, et al. Postmenopausal estrogen and estrogen-progestin use and 2-year rate of cognitive change in a cohort of older Japanese American women: The Kame Project. Arch Intern Med. 2000 Jun 12;160(11):1641-9.
76. Tang MX, Jacobs D, Stern Y, Marder K, Schofield P, Gurland B, et al. Effect of oestrogen during menopause on risk and age at onset of Alzheimer's. Lancet. 1996;348:429-32.
77. Kawas C, Resnick S, Morrison A, Brookmeyer R, Corrada M, Zonderman A, et al. A prospective study of estrogen replacement therapy and the risk of developing Alzheimer's

disease: the Baltimore Longitudinal Study of Aging. Neurology. 1997;48(6):1517-21.
78. Hogervorst E, Williams J, Budge M, Riedel W, Jolles J. The nature of the effect of female gonadal hormone replacement therapy on cognitive function in post-menopausal women: a meta-analysis. Neuroscience. 2000;101(3):485-512.
79. Yaffe K, Sawaya G, Lieberburg I, Grady D. Estrogen therapy in postmenopausal women: effects on cognitive function and dementia. JAMA. 1998;279(9):688-95.
80. Wise PM, Dubal DB, Wilson ME, Rau SW, Bottner M. Minireview: neuroprotective effects of estrogen-new insights into mechanisms of action. Endocrinology. 2001;142(3):969-73.
81. Wang PN, Liao SQ, Liu RS, Liu CY, Chao HT, Lu SR, et al. Effects of estrogen on cognition, mood, and cerebral blood flow in AD: a controlled study. Neurology. 2000;54(11):2061-6.
82. Mulnard RA, Cotman CW, Kawas C, van Dyck CH, Sano M, Doody R, et al. Estrogen replacement therapy for treatment of mild to moderate Alzheimer disease: a randomized controlled trial. Alzheimer's Disease Cooperative Study [In Process Citation]. JAMA. 2000;283(8):1007-15.
83. Resnick SM, Henderson VW. Hormone therapy and risk of Alzheimer disease: a critical time. JAMA. 2002 Nov 6;288(17):2170-2.
84. Zandi PP, Carlson MC, Plassman BL, Welsh-Bohmer KA, Mayer LS, Steffens DC, et al. Hormone replacement therapy and incidence of Alzheimer disease in older women: the Cache County Study. JAMA. 2002 Nov 6;288(17):2123-9.
85. Chesler EJ, Juraska JM. Acute administration of estrogen and progesterone impairs the acquisition of the spatial morris water maze in ovariectomized rats. Horm Behav. 2000 Dec;38(4):234-42.
86. Anderson GL, Limacher M, Assaf AR, Bassford T, Beresford SA, Black H, et al. Effects of conjugated equine estrogen in postmenopausal women with hysterectomy: the Women's Health Initiative randomized controlled trial. JAMA. 2004 Apr 14;291(14):1701-12.
87. Shumaker SA, Legault C, Kuller L, Rapp SR, Thal L, Lane DS, et al. Conjugated equine estrogens and incidence of probable dementia and mild cognitive impairment in postmenopausal women: Women's Health Initiative Memory Study. JAMA. 2004 Jun 23;291(24):2947-58.
88. Espeland MA, Rapp SR, Shumaker SA, Brunner R, Manson JE, Sherwin BB, et al. Conjugated equine estrogens and global cognitive function in postmenopausal women: Women's Health Initiative Memory Study. JAMA. 2004 Jun 23;291(24):2959-68.
89. Hu FB, Stampfer MJ, Manson JE, Grodstein F, Colditz GA, Speizer FE, et al. Trends in the incidence of coronary heart disease and changes in diet and lifestyle in women. N Engl J Med. 2000 Aug 24;343(8):530-7.
90. Raggi P, Callister TQ, Cooil B, He ZX, Lippolis NJ, Russo DJ, et al. Identification of patients at increased risk of first unheralded acute myocardial infarction by electron-beam computed tomography. Circulation. 2000 Feb 29;101(8):850-5.
91. Manson JE, Hsia J, K.C. J, Rossouw JE, Assaf AR, Lasser NL, et al. Estrogen plus Progestin and the Risk of Coronary Heart Disease. N Engl J Med. 2003;349:523-34.
92. Chetkowski RJ, Meldrum DR, Steingold KA, Randle D, Lu JK, Eggena P, et al. Biologic effects of transdermal estradiol. N Engl J Med. 1986 Jun 19;314(25):1615-20.
93. de Lignieres B. Oral micronized progesterone. Clin Ther. 1999 Jan;21(1):41-60; discussion 1-2.

94. Expert Panel on Detection E, and Treatment of High Blood Cholesterol in Adults. (Adult Treatment Panel III). Executive Summary of the Third Report of the National Cholesterol Education Program (NCEP) Expert Panel on Detection, Evaluation, and Treatment of High Blood Cholesterol in Adults. JAMA. 2001;285:2486–97.
95. Grundy SM, Cleeman JI, Bairey Merz CN, Brewer HBJ, Clark LT, Hunninghake DB, et al. Implications of Recent Clinical Trials for the National Cholesterol Education Program Adult Treatment Panel III Guidelines. Circulation. 2004;110:227-39.
96. Blankenhorn DH, Selzer RH, Crawford DW, Barth JD, Liu CR, Liu CH, et al. Beneficial effects of colestipol-niacin therapy on the common carotid artery. Two- and four-year reduction of intima-media thickness measured by ultrasound. Circulation. 1993 Jul;88(1):20-8.
97. Mack WJ, Selzer RH, Hodis HN, Erickson JK, Liu CR, Liu CH, et al. One-year reduction and longitudinal analysis of carotid intima-media thickness associated with colestipol/niacin therapy. Stroke. 1993 Dec;24(12):1779-83.
98. Hodis HN, Mack WJ, LaBree L, Selzer RH, Liu C, Alaupovic P, et al. Reduction in carotid arterial wall thickness using lovastatin and dietary therapy: a randomized controlled clinical trial. Ann Intern Med. 1996 Mar 15;124(6):548-56.
99. Selzer RH, Hodis HN, Kwong-Fu H, Mack WJ, Lee PL, Liu CR, et al. Evaluation of computerized edge tracking for quantifying intima-media thickness of the common carotid artery from B-mode ultrasound images. Atherosclerosis. 1994 Nov;111(1):1-11.
100. Persson J, Formgren J, Israelsson B, Berglund G. Ultrasound-determined intima-media thickness and atherosclerosis. Direct and indirect validation. Arterioscler Thromb. 1994 Feb;14(2):261-4.
101. Pignoli P, Tremoli E, Poli A, Oreste P, Paoletti R. Intimal plus medial thickness of the arterial wall: a direct measurement with ultrasound imaging. Circulation. 1986 Dec;74(6):1399-406.
102. Sternby NH. Atherosclerosis in a defined population. An autopsy survey in Malmo, Sweden. Acta Pathol Microbiol Scand. 1968:Suppl 194:5+.
103. Wofford JL, Kahl FR, Howard GR, McKinney WM, Toole JF, Crouse JR, 3rd. Relation of extent of extracranial carotid artery atherosclerosis as measured by B-mode ultrasound to the extent of coronary atherosclerosis. Arterioscler Thromb. 1991 Nov-Dec;11(6):1786-94.
104. Craven TE, Ryu JE, Espeland MA, Kahl FR, McKinney WM, Toole JF, et al. Evaluation of the associations between carotid artery atherosclerosis and coronary artery stenosis. A case-control study. Circulation. 1990 Oct;82(4):1230-42.
105. Crouse JR, Toole JF, McKinney WM, Dignan MB, Howard G, Kahl FR, et al. Risk factors for extracranial carotid artery atherosclerosis. Stroke. 1987 Nov-Dec;18(6):990-6.
106. O'Leary DH, Polak JF, Kronmal RA, Kittner SJ, Bond MG, Wolfson SK, Jr., et al. Distribution and correlates of sonographically detected carotid artery disease in the Cardiovascular Health Study. The CHS Collaborative Research Group. Stroke. 1992 Dec;23(12):1752-60.
107. Furberg CD, Adams HP, Jr., Applegate WB, Byington RP, Espeland MA, Hartwell T, et al. Effect of lovastatin on early carotid atherosclerosis and cardiovascular events. Asymptomatic Carotid Artery Progression Study (ACAPS) Research Group. Circulation. 1994 Oct;90(4):1679-87.
108. Salonen R, Nyyssonen K, Porkkala E, Rummukainen J, Belder R, Park JS, et al. Kuopio Atherosclerosis Prevention Study (KAPS). A population-based primary preventive trial of

the effect of LDL lowering on atherosclerotic progression in carotid and femoral arteries. Circulation. 1995 Oct 1;92(7):1758-64.

109. Mercuri M, Bond MG, Sirtori CR, Veglia F, Crepaldi G, Feruglio FS, et al. Pravastatin reduces carotid intima-media thickness progression in an asymptomatic hypercholesterolemic mediterranean population: the Carotid Atherosclerosis Italian Ultrasound Study. Am J Med. 1996 Dec;101(6):627-34.
110. Shepherd J, Cobbe SM, Ford I, Isles CG, Lorimer AR, MacFarlane PW, et al. Prevention of coronary heart disease with pravastatin in men with hypercholesterolemia. West of Scotland Coronary Prevention Study Group. N Engl J Med. 1995 Nov 16;333(20):1301-7.
111. Downs JR, Clearfield M, Weis S, Whitney E, Shapiro DR, Beere PA, et al. Primary prevention of acute coronary events with lovastatin in men and women with average cholesterol levels: results of AFCAPS/TexCAPS. Air Force/Texas Coronary Atherosclerosis Prevention Study. JAMA. 1998 May 27;279(20):1615-22.
112. Crouse JR, 3rd, Byington RP, Bond MG, Espeland MA, Craven TE, Sprinkle JW, et al. Pravastatin, Lipids, and Atherosclerosis in the Carotid Arteries (PLAC-II). Am J Cardiol. 1995 Mar 1;75(7):455-9.
113. Ridker PM, Rifai N, Pfeffer MA, Sacks F, Braunwald E. Long-term effects of pravastatin on plasma concentration of C-reactive protein. The Cholesterol and Recurrent Events (CARE) Investigators. Circulation. 1999 Jul 20;100(3):230-5.
114. Prevention of cardiovascular events and death with pravastatin in patients with coronary heart disease and a broad range of initial cholesterol levels. The Long-Term Intervention with Pravastatin in Ischaemic Disease (LIPID) Study Group. N Engl J Med. 1998 Nov 5;339(19):1349-57.
115. Mack WJ, LaBree L, Liu C, Selzer RH, Hodis HN. Correlations between measures of atherosclerosis change using carotid ultrasonography and coronary angiography. Atherosclerosis. 2000 Jun;150(2):371-9.
116. Hodis HN, Mack WJ, LaBree L, Selzer RH, Liu CR, Liu CH, et al. The role of carotid arterial intima-media thickness in predicting clinical coronary events. Ann Intern Med. 1998 Feb 15;128(4):262-9.
117. Salonen JT, Salonen R. Ultrasound B-mode imaging in observational studies of atherosclerotic progression. Circulation. 1993 Mar;87(3 Suppl):II56-65.
118. Chambless LE, Heiss G, Folsom AR, Rosamond W, Szklo M, Sharrett AR, et al. Association of coronary heart disease incidence with carotid arterial wall thickness and major risk factors: the Atherosclerosis Risk in Communities (ARIC) Study, 1987-1993. Am J Epidemiol. 1997 Sep 15;146(6):483-94.
119. Bots ML, Hoes AW, Koudstaal PJ, Hofman A, Grobbee DE. Common carotid intima-media thickness and risk of stroke and myocardial infarction: the Rotterdam Study. Circulation. 1997 Sep 2;96(5):1432-7.
120. O'Leary DH, Polak JF, Kronmal RA, Manolio TA, Burke GL, Wolfson SK, Jr. Carotid-artery intima and media thickness as a risk factor for myocardial infarction and stroke in older adults. Cardiovascular Health Study Collaborative Research Group. N Engl J Med. 1999 Jan 7;340(1):14-22.
121. Blankenhorn DH, Hodis HN. George Lyman Duff Memorial Lecture. Arterial imaging and atherosclerosis reversal. Arterioscler Thromb. 1994 Feb;14(2):177-92.
122. Beach KW, Isaac CA, Phillips DJ, Strandness DE, Jr. An ultrasonic measurement of superficial femoral artery wall thickness. Ultrasound Med Biol. 1989;15(8):723-8.

123. O'Leary DH, Bryan FA, Goodison MW, Rifkin MD, Gramiak R, Ball M, et al. Measurement variability of carotid atherosclerosis: real-time (B-mode) ultrasonography and angiography. Stroke. 1987 Nov-Dec;18(6):1011-7.
124. Wendelhag I, Gustavsson T, Suurkula M, Berglund G, Wikstrand J. Ultrasound measurement of wall thickness in the carotid artery: fundamental principles and description of a computerized analysing system. Clin Physiol. 1991 Nov;11(6):565-77.
125. Mautner SL, Mautner GC, Froehlich J, Feuerstein IM, Proschan MA, Roberts WC, et al. Coronary artery disease: prediction with in vitro electron beam CT. Radiology. 1994 Sep;192(3):625-30.
126. Mautner GC, Mautner SL, Froehlich J, Feuerstein IM, Proschan MA, Roberts WC, et al. Coronary artery calcification: assessment with electron beam CT and histomorphometric correlation. Radiology. 1994 Sep;192(3):619-23.
127. Tanenbaum SR, Kondos GT, Veselik KE, Prendergast MR, Brundage BH, Chomka EV. Detection of calcific deposits in coronary arteries by ultrafast computed tomography and correlation with angiography. Am J Cardiol. 1989 Apr 1;63(12):870-2.
128. Rumberger JA, Sheedy PF, 2nd, Breen JF, Fitzpatrick LA, Schwartz RS. Electron beam computed tomography and coronary artery disease: scanning for coronary artery calcification. Mayo Clin Proc. 1996 Apr;71(4):369-77.
129. Budoff MJ, Georgiou D, Brody A, Agatston AS, Kennedy J, Wolfkiel C, et al. Ultrafast computed tomography as a diagnostic modality in the detection of coronary artery disease: a multicenter study. Circulation. 1996;93(5):898-904.
130. Kennedy J, Shavelle R, Wang S, Budoff M, Detrano RC. Coronary calcium and standard risk factors in symptomatic patients referred for coronary angiography. Am Heart J. 1998 Apr;135(4):696-702.
131. Simons DB, Schwartz RS, Edwards WD, Sheedy PF, Breen JF, Rumberger JA. Noninvasive definition of anatomic coronary artery disease by ultrafast computed tomographic scanning: a quantitative pathologic comparison study. J Am Coll Cardiol. 1992 Nov 1;20(5):1118-26.
132. Rumberger JA, Behrenbeck T, Breen JF, Sheedy PF, 2nd. Coronary calcification by electron beam computed tomography and obstructive coronary artery disease: a model for costs and effectiveness of diagnosis as compared with conventional cardiac testing methods. J Am Coll Cardiol. 1999 Feb;33(2):453-62.
133. Brown BG, Morse J, Zhao XQ, Cheung M, Marino E, Albers JJ. Electron-beam tomography coronary calcium scores are superior to Framingham risk variables for predicting the measured proximal stenosis burden. Am J Cardiol. 2001 Jul 19;88(2A):23E-6E.
134. Cheng YJ, Church TS, Kimball TE, Nichaman MZ, Levine BD, McGuire DK, et al. Comparison of coronary artery calcium detected by electron beam tomography in patients with to those without symptomatic coronary heart disease. Am J Cardiol. 2003 Sep 1;92(5):498-503.
135. Callister TQ, Cooil B, Raya SP, Lippolis NJ, Russo DJ, Raggi P. Coronary artery disease: improved reproducibility of calcium scoring with an electron-beam CT volumetric method. Radiology. 1998 Sep;208(3):807-14.
136. Callister TQ, Raggi P, Cooil B, Lippolis NJ, Russo DJ. Effect of HMG-CoA reductase inhibitors on coronary artery disease as assessed by electron-beam computed tomography. N Engl J Med. 1998 Dec 31;339(27):1972-8.

137. Budoff MJ, Lane KL, Bakhsheshi H, Mao S, Grassmann BO, Friedman BC, et al. Rates of progression of coronary calcium by electron beam tomography. Am J Cardiol. 2000 Jul 1;86(1):8-11.
138. Maher JE, Raz JA, Bielak LF, Sheedy PFn, Schwartz RS, Peyser PA. Potential of quantity of coronary artery calcification to identify new risk factors for asymptomatic atherosclerosis. Am J Epidemiol. 1996;144:943-53.
139. Budoff MJ, Raggi P. Coronary artery disease progression assessed by electron-beam computed tomography. Am J Cardiol. 2001 Jul 19;88(2A):46E-50E.
140. Third Report of the National Cholesterol Education Program (NCEP) Expert Panel on Detection, Evaluation, and Treatment of High Blood Cholesterol in Adults (Adult Treatment Panel III) final report. Circulation. 2002 Dec 17;106(25):3143-421.
141. Ginsberg HN. Is hypertriglyceridemia a risk factor for atherosclerotic cardiovascular disease? A simple question with a complicated answer. Ann Intern Med. 1997 Jun 1;126(11):912-4.
142. Knopp RH. Drug treatment of lipid disorders. N Engl J Med. 1999 Aug 12;341(7):498-511.
143. Miller VT, Muesing RA, LaRosa JC, Stoy DB, Phillips EA, Stillman RJ. Effects of conjugated equine estrogen with and without three different progestogens on lipoproteins, high-density lipoprotein subfractions, and apolipoprotein A-I. Obstet Gynecol. 1991 Feb;77(2):235-40.
144. Slowinska-Srzednicka J, Zgliczynski S, Chotkowska E, Srzednicki M, Stopinska-Gluszak U, Jeske W, et al. Effects of transdermal 17 beta-oestradiol combined with oral progestogen on lipids and lipoproteins in hypercholesterolaemic postmenopausal women. J Intern Med. 1993 Nov;234(5):447-51.
145. Tikkanen MJ. Estrogens, progestins and lipid metabolism. Maturitas. 1996 May;23 Suppl:S51-5.
146. Paganini-Hill A, Dworsky R, Krauss RM. Hormone replacement therapy, hormone levels, and lipoprotein cholesterol concentrations in elderly women. Am J Obstet Gynecol. 1996 Mar;174(3):897-902.
147. Coresh J, Kwiterovich PO, Jr. Small, dense low-density lipoprotein particles and coronary heart disease risk: A clear association with uncertain implications. JAMA. 1996 Sep 18;276(11):914-5.
148. Barnes JF, Farish E, Rankin M, Hart DM. A comparison of the effects of two continuous HRT regimens on cardiovascular risk factors. Atherosclerosis. 2002 Jan;160(1):185-93.
149. Farish E, Spowart K, Barnes JF, Fletcher CD, Calder A, Brown A, et al. Effects of postmenopausal hormone replacement therapy on lipoproteins including lipoprotein(a) and LDL subfractions. Atherosclerosis. 1996 Sep 27;126(1):77-84.
150. Lilley SH, Spivey JM, Vadlamudi S, Otvos J, Cummings DM, Barakat H. Lipid and lipoprotein responses to oral combined hormone replacement therapy in normolipemic obese women with controlled type 2 diabetes mellitus. J Clin Pharmacol. 1998 Dec;38(12):1107-15.
151. Shlipak MG, Simon JA, Vittinghoff E, Lin F, Barrett-Connor E, Knopp RH, et al. Estrogen and progestin, lipoprotein(a), and the risk of recurrent coronary heart disease events after menopause. JAMA. 2000 Apr 12;283(14):1845-52.
152. Kulkarni KR, Garber DW, Marcovina SM, Segrest JP. Quantification of cholesterol in all lipoprotein classes by the VAP-II method. J Lipid Res. 1994;35:159-68.
153. Acs N, Vajo Z, Miklos Z, Siklosi G, Paulin F, Felicetta JV, et al. The effects of

postmenopausal hormone replacement therapy on hemostatic variables: a meta-analysis of 46 studies. Gynecol Endocrinol. 2002 Aug;16(4):335-46.
154. Gottsater A, Rendell M, Hulthen UL, Berntorp E, Mattiasson I. Hormone replacement therapy in healthy postmenopausal women: a randomized, placebo-controlled study of effects on coagulation and fibrinolytic factors. J Intern Med. 2001 Mar;249(3):237-46.
155. Boschetti C, Cortellaro M, Nencioni T, Bertolli V, Della Volpe A, Zanussi C. Short- and long-term effects of hormone replacement therapy (transdermal estradiol vs oral conjugated equine estrogens, combined with medroxyprogesterone acetate) on blood coagulation factors in postmenopausal women. Thromb Res. 1991 Apr;62(1-2):1-8.
156. Ridker PM, Genest J, Libby P. Risk factors for atherosclerotic disease. In: Braunwald E, Zipes DP, Libby P, eds. Heart Disease: A Textbook of Cardiovascular Medicine. Philadelphia: W.B. Saunders 2001:1010-39.
157. Tracy RP. Emerging relationships of inflammation, cardiovascular disease and chronic diseases of aging. Int J Obesity. 2003;27:S29–S34.
158. Cushman M, Legault C, Barrett-Connor E, Stefanick ML, Kessler C, Judd HL, et al. Effect of postmenopausal hormones on inflammation-sensitive proteins: the Postmenopausal Estrogen/Progestin Interventions (PEPI) Study. Circulation. 1999 Aug 17;100(7):717-22.
159. Hansen MA, Hassager C, Overgaard K, Marslew U, Riis BJ, Christiansen C. Dual-energy x-ray absorptiometry: a precise method of measuring bone mineral density in the lumbar spine. J Nucl Med. 1990 Jul;31(7):1156-62.
160. Bone densitometry as a screening tool for osteoporosis in postmenopausal women. Radiol Manage. 1998 Mar-Apr;20(2):43-54.
161. Mazess RB, Barden HS, Bisek JP, Hanson J. Dual-energy x-ray absorptiometry for total-body and regional bone-mineral and soft-tissue composition. Am J Clin Nutr. 1990 Jun;51(6):1106-12.
162. Ettinger B, Genant HK, Cann CE. Long-term estrogen replacement therapy prevents bone loss and fractures. Annals of Internal Medicine. 1985;102:319-24.
163. Felson DT, Zhang Y, Hannan MT, Kiel DP, Wilson PWF, Anderson JJ. The effect of postmenopausal estrogen therapy on bone density in elderly women. N Engl J Med. 1993;329:1141-6.
164. Gass M, Liu J, Rebar RW. The effect of low-dose conjugated equine estrogens and cyclic MPA on bone density. Maturitas. 2002 Feb 26;41(2):143-7.
165. Cauley JA, Robbins J, Chen Z, Cummings SR, Jackson RD, LaCroix AZ, et al. Effects of estrogen plus progestin on risk of fracture and bone mineral density: the Women's Health Initiative randomized trial. JAMA. 2003 Oct 1;290(13):1729-38.
166. Heymsfield SB, Smith R, Aulet M, Bensen B, Lichtman S, Wang J, et al. Appendicular skeletal muscle mass: measurement by dual-photon absorptiometry. Am J Clin Nutr. 1990 Aug;52(2):214-8.
167. Wang ZM, Pierson RN, Jr., Heymsfield SB. The five-level model: a new approach to organizing body-composition research. Am J Clin Nutr. 1992 Jul;56(1):19-28.
168. Kohrt WM, Malley MT, Dalsky GP, Holloszy JO. Body composition of healthy sedentary and trained, young and older men and women. Med Sci Sports Exerc. 1992 Jul;24(7):832-7.
169. Asthana S, Craft S, Baker LD, Raskind MA, Birnbaum RS, Lofgreen CP, et al. Cognitive and neuroendocrine response to transdermal estrogen in postmenopausal women with Alzheimer's disease: results of a placebo-controlled, double-blind, pilot study.

Psychoneuroendocrinology. 1999 Aug;24(6):657-77.
170. Asthana S, Baker LD, Craft S, Stanczyk FZ, Veith RC, Raskind MA, et al. High-dose estradiol improves cognition for women with AD: results of a randomized study. Neurology. 2001 Aug 28;57(4):605-12.
171. Utian WH, Janata JW, Kingsberg SA, Schluchter M, Hamilton JC. The Utian Quality of Life (UQOL) Scale: development and validation of an instrument to quantify quality of life through and beyond menopause. Menopause. 2002 Nov-Dec;9(6):402-10.
172. Rosen R, Brown C, Heiman J, Leiblum S, Meston C, Shabsigh R, et al. The Female Sexual Function Index (FSFI): a multidimensional self-report instrument for the assessment of female sexual function. J Sex Marital Ther. 2000 Apr-Jun;26(2):191-208.
173. MacMahon S, Sharpe N, Gamble G, Hart H, Scott J, Simes J, et al. Effects of lowering average of below-average cholesterol levels on the progression of carotid atherosclerosis: results of the LIPID Atherosclerosis Substudy. LIPID Trial Research Group. Circulation. 1998 May 12;97(18):1784-90.
174. McCulloch CE, Searle SR. Generalized, linear, and mixed models. New York: Wiley 2001.
175. Lobo RA. Evaluation of cardiovascular event rates with hormone therapy in healthy, early postmenopausal women: results from 2 large clinical trials. Arch Intern Med. 2004 Mar 8;164(5):482-4.
176. Broderick JP, Phillips SJ, Whisnant JP, O'Fallon WM, Bergstralh EJ. Incidence rates of stroke in the eighties: the end of the decline in stroke? Stroke. 1989 May;20(5):577-82.
177. Broderick J, Brott T, Kothari R, Miller R, Khoury J, Pancioli A, et al. The Greater Cincinnati/Northern Kentucky Stroke Study: preliminary first-ever and total incidence rates of stroke among blacks. Stroke. 1998 Feb;29(2):415-21.
178. Silverstein MD, Heit JA, Mohr DN, Petterson TM, O'Fallon WM, Melton LJ, 3rd. Trends in the incidence of deep vein thrombosis and pulmonary embolism: a 25-year population-based study. Arch Intern Med. 1998 Mar 23;158(6):585-93.
179. Heit JA, Melton LJ, 3rd, Lohse CM, Petterson TM, Silverstein MD, Mohr DN, et al. Incidence of venous thromboembolism in hospitalized patients vs community residents. Mayo Clin Proc. 2001 Nov;76(11):1102-10.
180. Cushman M, Kuller LH, Prentice R, Rodabough RJ, Psaty BM, Stafford RS, et al. Estrogen plus progestin and risk of venous thrombosis. JAMA. 2004 Oct 6;292(13):1573-80.
181. Straczek C, Oger E, Yon de Jonage-Canonico MB, Plu-Bureau G, Conard J, Meyer G, et al. Prothrombotic mutations, hormone therapy, and venous thromboembolism among postmenopausal women: impact of the route of estrogen administration. Circulation. 2005 Nov 29;112(22):3495-500.
182. Colditz GA, Hankinson SE, Hunter DJ, Willett WC, Manson JE, Stampfer MJ, et al. The use of estrogens and progestins and the risk of breast cancer in postmenopausal women. N Engl J Med. 1995 Jun 15;332(24):1589-93.
183. SEER. SEER Cancer Statistics Review, 1975-2000. [World Wide Web] 2000 [cited 2003; Available from: http://seer.cancer.gov/csr/1975_2000/
184. Horwitz RI, Feinstein AR, Vidone RA, Sommers SC, Robboy SJ. Histopathologic distinctions in the relationship of estrogens and endometrial cancer. JAMA. 1981 Sep 25;246(13):1425-7.
185. Smith DC, Prentice R, Thompson DJ, Herrmann WL. Association of exogenous estrogen and endometrial carcinoma. N Engl J Med. 1975 Dec 4;293(23):1164-7.
186. Mack TM, Pike MC, Henderson BE, Pfeffer RI, Gerkins VR, Arthur M, et al. Estrogens

and endometrial cancer in a retirement community. N Engl J Med. 1976 Jun 3;294(23):1262-7.

187. Weiss NS, Szekely DR, Austin DF. Increasing incidence of endometrial cancer in the United States. N Engl J Med. 1976 Jun 3;294(23):1259-62.

188. Newcomb PA, Trentham-Dietz A. Patterns of postmenopausal progestin use with estrogen in relation to endometrial cancer (United States). Cancer Causes Control. 2003 Mar;14(2):195-201.

189. Lethaby A, Farquhar C, Sarkis A, Roberts H, Jepson R, Barlow D. Hormone replacement therapy in postmenopausal women: endometrial hyperplasia and irregular bleeding. Cochrane Database Syst Rev. 2000(2):CD000402.

190. Schairer C, Adami HO, Hoover R, Persson I. Cause-specific mortality in women receiving hormone replacement therapy. Epidemiology. 1997 Jan;8(1):59-65.

191. Hidajat N, Wolf M, Rademaker J, Knowlmann F, Oestmann JW, Felix R. Radiation Dose in CT of the Heart for Coronary Heart Disease and CT of the Lung for Pulmonary Embolism: Comparisons between single-slice Detector CT, Multi-Slice Detector CT and EBT. Radiology. 2000;374:217.

192. Grimes DA. Diagnostic dilation and curettage: a reappraisal. Am J Obstet Gynecol. 1982;142:1-6.
