# Supplementary material for: Effects of Hormone Therapy on Cognition and Mood in Recently Postmenopausal Women: Findings from the Randomized, Controlled KEEPS–Cognitive and Affective Study
Source: PLoS Med. 2015 Jun 2;12(6):e1001833. doi: 10.1371/journal.pmed.1001833 (PMC4452757; doi:10.1371/journal.pmed.1001833)
Supplement: S2 Text — (DOC) [file pmed.1001833.s005.doc]

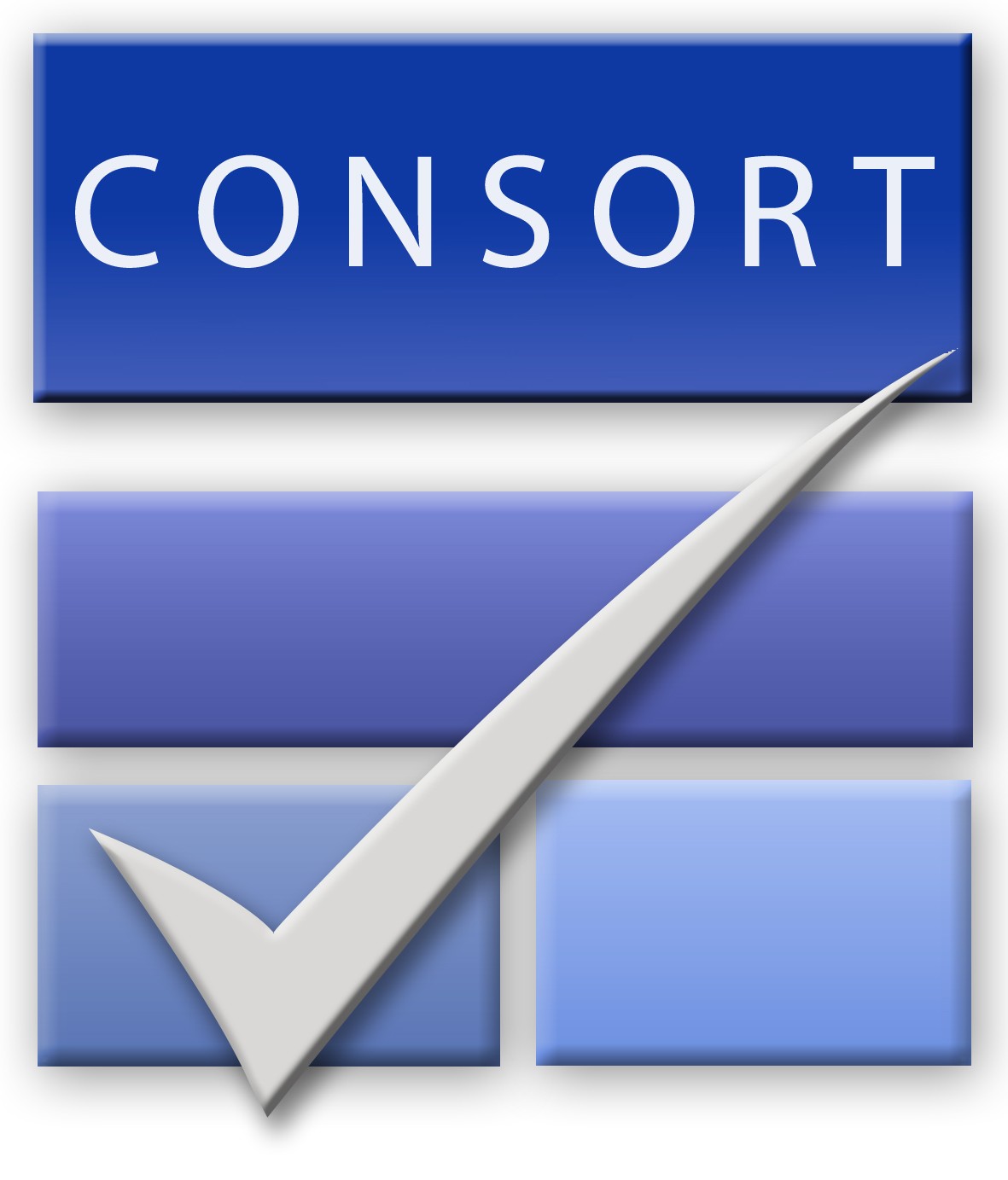
S2 Text: CONSORT 2010 checklist of information to include when reporting a randomised trial*

| Section/Topic | Item No | Checklist item | Reported on page No |
| --- | --- | --- | --- |
| Title and abstract | | | |
|  | 1a | Identification as a randomised trial in the title | Page 1 |
|  | 1b | Structured summary of trial design, methods, results, and conclusions (for specific guidance see CONSORT for abstracts) | Pages 5-6 |
| Introduction | | | |
| Background and objectives | 2a | Scientific background and explanation of rationale | Pages 7-8 |
|  | 2b | Specific objectives or hypotheses | Page 8 |
| Methods | | | |
| Trial design | 3a | Description of trial design (such as parallel, factorial) including allocation ratio | Page 9 |
|  | 3b | Important changes to methods after trial commencement (such as eligibility criteria), with reasons | Page 10 (MMSE cut off) |
| Participants | 4a | Eligibility criteria for participants | Pages 9 to 10 |
|  | 4b | Settings and locations where the data were collected | Pages 9 to 10: Page 30 |
| Interventions | 5 | The interventions for each group with sufficient details to allow replication, including how and when they were actually administered | Pages 10 to 11 |
| Outcomes | 6a | Completely defined pre-specified primary and secondary outcome measures, including how and when they were assessed | Pages 11 to 12 |
|  | 6b | Any changes to trial outcomes after the trial commenced, with reasons | NA |
| Sample size | 7a | How sample size was determined | Page 11 |
|  | 7b | When applicable, explanation of any interim analyses and stopping guidelines | NA |
| Randomisation: |  |  |  |
| Sequence generation | 8a | Method used to generate the random allocation sequence | Page 10 (ref. Parent KEEPS) |
|  | 8b | Type of randomisation; details of any restriction (such as blocking and block size) | Page 10 (ref. Parent KEEPS) |
| Allocation concealment mechanism | 9 | Mechanism used to implement the random allocation sequence (such as sequentially numbered containers), describing any steps taken to conceal the sequence until interventions were assigned | Page 10 and Ref to Parent KEEPS |
| Implementation | 10 | Who generated the random allocation sequence, who enrolled participants, and who assigned participants to interventions | Page 10 and Ref to Parent KEEPS |
| Blinding | 11a | If done, who was blinded after assignment to interventions (for example, participants, care providers, those assessing outcomes) and how | Page 9 (double blinded) |
|  | 11b | If relevant, description of the similarity of interventions | NA |
| Statistical methods | 12a | Statistical methods used to compare groups for primary and secondary outcomes | Pages 12 to 13 |
|  | 12b | Methods for additional analyses, such as subgroup analyses and adjusted analyses | Page 13 |
| Results | | | |
| Participant flow (a diagram is strongly recommended) | 13a | For each group, the numbers of participants who were randomly assigned, received intended treatment, and were analysed for the primary outcome | Page 9 and Figure 1 |
|  | 13b | For each group, losses and exclusions after randomisation, together with reasons | Pages 14 to 15, and Table 2 |
| Recruitment | 14a | Dates defining the periods of recruitment and follow-up | Page 9 |
|  | 14b | Why the trial ended or was stopped | Page 9 |
| Baseline data | 15 | A table showing baseline demographic and clinical characteristics for each group | Table 1 |
| Numbers analysed | 16 | For each group, number of participants (denominator) included in each analysis and whether the analysis was by original assigned groups | Page 14 to 15 |
| Outcomes and estimation | 17a | For each primary and secondary outcome, results for each group, and the estimated effect size and its precision (such as 95% confidence interval) | Table 3 |
|  | 17b | For binary outcomes, presentation of both absolute and relative effect sizes is recommended | NA |
| Ancillary analyses | 18 | Results of any other analyses performed, including subgroup analyses and adjusted analyses, distinguishing pre-specified from exploratory | Pages 15 to 16 and Table 3 |
| Harms | 19 | All important harms or unintended effects in each group (for specific guidance see CONSORT for harms) | Ref KEEPS paper Page 15 |
| Discussion | | | |
| Limitations | 20 | Trial limitations, addressing sources of potential bias, imprecision, and, if relevant, multiplicity of analyses | Pages 20 to 21 |
| Generalisability | 21 | Generalisability (external validity, applicability) of the trial findings | Page 20 |
| Interpretation | 22 | Interpretation consistent with results, balancing benefits and harms, and considering other relevant evidence | Page 21 |
| Other information | | |  |
| Registration | 23 | Registration number and name of trial registry | Page 6** |
| Protocol | 24 | Where the full trial protocol can be accessed, if available | Page 30 |
| Funding | 25 | Sources of funding and other support (such as supply of drugs), role of funders | Pages 31 to 32 |

*We strongly recommend reading this statement in conjunction with the CONSORT 2010 Explanation and Elaboration for important clarifications on all the items. If relevant, we also recommend reading CONSORT extensions for cluster randomised trials, non-inferiority and equivalence trials, non-pharmacological treatments, herbal interventions, and pragmatic trials. Additional extensions are forthcoming: for those and for up to date references relevant to this checklist, see [www.consort-statement.org](http://www.consort-statement.org/).

**NOTE: While the KEEPS Trial has 2 ClinicalTrials.gov registration numbers, there was only one study protocol.

The parent KEEPS trial was registered with ClinicalTrials.gov on September 7^th^, 2005. At that time, KEEPS-Cog was an ancillary study of the parent KEEPS trial. Additional funding was obtained (R01 AG029624) from the NIH-NIA on 4/15/2007. We were required by our funding organization to obtain a second trial registration number; thus we registered KEEPS-Cog with ClinicalTrials.gov on February 14, 2008.

Of note, the KEEPS-Cog followed the same study protocol as that described for the parent KEEPS trial, and all subjects enrolled in KEEPS Cog were recruited from the parent KEEPS trial. No participant was allowed to participate in the cognitive study independent of enrollment in the parent KEEPS.
