# Supplementary material for: Effects of Hormone Therapy on Cognition and Mood in Recently Postmenopausal Women: Findings from the Randomized, Controlled KEEPS–Cognitive and Affective Study
Source: PLoS Med. 2015 Jun 2;12(6):e1001833. doi: 10.1371/journal.pmed.1001833 (PMC4452757; doi:10.1371/journal.pmed.1001833)
Supplement: S3 Text — (PDF) [file pmed.1001833.s006.pdf]

S3 Text: Supplementary Information about LME Models.

The fitted model can be expressed as

Level 1 (Time)

$$Y_{ij} = \theta_{0j} + \theta_{1j} Time_{ij} + r_{ij}, \quad where \ r_{ij} \sim N(0, \sigma^2) \quad and$$

Level 2

$$\theta_{0j} = \beta_{00} + \beta_{01} Education_j + \beta_{02} Age_j + \beta_{03} tE2_j + \beta_{04} oCEE_j + \zeta_{0j}$$

$$\theta_{1j} = \beta_{10} + \beta_{11} Education_j + \beta_{12} Age_j + \beta_{13} tE2_j + \beta_{14} oCEE_j + \zeta_{1j} \quad where \begin{pmatrix} \zeta_{0j} \\ \zeta_{1j} \end{pmatrix} \sim N\left[\begin{pmatrix} 0 \\ 0 \end{pmatrix}, \begin{pmatrix} \omega_{00} & \omega_{01} \\ \omega_{10} & \omega_{11} \end{pmatrix}\right]$$

The full mixed model used in this study is given by:

$$Y_{ij} = \beta_{00} + \beta_{01} Education_j + \beta_{02} Age_j + \beta_{03} tE2_j + \beta_{04} oCEE_j + \beta_{10} Time_{ij} + \beta_{11} Education_j * Time_{ij} + \beta_{12} Age_j * Time_{ij} +$$
$$\beta_{13} tE2_j * Time_{ij} + \beta_{14} oCEE_j * Time_{ij} + \zeta_{1j} * Time_{ij} + \zeta_{0j} + r_{ij}$$

The estimates of interest, and those reported in the results, are the interaction terms $\beta_{13} tE2_j * Time_{ij}$ and $\beta_{14} oCEE_j * Time_{ij}$ using the placebo group as reference. Our primary interest was to compare the effect of MHT vs placebo (t-E2 vs placebo, and o-CEE vs placebo) on changes in mood and cognitive performance across time (where time represents months since baseline) after controlling for baseline age and education level."

Cognitive Factor and Mood data were conducted as separate analyses with separate models so that we could optimize availability of data. Conducting analyses separately using the same analytical approach, was done so that women with only one set of data could be included in the appropriate analyses. For example, mood analyses included data from some women who provided data for the POMS outcomes, but not the cognitive outcomes.

We modeled time/visits in months since baseline units, e.g., 0 (baseline), Month 18, Month 36, and Month 48. In our equations, which are now included in the manuscript, the variable "Time" represents the time in months since baseline, e.g., 18 months (data associated with Month 18). The estimates of interest, and those reported in the results, are the interaction terms of time by treatment with placebo as the reference group. Site was included in the models as a random effect.

Additionally, we examined the intraclass correlation (the amount of between center variability) as a preliminary step to estimate the clustering effect and the degree of potential bias.

As stated above, treatment by time was included in the models as an interaction term $\beta_{13} tE2_j * Time_{ij}$ and $\beta_{14} oCEE_j * Time_{ij}$ using the placebo group as reference. Our primary goal was to examine the effect of hormone therapy (t-E2 vs placebo and o-CEE vs placebo) on changes in outcomes over time.
