# Supplementary material for: Effects of Hormone Therapy on Cognition and Mood in Recently Postmenopausal Women: Findings from the Randomized, Controlled KEEPS–Cognitive and Affective Study
Source: PLoS Med. 2015 Jun 2;12(6):e1001833. doi: 10.1371/journal.pmed.1001833 (PMC4452757; doi:10.1371/journal.pmed.1001833)
Supplement: S4 Text — (PDF) [file pmed.1001833.s007.pdf]

S4 Text: Clinical relevance of statistically significant findings.

In addition to estimating Effect Sizes, we referred to the literature to evaluate the clinical significance of observed changes. The American Pain Society convened a panel to develop guidelines to interpret the clinical significance of outcomes used in clinical trials, and published a Consensus Paper.[1] The POMS was one of the tools discussed by the panel. The panel recommended that a change of one SEM or half of a standard deviation should be considered clinically significant. According to the panel when normative data are used as the standard, this corresponds to a 2 to 5 point change on the individual POMS subscales. In the table above we provide the baseline values for the POMS subscales including the Mean and SD, and Mean change at Month 48. By this interpretation, the o-CEE group demonstrated clinically meaningful change on Depression, Tension-Anxiety subscales and a borderline meaningful change on the Hostility subscale. By published standards the change observed on the Depression scales corresponds to a medium effect size, and the Tension – Anxiety scale changes as small to medium effect sizes.[2] These results are highly consistent with results from a meta-analysis of clinical studies, the majority of which used o-CEE [3]. Specifically, the beneficial mood effects of MHT with progesterone were estimated to correspond to an effect size of = 0.45.

Appendix B Table. Change from Baseline for POMS Sub-Scale Scores at each Visit

POMS Sub-Scale	Mean Score (SD) at Baseline for full sample	Mean Score Change for Treatment Groups at								
		18 months			36 months			48 months		
		PL	t-E2	o-CEE	PL	t-E2	o-CEE	PL	t-E2	o-CEE
Tension-Anxiety	6.25 (4.94)	-0.704	-0.593	-1.357	-0.388	-0.491	-1.657	-1.779	-1.745	-2.694
Depression-Dejection	5.44 (6.61)	0.982	0.133	-0.803	0.752	0.302	-1.450	-0.803	-1.365	-3.234
Anger-Hostility	5.26 (5.41)	-0.004	-0.386	-0.258	-0.208	-0.629	-1.517	-1.289	-1.945	-2.258
Fatigue	6.52 (5.47)	0.453	-0.367	0.098	-0.443	0.006	-0.529	-1.229	-1.695	-2.196
Vigor	17.71 (6.36)	-0.383	-0.584	-0.115	-0.050	-1.346	0.333	-3.727	-3.480	-3.268
Confusion	5.11 (3.92)	-0.278	-0.506	-0.355	-0.198	0.050	-0.477	-1.189	-0.904	-1.225

1. Dworkin RH, Turk DC, Wyrwich KW, Beaton D, Cleeland CS, et al. (2008) Interpreting the clinical importance of treatment outcomes in chronic pain clinical trials: IMMPACT recommendations. J Pain 9: 105-121.
2. Cohen J (1988) Statistical power analysis for the behavioral sciences. Hillsdale, N.J.: L. Erlbaum Associates. xxi, 567 p. p.
3. Zweifel JE, O'Brien WH (1997) A meta-analysis of the effect of hormone replacement therapy upon depressed mood. Psychoneuroendocrinology 22: 189-212.
