# Supplementary material for: Effects of Hormone Therapy on Cognition and Mood in Recently Postmenopausal Women: Findings from the Randomized, Controlled KEEPS–Cognitive and Affective Study
Source: PLoS Med. 2015 Jun 2;12(6):e1001833. doi: 10.1371/journal.pmed.1001833 (PMC4452757; doi:10.1371/journal.pmed.1001833)
Supplement: S5 Text — (PDF) [file pmed.1001833.s008.pdf]

THE UNIVERSITY of WISCONSIN MADISON

Health Sciences IRB

4/23/2014

**Submission ID number:**	H-2005-0059-CR005
**Title:**	KEEPS Cognitive and Affective Study
**Principal Investigator:**	SANJAY ASTHANA
**Point-of-contact:**	KRISTIN ALDRIDGE
**IRB Staff Reviewer:**	JESSICA JOHNSON

A designated HS IRB member conducted an expedited review of the above-referenced continuing review progress report form. The study was approved by the IRB member for the period of 12 months with the expiration date of 4/22/2015. The study qualified for expedited review pursuant to 45 CFR 46.110 and, if applicable, 21 CFR 56.110 and 38 CFR 16.110:

Category 8: The study was previously approved by the convened IRB and the remaining research activities are limited to data analysis

To access the materials approved by the IRB, including any stamped consent forms and recruitment materials, please log in to your ARROW account and view the documents tab in the submission's workspace.

Please review the Investigator Responsibilities guidance (http://go.wisc.edu/m0lovn.), which includes a description of IRB requirements for submitting continuing review progress reports, changes of protocol and reportable events.

Please contact the appropriate IRB office with general questions: Health Sciences IRBs at 608-263-2362 or Education and Social/Behavioral Science IRB at 608-263-2320. For questions related to this submission, contact the assigned staff reviewer.
